# Supplementary material for: Effects of risperidone on amino acid metabolism, glucose, and kidney function in healthy adults: A pilot randomized controlled trial
Source: PLoS One. 2025 Dec 5;20(12):e0324222. doi: 10.1371/journal.pone.0324222 (PMC12680216; doi:10.1371/journal.pone.0324222)
Supplement: S1 Protocol — (PDF) [file pone.0324222.s001.pdf]

# リスペリドンが健常者の糖代謝指標と腸内環境に及ぼす影響についての

# 非盲検ランダム化比較試験

# 研究実施計画書

略称：リスペリドン健常者パイロット試験

研究責任医師：金沢大学附属病院検査部特任助教
大島 恵
研究事務局：金沢大学大学院腎臓内科学事務室
住所〒920-8641 石川県金沢市宝町 13-1
電話：076-265-2499
FAX：076-234-4273
E-mail：lab-med@med.kanazawa-u.ac.jp

2021 年 12 月 1 日 計画書案 Ver. 0.1
2022 年 2 月 8 日 計画書案 Ver. 0.2
2022 年 3 月 9 日 臨床研究審査委員会承認版 Ver. 1.0
2022 年 11 月 11 日 Ver. 1.1
2023 年 1 月 18 日 Ver. 1.2

## 目次


0. 研究の概要 ........ 4
0.1. シェーマ ........ 4
0.2. 臨床研究デザインの要約 ........ 4
0.3. 臨床研究の種類 ........ 4
1. 目的 ........ 4
2. 背景 ........ 4
2.1. 国内外における対象疾患の状況（対象疾患に関する疫学データを含む） ........ 4
2.2. これまでに実施されてきた標準治療の経緯及び内容 ........ 4
2.3. 現在の標準治療及び治療成績 ........ 5
2.4. 当該臨床研究の必要性につながる、現在の標準治療の課題、不明点等 ........ 5
2.5. プロトコール治療による利益と不利益 ........ 6
2.5.1. 研究対象者に生じる負担並びに予測されるリスク ........ 6
2.5.2. 研究対象者に予想される利益 ........ 6
2.5.3. これらの総合評価並びに負担及びリスクを最小化する対策 ........ 6
2.6. 研究デザイン ........ 6
2.7. 被験薬の概要 ........ 6
3. 適格基準 ........ 7
3.1. 選択基準 ........ 7
3.2. 除外基準 ........ 7
4. 登録・割付 ........ 7
4.1. 症例登録の手順 ........ 7
4.2. 割付方法と割付調整因子 ........ 8
5. 臨床研究計画 ........ 8
5.1. プロトコール治療 ........ 8
5.1.1. 投薬群・手術群・検査群等 ........ 8
5.1.2. 投薬・手術・検査等の介入を行う手順と経時的なスケジュール ........ 8
5.1.2.1. 投薬部位・手術部位・検査部位等 ........ 8
5.1.2.2. 投薬・手術・検査等の介入を行う時期・期間 ........ 8
5.1.2.3. 用法・用量、回数、所要時間等 ........ 8
5.2. 用量・スケジュール変更基準 ........ 9
5.3. 併用治療・支持療法 ........ 9
5.4. プロトコール治療の中止 ........ 9
5.5. 後治療 ........ 9
5.6. 被験薬等の管理の手順 ........ 9
5.7. 試料・情報等の保存及び他の機関等の試料・情報等の利用 ........ 9
6. 観察・検査・報告項目とスケジュール ........ 10
6.1. 観察・検査項目及び報告すべき治療情報 ........ 10
6.2. 観察・検査・報告スケジュール ........ 10

7. 目標症例数と臨床研究期間 ........ 11
7.1. 目標症例数 ........ 11
7.2. 臨床研究期間 ........ 11
8. 有害事象（疾病等）の評価・報告 ........ 11
8.1. 有害事象（疾病等）の定義 ........ 11
8.2. 有害事象の評価と報告 ........ 12
8.3. 予期される有害事象 ........ 12
8.4. 重篤な有害事象の報告と対応 ........ 12
8.4.1. 当該臨床研究の実施で発生した重篤な有害事象の報告 ........ 12
8.4.2. 厚生労働大臣等への報告 ........ 13
8.4.3 詳細報告及び追加報告 ........ 13
9. エンドポイントの定義 ........ 13
9.1. 主要エンドポイント ........ 13
9.2. 副次エンドポイント ........ 13
10. 統計学的考察 ........ 14
10.1. 目標症例数の設定根拠 ........ 14
10.2. 解析対象集団 ........ 14
10.3. 解析項目・方法 ........ 14
10.3.1. 主要エンドポイントの主たる解析方法 ........ 14
10.3.2. 主要エンドポイントの主たる副次解析方法 ........ 14
10.3.3. 副次エンドポイントの解析方法 ........ 15
10.3.4. 有意水準 ........ 15
10.3.5. 欠測データの取扱い等 ........ 15
10.4. 中間解析 ........ 15
10.5. 統計解析計画の変更に関する手順 ........ 15
11. 症例報告書の記入と提出 ........ 15
11.1. 種類と提出期限 ........ 15
11.2. 記入方法 ........ 15
11.3. 送付方法 ........ 16
12. 原資料等の閲覧と品質管理及び品質保証 ........ 16
12.1. 直接閲覧の受入れと協力 ........ 16
12.2. モニタリング ........ 16
13. 倫理的事項 ........ 16
13.1. 遵守すべき諸規則 ........ 16
13.2. 研究開始に係る認定臨床研究審査委員会による審査と実施医療機関の管理者、厚生労働大臣への届出 ........ 16
13.3. 説明文書・同意文書（様式）の作成と改訂 ........ 16
13.4. インフォームド・コンセント ........ 17
13.5. 試料・情報の二次利用 ........ 18

14. 個人情報の取扱い ..... 18
15. 研究計画書の逸脱・変更・改訂 ..... 18
15.1. 研究計画書の逸脱又は変更 ..... 18
15.2. 研究計画書の改訂 ..... 18
16. 臨床研究の終了と早期中止 ..... 19
16.1. 臨床研究の終了 ..... 19
16.2. 臨床研究の早期中止 ..... 19
17. 実施医療機関の管理者等への報告 ..... 20
17.1. 実施医療機関の管理者への報告事項 ..... 20
17.2. 認定臨床研究審査委員会への報告事項 ..... 20
17.3. 厚生労働大臣への報告事項 ..... 21
18. 臨床研究に関する記録の取り扱い ..... 21
19. 臨床研究の実施に係る金銭の支払及び補償 ..... 22
19.1. 臨床研究に関する費用 ..... 22
19.2. 健康被害に対する補償 ..... 22
20. 研究資金及び利益相反管理 ..... 22
20.1. 利益相反管理 ..... 22
20.2. 研究資金源 ..... 22
21. 研究成果の帰属と結果の公表 ..... 22
22. 研究組織 ..... 22
22.1. 研究責任医師 ..... 22
22.2. 研究事務局（調整管理実務担当者） ..... 22
22.3. データマネジメント責任者 ..... 23
22.4. 統計解析責任者 ..... 23
22.5. モニタリング責任者 ..... 23
22.6. 被験薬管理者 ..... 23
22.7. 症例登録センター ..... 23
22.8. 検体検査測定センター ..... 23
23. 苦情及び相談窓口 ..... 24
24. 文献 ..... 24
25. 付録 ..... 24

# 0. 研究の概要

## 0.1. シェーマ

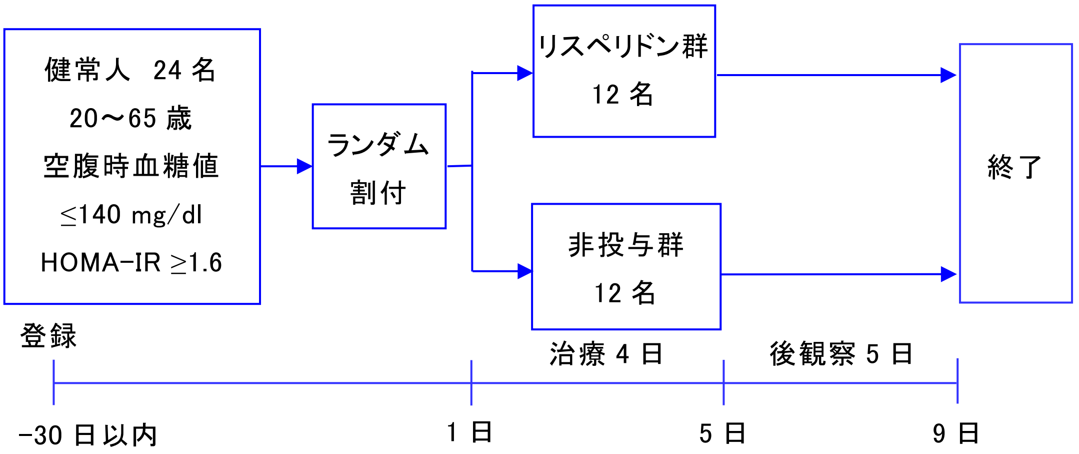


## 0.2. 臨床研究デザインの要約

前向き、介入研究、特定臨床研究、無治療対照、ランダム化（最小化法）、非盲検、単施設

## 0.3. 臨床研究の種類

特定臨床研究（企業等からの資金提供なし、適応外使用あり）

# 1. 目的

本研究は、健常成人を対象としてリスペリドンの服用による糖代謝指標と腸内環境への影響について評価するための無治療対照非盲検ランダム化比較試験である。

主要エンドポイントはインスリン抵抗性の変化、副次エンドポイントは腸内細菌叢の変化とする。

# 2. 背景

## 2.1. 国内外における対象疾患の状況（対象疾患に関する疫学データを含む）

本邦の慢性腎臓病（CKD）患者は約 1,400 万人と推計される。CKD は進行すると末期腎不全に至るのみならず心血管疾患の発症および死亡につながるため、その進行の抑制は重要な課題である。特に糖尿病を背景とする糖尿病性腎臓病は、依然として透析導入の原疾患の第 1 位（41.6%）であり（わが国の慢性透析療法の現況 2019 年）、心血管疾患の発症や生命予後の観点からも最も重要な疾患である。

## 2.2. これまでに実施されてきた標準治療の経緯及び内容

糖尿病性腎臓病に対する治療として、厳格な血糖あるいは血圧管理の重要性が、1990 年代に行われた DCCT/EDIC、Kumamoto 研究、UKPDS 等により示されている。2000 年代には降圧薬レニン・アンジオテンシン系（RAS）阻害薬を用いた大規模臨床試験により、RAS 阻害薬の腎保護効果、特にアルブミン尿の減少効果が明らかになり、その結果 CKD 及び高血圧のガイドラインで糖尿病患者の降圧薬の第一選択薬として RAS 阻害薬が推奨された。2010 年代には糖尿病治療薬の DPP-4（dipeptidyl peptidase-4）阻害薬、GLP-1（glucagon-like peptide-1）受容体作動薬、SGLT2（sodium-glucose cotransporter 2）阻害薬等による腎保護効果が様々な大規模臨床試験により検証され、糖尿

病性腎臓病の治療方針が変わりつつある。

### 2.3. 現在の標準治療及び治療成績

上記の背景より、「エビデンスに基づくCKD診療ガイドライン2018」では糖尿病を背景とするCKDの進行を抑制するために血糖・血圧の適切な管理を含む集約的治療が推奨されている。一方、2型糖尿病を伴うCKDを対象とした大規模臨床試験であるCREDENCE試験では、RAS阻害薬に加えてSGLT2阻害薬を服用しても、末期腎不全に至る患者が年間49人に1人と概算され、残余リスクをいかに減らすかが重要である。

### 2.4. 当該臨床研究の必要性につながる、現在の標準治療の課題、不明点等

現行治療にもかかわらず糖尿病を背景として末期腎不全に至る患者をいかに減らすかが、医療費の適正化および生命予後の観点からも重要な課題となっている。

近年、腎臓病において腸内細菌叢が変化し、腎機能に影響を及ぼすことが知られており、その代謝産物の一つとしてアミノ酸が注目されている(1)。近年の分析技術の向上により、アミノ酸は光学異性体であるL体とD体に分離し測定することが可能となった。これまで急性腎障害のマウスでD-アミノ酸のうち血中D-セリンが上昇し腎機能と相関を示すこと、またD-セリン投与により腎障害が軽減することが明らかになっている（2）。D-アミノ酸を標的とした腎臓病に対するバイオマーカーや治療薬開発への活用が期待される。

リスペリドンはD-アミノ酸分解酵素（D-amino acids oxidase: DAO）の阻害作用を有する代表的な薬剤であり、D-アミノ酸の受容体であるNMDAR（Nmethyl-D-asparate receptor: NMDAR）の機能低下をきたす精神疾患である統合失調症の治療薬として既に承認されている。また糖尿病性腎症のマウスでリスペリドン投与によりインスリン抵抗性の改善を認めている（論文執筆中）。したがってリスペリドンのD-アミノ酸分解酵素の阻害作用は耐糖能の改善を介して腎保護につながることが示唆されるが、ヒトでの検証はされておらず、また腎機能障害患者での安全性や腸内細菌叢への影響も解明されていない。

以上の背景に基づき、本研究ではインスリン抵抗性を有する健常成人を対象として、リスペリドンの服用による糖代謝指標と腸内環境への影響について評価する。インスリン抵抗性の指標として、HOMA-IRは空腹時の血糖が140 mg/dL以下の場合はグルコースクランプ法で求めたインスリン抵抗性とよく相関することが報告されていることから、HOMA-IR＞1.6を用いる。

本研究によりリスペリドンによる耐糖能及び腸内環境への影響、安全性が明らかになれば、次の段階として糖尿病性腎臓病患者を対象としたリスペリドン投与の試験を計画する予定である。

【用法用量の設定根拠】

日本人及び白人の健康成人各24例を対象に、リスペリドン錠1 mg単回投与（1日目）及び反復投与（5～11日目）時の薬物動態、安全性及び忍容性の比較検討を目的として実施された第I相薬物動態試験（RIS-P01-101 試験）では、安全性解析対象集団における有害事象の発現割合は日本人群及び白人群の両群100%（24/24 例）であった（付録1）。日本人群で多く報告された有害事象は、傾眠（24/24 例、100%）、起立性低血圧（9/24 例、38%）、浮動性めまい（8/24 例 33%）であった。なお、死亡及びその他の重篤な有害事象は両群で認めなかった。

動物実験において反復投与によりインスリン抵抗性の改善を認めたことから、単回投与では目標を達成できないため、本研究では安全性に配慮した上で反復投与を行う。投与量については安全性に配慮し、最低用量である0.5 mg/日を用いる。またリスペリドンの保険適用がある統合失調症は本研

究の対象疾患ではない。

## 2.5. プロトコール治療による利益と不利益

### 2.5.1. 研究対象者に生じる負担並びに予測されるリスク

対象者には採血の負担および試験薬による有害事象のリスクがある。経済的負担は発生しない。

### 2.5.2. 研究対象者に予想される利益

研究対象者には謝礼を支払う。研究成果により医療の進歩に貢献できる可能性がある。

### 2.5.3. これらの総合評価並びに負担及びリスクを最小化する対策

対象者への負担を最小化するため、採血は必要最小量・回数とする。試験薬リスペリドンの投与により眠気、注意力・集中力・反射運動能力等の低下が起こることがあることから、眠前に投与し、服用中は自動車の運転等の危険な機械の操作に従事しない、また飲酒を控えるよう指示する。当該臨床研究に起因した健康被害が発生した場合には適切な治療を行い、医薬品副作用被害救済制度でいう死亡・後遺障害 1 級及び 2 級に該当する健康被害が発生した場合は、当該臨床研究グループが金沢大学附属病院の 2021 年度臨床研究等に係る公募研究助成により加入している「臨床研究に係る補償責任保険」にて対応する。

## 2.6. 研究デザイン

当該臨床研究は、健常成人におけるリスペリドンの糖代謝指標と腸内環境に及ぼす影響についての前向き非盲検ランダム化比較試験である。登録後、年齢及び性別を割付調整因子としてとして最小化法を用い、研究対象者を試験薬投与（リスペリドン）群又は非投与群に 1:1 にランダムに割り付ける。割付後、試験薬リスペリドンを 4 日間服用し（プロトコール治療期）、HOMA-IR 値の変化量を評価する。なお、プロトコール治療期は非盲検下で実施する。終了後は 5 日間の後観察期をおいて、有害事象の有無を確認する。

以上、研究対象者の安全性に考慮しつつ、バイアスを最小限にする工夫した上で、「世界医師会ヘルシンキ宣言」及び臨床研究法に準じて研究を実施する。

## 2.7. 被験薬の概要

・被験薬名：リスペリドン （商品名リスパダール®）

・製造元（又は販売元）：ヤンセンファーマ株式会社

・薬効分類：非定型抗精神病薬（セロトニン・ドパミン拮抗薬）

・作用機序：主としてドパミン D2 受容体拮抗作用及びセロトニン 5-HT2 受容体拮抗作用に基づく、中枢神経系の調節によるものと考えられる

・適応症：統合失調症、小児期の自閉スペクトラム症の易刺激性

・投与経路、用法・用量、使用方法：統合失調症では通常、成人にはリスペリドンとして 1 回 1mg1 日 2 回より経口投与を開始し、徐々に増量する。維持量は通常 1 日 2～6mg を原則として 1 日 2 回に分けて経口投与する。

・禁忌：昏睡状態の患者、バルビツール酸誘導体等の中枢神経抑制剤の強い影響下にある患者、アドレナリンを投与中の患者、本剤の成分及びパリペリドンに対し過敏症の既往歴のある患者

・主な臨床使用成績：国内で実施された二重盲検比較試験を含む総計 727 例の統合失調症及び統合失調感情障害患者における臨床試験の結果、有効性評価対象症例 722 例に対する中等度改善以上の改善率は 51.5%（372/722 例）であった。また、二重盲検比較試験によって統合失調症に対する

本剤の有用性が認められている。安全性評価対象症例 723 例中 420 例（58.1%）に副作用が認められた。主なものは、アカシジア 126 例（17.4%）、振戦 95 例（13.1%）、易刺激性 92 例（12.7%）、不眠症 87 例（12.0%）、筋固縮 85 例（11.8%）、流涎過多 81 例（11.2%）であった。

・副作用・不具合：

1 重大な副作用：悪性症候群、遅発性ジスキネジア、麻痺性イレウス、抗利尿ホルモン不適合分泌症候群（SIADH）、肝機能障害、黄疸、横紋筋融解症、不整脈、脳血管障害、高血糖、糖尿病性ケトアシドーシス、糖尿病性昏睡、低血糖、無顆粒球症、白血球減少、肺塞栓症、深部静脈血栓症、持続勃起症

2 その他の副作用：添付文書（付録 2）参照

・相互作用：添付文書参照

・使用上の注意事項：添付文書参照

# 3. 適格基準

## 3.1. 選択基準

下記を全て満たす者を対象とする。

1) 20 歳以上 65 歳以下の男女
2) 精神疾患、神経疾患またはそれらの既往歴がない者
3) 空腹時血糖値 140 mg/dL 未満
4) HOMA-IR（homeostasis model assessment-insulin resistance）1.6 以上
5) 推算 GFR 60 ml/min/1.73 $m^2$ 以上
6) AST 及び ALT 30 IU/L 未満
7) 不整脈の既往歴及び先天性 QT 延長症候群のない者
8) 活動性がん（無病期間 5 年以内）の既往のない者
9) 4 日に 1 回以上排便のある者（便秘薬使用の有無は問わない）
10) 本研究への参加にあたり十分なインフォームド・コンセントの後に、本人の自由意思による文書同意が得られている

## 3.2. 除外基準

1) 医学的診断のもと治療中の疾病を有する者
2) リスペリドンの成分及びパリペリドンに対し過敏症の既往歴のある者
3) 妊娠または授乳中の者
4) 被験薬投与開始前 3 か月以内に他の臨床研究に参加した者
5) 研究責任医師又は研究分担医師が研究対象者として不適当と判断した者
6) 研究責任医師と同じもしくは関連する教室の教職員

[設定根拠] 1)5)6)有効性評価への影響および安全性への配慮のため、4)有効性評価への影響のため 2)3)4)安全性への配慮のため、

# 4. 登録・割付

## 4.1. 症例登録の手順

当該臨床研究の症例登録は研究責任医師あるいは研究分担医師が以下の手順で行う。

金沢大学教職員に対して金沢大学アカンサスポータル等により周知を行い、本人の自由意思による研究参加希望者を募る。

研究責任医師と同じもしくは関連する教室の教職員を除く参加希望者に、当該臨床研究についての説明を行い、十分に考える時間を与え、研究対象者が臨床研究の内容をよく理解したことを確認した上で、臨床研究への参加について依頼し、文書による同意を取得する。

1) 研究責任医師あるいは研究分担医師は同意取得後にスクリーニング検査を行い、対象患者が適格基準をすべて満たし、除外基準のいずれにも該当しないことを確認し、研究責任医師が保管する研究対象者リストに研究対象者と研究対象者識別コードを対応させる必要事項（同意取得日、研究対象者識別コード、研究対象者名、カルテ番号等）を記載する。
2) 研究責任医師あるいは研究分担医師は研究対象者識別コードを用いた症例登録書を作成し、症例登録センターに FAX 送信する。
3) 症例登録センターより登録番号および割付群が記載された症例登録確認書が、症例登録票に記載された返信用 FAX 宛に送信されるので、研究責任医師あるいは研究分担医師はこの症例登録確認書を受領後、臨床研究を開始する。
4) 同意撤回、中止、脱落等が生じたときは、速やかに研究責任医師に報告する。
5) 誤登録、重複登録等が生じた時は、速やかに登録センターへ報告する。

### 4.2. 割付方法と割付調整因子

症例登録センターは、症例登録票の記載内容により適格性を確認し、割付を行う。割付に際しては①年齢、②性別を割付調整因子とする最小化法を用いて原則 1:1 になるよう動的割付を行う。

## 5. 臨床研究計画

### 5.1. プロトコール治療

#### 5.1.1. 投薬群・手術群・検査群等

・リスペリドン群（12 症例）

・非投与群（12 症例）

#### 5.1.2. 投薬・手術・検査等の介入を行う手順と経時的なスケジュール

##### 5.1.2.1. 投薬部位・手術部位・検査部位等

リスペリドン群はリスペリドンを経口投与する。

##### 5.1.2.2. 投薬・手術・検査等の介入を行う時期・期間

リスペリドン群の投薬期間は 4 日間である。

##### 5.1.2.3. 用法・用量、回数、所要時間等

リスペリドン群は登録後 30 日以内にリスペリドン 0.5mg（リスパダール OD 0.5mg）を 1 日 1 回、眠前、4 日間内服を開始する。リスペリドン群は投薬終了後に残薬を持参し回収する。投与量については安全性に配慮し、最低用量である 0.5 mg/日を用いる。

全ての対象者は 1 日目、5 日目、9 日目（又は中止時）に来院する。投薬 1 週間前から後観察の間は、腸内細菌に影響を与えるプロバイオティクスを含む食品（ヨーグルト、乳酸菌飲料、納豆、味噌、ぬか漬け、キムチなどの発酵食品）の摂取を制限する。投薬中は有害事象のリスクを減らすため自動車の運転等の危険な機械の操作に従事しない、また飲酒しないよう指示する。入院は行わない。

また対象者には取り決め事項の遵守状況を研究責任医師または研究分担医師が問診で確認する。

### 5.2. 用量・スケジュール変更基準

- 登録・割付後に治療を要する疾病を認めた場合は疾病の治療が終了するまで投薬を延期する。
- 試験薬リスペリドンは最低用量を用いるため、減量は行わず、投薬を継続できない有害事象を認めた場合は中止する。

### 5.3. 併用治療・支持療法

併用禁止治療：適応症あるいは作用機序が同じ薬剤（抗精神病薬）、神経系障害の副作用が同じ薬剤（催眠鎮静剤、抗不安剤）、添付文書上併用禁忌と記載されている薬剤（アドレナリン）の併用を禁止する。有効性に影響を及ぼす血糖降下薬（SGLT2 阻害薬を含む）の試験期間中の併用を禁止する。腸内細菌叢に影響を及ぼす抗菌薬、乳酸菌製剤の試験期間中の併用を禁止する。

併用制限治療：投薬 1 週間前から後観察の間は、腸内細菌に影響を与えるプロバイオティクスを含む食品（ヨーグルト、乳酸菌飲料、納豆、味噌、ぬか漬け、キムチなどの発酵食品）の摂取を制限する。添付文書上併用注意と記載されている薬剤（中枢神経抑制剤、ドパミン作動薬、降圧薬、アルコール、CYP2D6 を阻害する薬剤（パロキセチン等）、CYP3A4 を誘導あるいは阻害する薬剤（リファンピシン、イトラコナゾール等）、QT 延長を起こすことが知られている薬剤）の試験期間中の併用には注意する。

### 5.4. プロトコール治療の中止

研究責任医師または研究分担医師は、以下の理由で臨床研究の継続が不可能と判断した場合には、被験薬の投与を中止する。中止の日付、理由、経過をカルテならびに症例報告書（CRF）に記載するとともに、中止・脱落時点で必要な検査を行い、有効性・安全性の評価を行う。有害事象発生により中止した場合には、可能な限り現状に回復するまでフォローアップする。

研究対象者からの臨床研究参加の辞退の申し出や同意の撤回があった場合
登録後に適格性を満足しないことが判明した場合
合併症により臨床研究の継続が困難な場合
有害事象により臨床研究の継続が困難な場合
妊娠が判明した場合
転居等により研究対象者が来院しない場合
臨床研究全体が中止された場合
その他の理由により、研究責任者が臨床研究を中止することが適当と判断した場合

### 5.5. 後治療

後治療は規定しない。

### 5.6. 被験薬等の管理の手順

研究責任医師は被験薬の購入状況（購入数、購入日等）を確認後、被験薬管理者を指名する。被験薬管理者は処方状況（処方した研究対象者の識別番号、処方量、処方日等）、廃棄又は未使用品の扱い等を管理する。なお、被験薬は添付文書に記載のある条件で保存し、本研究以外の目的には使用しない。

### 5.7. 試料・情報等の保存及び他の機関等の試料・情報等の利用

研究責任医師は、定められた保管方法に従って研究分担医師が適切に保管するよう指導し試料の漏えい、混交、盗難、紛失等が起こらないよう必要な管理を行う。採取した血液は、臨床研究終了後

10 年まで腎臓内科医局にて冷凍保管する。廃棄する際は、匿名化し個人情報に注意して行う。

本研究では他機関に資料・情報の提供は行わない。また研究対象者の健康又は子孫に受け継がれ得る遺伝的特徴等に関する重要な知見が得られる可能性はない。

# 6. 観察・検査・報告項目とスケジュール

## 6.1. 観察・検査項目及び報告すべき治療情報

・登録時

研究対象者情報：生年月日、性別、既往症、併発症

血液検査（原則空腹時もしくは食後 6 時間採血）：空腹時血糖、インスリン、クレアチニン（Cre）、AST、ALT

生理検査：心電図

＊Cre、AST、ALT、心電図に関しては、直近 1 年以内の検査歴がある場合は研究対象者が持参した検査結果を研究責任医師又は研究分担医師が確認し代用可能とする。

・投薬開始前

研究対象者情報：識別コード、身長・体重、血圧、脈拍、生活習慣（現在喫煙・飲酒の有無）

血液検査（原則空腹時もしくは食後 6 時間以上経過したあとの採血）：白血球数、赤血球数、ヘモグロビン、ヘマトクリット、血小板数、TP、Alb、AST、ALT、BUN、Cre、UA、Na、K、Ca、P、Mg、空腹時血糖、HbA1c、T-Cho、HDL-Cho、TG、インスリン、C ペプチド

早朝尿検査：定性（糖・潜血）、定量（Alb、Cre）

特殊検査：キラルアミノ酸（血液・尿・便・唾液）、腸内細菌 16S rRNA 菌叢解析（便・唾液）

＊採便は登録日以降で投薬開始までに採取する。

・投薬終了時・中止時

研究対象者情報：体重、血圧、脈拍

血液検査（原則空腹時もしくは食後 6 時間採血）：白血球数、赤血球数、ヘモグロビン、ヘマトクリット、血小板数、TP、Alb、AST、ALT、BUN、Cre、UA、Na、K、Ca、P、Mg、空腹時血糖、HbA1c、T-Cho、HDL-Cho、TG、インスリン、C ペプチド

早朝尿検査：定性（糖・潜血）、定量（Alb、Cre）

特殊検査：キラルアミノ酸（血液・尿・便・唾液）、腸内細菌 16S rRNA 菌叢解析（便・唾液）

生理検査：心電図

有害事象の有無・服薬状況

＊採便は投薬終了（又は中止）以降で後観察時までに採取する。

・後観察時

研究対象者情報：体重、血圧、脈拍

血液検査（原則空腹時もしくは食後 6 時間採血）：Cre、空腹時血糖、HbA1c、インスリン、C ペプチド

早朝尿検査：定量（Alb、Cre）

有害事象の有無

＊問診、診察、採血は金沢大学大学院腎臓内科学の研究室または金沢大学保健管理センターで実施する。採尿は同日の早朝尿を採取する。

## 6.2. 観察・検査・報告スケジュール

| 項目 | 登録 | 投薬開始 | 投薬終了 | 後観察 | 中止時 |
| --- | --- | --- | --- | --- | --- |
| Visit<br>時期<br>（許容範囲） | Visit 1<br>登録後 30 日以内に投薬開始 | Visit 2<br>1 日<br>（±0） | Visit 3<br>5 日<br>（±1 日） | Visit 4<br>9 日<br>（±2 日） | 中止日<br><br>（+2 日） |
| 同意取得 | ○ | | | | |
| 背景の確認 | ○ | ○ | | | |
| 試験薬リスペリドン投与 | | ← | → | | |
| 有害事象の観察 | | ← | | → | |
| 身長・体重測定 | | ○ | ○ | ○ | ○ |
| 血圧・脈拍数測定 | | ○ | ○ | ○ | ○ |
| 血液検査 | ○ | ○ | ○ | ○ | ○ |
| 尿検査 | | ○ | ○ | ○ | ○ |
| 心電図検査 | ○ | | ○ | | |
| 特殊検査（血液・尿・便・唾液） | | ○<br>（便は登録～投薬開始までに採取） | ○<br>（便は投薬終了～後観察までに採取） | | ○<br>（便は中止～後観察までに採取） |

＊問診、診察、採血、採尿、採便、採唾液、心電図検査は金沢大学大学院腎臓内科学の研究室または金沢大学保健管理センターで実施する。

# 7. 目標症例数と臨床研究期間

## 7.1. 目標症例数

参加施設数　1 施設、目標症例数 24 例（リスペリドン群 12 例、非投与群 12 例）

## 7.2. 臨床研究期間

登録期間：jRCT 初回公表日から 2024 年 3 月 31 日

観察期間：jRCT 初回公表日から 2024 年 9 月 30 日（最初の症例を登録したときから臨床研究の内容に関する事項として記載した全ての評価項目に係るデータの収集を行うための期間）

研究実施期間：jRCT 初回公表日から 2025 年 3 月 31 日（実施計画を Japan Registry of Clinical trials （jRCT）に登録してから総括報告書の概要を jRCT に登録するまでの期間）

# 8. 有害事象（疾病等）の評価・報告

## 8.1. 有害事象（疾病等）の定義

有害事象とは、実施された研究との因果関係の有無を問わず、研究対象者に生じた全ての好ましくない又は意図しない傷病若しくはその徴候（臨床検査値の異常を含む。）をいう。

「重篤な有害事象」とは、有害事象のうち、次に掲げるいずれかに該当するものをいう。

1） 死に至るもの
2） 生命を脅かすもの

3) 治療のための入院又は入院期間の延長が必要となるもの
4) 永続的又は顕著な障害・機能不全に陥るもの
5) 子孫に先天異常を来すもの

「予測できない重篤な有害事象」とは、重篤な有害事象のうち、研究計画書、インフォームド・コンセントの説明文書等において記載されていないもの又は記載されていてもその性質若しくは重症度が記載内容と一致しないものをいう。

副作用（薬物有害反応 ADR: adverse drug reaction）とは、有害事象のうち、当該医薬品の使用との因果関係が否定できないもの（医薬品の使用との因果関係で「否定できない」と判定されたもの。因果関係が不明なものも含む）をいう。

予測できない薬物有害反応とは、薬物有害反応のうち、被験薬概要書又は添付文書に記載されていないもの、あるいは記載されていてもその性質や重症度が記載内容と一致しないものをいう。

### 8.2. 有害事象の評価と報告

有害事象の評価には、MedDRA/J (Medical Dictionary for Regulatory Activities/J: ICH 国際医薬用語集日本語版)に準じて判断する。

研究責任医師又は研究分担医師は、有害事象の発現を認めた場合、厚生労働省「重篤副作用疾患別対応マニュアル」に基づいて、適切な処置を行い、症例報告書により報告するとともに、因果関係の有無にかかわらず、臨床研究期間終了まで観察し、可能な限り回復するまでその後も追跡観察する。プロトコール治療終了又は中止後 30 日以内までに研究対象者に発現した有害事象は、因果関係の有無に関わりなくすべて観察対象とする。ただし、研究対象者の合併症に伴い発現した症状が慢性化している場合、転院等で継続的な観察が困難な場合は、この限りでない。

研究責任医師は、重篤な有害事象の発生を認めたときは、研究機関の手順書に従い速やかに研究機関の長に報告する。研究責任医師は毎年 1 回、研究の進捗状況ならびに研究の実施に伴う有害事象の発生状況等を研究機関の管理者に報告する。

### 8.3. 予期される有害事象

添付文書から以下の有害事象が予期される。

・重大な有害事象（添付文書上の重大な副作用）：悪性症候群（頻度不明）、遅発性ジスキネジア（0.55%）、麻痺性イレウス（頻度不明）、抗利尿ホルモン不適合分泌症候群（SIADH）（頻度不明）、肝機能障害・黄疸（頻度不明）、横紋筋融解症（頻度不明）、不整脈（頻度不明）、脳血管障害（頻度不明）、高血糖・糖尿病性ケトアシドーシス・糖尿病性昏睡（頻度不明）、低血糖（頻度不明）、無顆粒球症・白血球減少（頻度不明）、肺塞栓症・深部静脈血栓症（頻度不明）、持続勃起症（頻度不明）

・その他添付文書上の副作用（発症割合 5%以上）：食欲不振、不眠症・不安、アカシジア・振戦・構音障害・傾眠・めまい・ふらつき、胃腸障害、筋固縮、月経障害、易刺激性・倦怠感・口渇

### 8.4. 重篤な有害事象の報告と対応

#### 8.4.1. 当該臨床研究の実施で発生した重篤な有害事象の報告

1) 研究分担医師は、重篤な有害事象が発生した場合、適切な処置を行うとともに、被験薬との因果関係を問わず、直ちに研究責任医師に報告する。
2) 研究責任医師は、当該重篤な有害事象について、当該臨床研究実施との因果関係及び既知事項か否かの判断を速やかに行い、「金沢大学における臨床研究法上の臨床研究における疾病等及び不具合等報告に関する手順書」に従い対応する。即ち、重篤な有害事象が発生し

た場合、知ったときから起算して 72 時間以内に金沢大学臨床研究電子申請システムを用いて「医薬品疾病等報告書（第 1 報）」（統一書式 8）（及び厚生労働大臣への報告が必要な重篤な有害事象の場合は「疾病等報告書（医薬品）」（別紙様式 2-1 を添付）を申請し、病院長に報告する（当該臨床研究との因果関係が疑われる事象に関しては認定臨床研究審査委員会の事務局にも提出される。）

3） 金沢大学臨床研究電子申請システムにて報告された内容を、研究事務局は金沢大学附属病院の管理者、研究責任医師、試験薬製造販売業者に報告する。

4） 他の研究責任医師は自らが所属する実施医療機関の管理者に報告する。

5） 実施医療機関の管理者は当該有害事象について必要な措置を講じる。

6） 認定臨床研究審査委員会の事務局は、認定臨床研究審査委員会の意見を研究事務局に報告し、研究事務局はその報告内容を金沢大学附属病院の管理者及び研究責任医師、被験薬製造販売業者に報告する。

7） 有害事象に関する更なる手順の詳細は各実施機関が定めた重篤な有害事象に関する手順書に従う。

＜緊急時の連絡先＞

研究責任医師：大島　恵

金沢大学附属病院　検査部

住所：石川県金沢市宝町 13-1

電話：076-265-2499

### 8.4.2. 厚生労働大臣等への報告

当該臨床研究の実施に起因すると疑われる予測できない重篤な有害事象が発生した場合、研究責任医師は、その発生を知った日から死亡又は死亡のおそれに関しては 7 日以内、それ以外は 15 日以内に「疾病等報告書（医薬品）」（別紙様式 2-1）を厚生労働大臣に提出する。

### 8.4.3 詳細報告及び追加報告

認定臨床研究審査委員会に報告した重篤な有害事象について追加の情報が得られた場合、研究責任医師は「医薬品疾病等報告書」の続報及び「疾病等報告書」の続報を認定臨床研究審査委員会に提出し、8.4.1. 3)～7)を行う。また、「疾病等報告書」の続報を厚生労働大臣に提出する。当該有害事象の転帰が「未回復」又は「不明」である場合、できる限り追跡観察を行う。

## 9. エンドポイントの定義

### 9.1. 主要エンドポイント

投与前から投与 5 日目の HOMA-IR の変化

定義：HOMA-IR の算出式

HOMA-IR＝空腹時血漿インスリン値（μU/mL）×空腹時血糖値（mg/dL）/405

設定根拠：HOMA-IR はインスリン抵抗性を表す一般的な指標として用いられる。

### 9.2. 副次エンドポイント

1） 下記項目における投与前から投与 5 日目、投与終了後 5 日目の変化

・血糖値、インスリン、HbA1c、C ペプチド

・血圧、BMI

・eGFR

eGFR の推算式

eGFR(mL/分/1.73 m$^2$)=194×血清クレアチニン濃度(mg/dL)$^{-1.094}$ ×年齢(歳)$^{-0.287}$

(女性は×0.739) 同意取得日の年齢で算出する。

・尿中アルブミン・クレアチニン比

・キラルアミノ酸(血液・尿・便・唾液)

・腸内細菌 16S rRNA 菌叢解析(便・唾液)

2) 有害事象の発現

設定根拠:血糖値、HbA1c は糖尿病患者における一般的な血糖管理の指標であり、インスリン、C ペプチドはインスリン分泌の指標として用いられる。また血圧管理不良、肥満、eGFR 低下、尿中アルブミン・クレアチニン比増加は糖尿病合併症の指標である。16SrRNA 菌叢解析は、細菌がもつ 16SrRNA 遺伝子を PCR にて増幅し次世代シーケンサーにて検体に含まれる細菌の種類や分布を解析する方法であり、腸内細菌の網羅的評価が可能である。キラルアミノ酸は腸内細菌の代謝産物であり 16SrRNA 菌叢解析と共に評価することにより、腸内環境の変化の詳細な分析が可能となる。

# 10. 統計学的考察

## 10.1. 目標症例数の設定根拠

当該臨床研究は健常者を対象としたパイロット試験であり、研究期間内での実施可能症例数として設定した。この例数のもとでは、主要評価項目である HOMA-IR の違いを検討する場合、リスペリドン群と非投与群それぞれのベースラインからの変化量の差が 0.6 で、その標準偏差が 0.5(エフェクトサイズ 1.2)のとき、両側有意水準 5%の下、検出力 80%を維持することができる。

## 10.2. 解析対象集団

主要エンドポイント及び副次エンドポイントの解析は、Full Analysis Set (FAS)を対象としたものを主解析とする。また、Per Protocol Set (PPS)を対象とした解析も実施し、解析結果の安定性を確認する。

FAS は、全てのランダム化(登録)された症例から、①確定診断により対象外疾患と判定されている症例、②明確に定義された客観的に判定可能な重要な選択・除外基準に抵触する症例)、③登録以降試験薬を一度も服用していない症例、④登録以降のデータが全くない症例、いずれかに該当する症例を除いた集団と定義する。

PPS は、研究計画書の主要変数に関する最低限の規定を満たす症例で、プロトコール治療を終了し、適格性基準やプロトコール治療、併用禁止薬等に関する重大な研究計画書違反が認められない症例と定義する。

## 10.3. 解析項目・方法

### 10.3.1. 主要エンドポイントの主たる解析方法

FAS を対象とし、プロトコール治療終了時点(投薬開始 5 日後)のベースラインからの HOMA-IR の変化量について、リスペリドン群と非投与群が等しいという帰無仮説に対し、Student の *t*-検定を行う。

### 10.3.2. 主要エンドポイントの主たる副次解析方法

FAS を対象とし、試験薬投薬終了時点(投薬開始 5 日後)のベースラインからの HOMA-IR の

変化量について、年齢、性別、腎機能等の対象者の背景因子による調整後に平均を比較する。

#### 10.3.3. 副次エンドポイントの解析方法

FAS を対象とし、プロトコール治療終了時点（投薬開始 5 日後）のベースラインからの血糖値、インスリン、HbA1c、C ペプチド、血圧、BMI、eGFR、尿中アルブミン・クレアチニン比、キラルアミノ酸、16S rRNA の各変化量について、リスペリドン群と非投与群が等しいという帰無仮説に対し、Student の *t*-検定を行う。

有害事象発現については、FAS を対象とし、プロトコール治療終了時点（投薬開始 5 日後）について、リスペリドン群と非投与群が等しいという帰無仮説に対し、カイ二乗検定を行う。

#### 10.3.4. 有意水準

有意水準は両側 5%とする

#### 10.3.5. 欠測データの取扱い等

欠落値は補填せず、欠落のまま解析を行う。

### 10.4. 中間解析

当該臨床研究では、被験者の安全性の観点から早期に研究を中断する必要性を判断するため、中間解析を実施する。中間解析の実施時期は、目標症例数の 1/2 の症例がプロトコール治療を終了した時点で行う。試験薬群のリスペリドンの有害事象の結果を見て、研究責任医師は当該臨床研究の中止を判断する。

### 10.5. 統計解析計画の変更に関する手順

「9. エンドポイントの定義」及び「10. 統計学的考察」に記載された内容を変更する場合は、「15.2. 研究計画書の改訂」に従い変更する。それ以外の統計解析計画の変更については、統計解析計画書に従って変更する。

## 11. 症例報告書の記入と提出

### 11.1. 種類と提出期限

症例報告書には以下の記載を含む

- 同意取得
- 研究対象者の背景
- 検査結果
- 試験薬投薬等の情報
- 有害事象の情報（重篤度、程度、転帰、終了日）
- 中止日、中止理由、中止の原因となった有害事象、中止後の経過及び追跡調査の結果
- 研究責任（分担）医師コメント

### 11.2. 記入方法

研究責任（分担）医師又は研究協力者は、CRF を以下の規定に従って記載する。なお CRF は、別途提供する「症例報告書の作成・修正に関する手引き」に従って記載する。

(1) CRF への記載は研究責任（分担）医師等が行う。

(2) CRF は登録が完了した症例について作成する。

(3) CRF の内容を変更又は修正する際、修正者、修正日、変更又は修正理由を記録する。

(4) 研究責任医師は、CRF が正確かつ完全に作成されていることを確認した上で、CRF に対して署

名を行う。
(5) 研究責任医師は、CRF の写しを保管する。

### 11.3. 送付方法

単施設での研究であり該当しない。

## 12. 原資料等の閲覧と品質管理及び品質保証

### 12.1. 直接閲覧の受入れと協力

研究責任医師及び実施医療機関は、当該臨床研究に関連するモニタリング並びに認定臨床研究審査委員会及び規制当局の調査に関し、原資料等の全ての臨床研究関連記録を直接閲覧に供し、また協力する。

### 12.2. モニタリング

研究責任医師はモニタリングに従事する者を指名する。モニタリング従事者は以下の観点から原資料を直接閲覧することによりモニタリングを実施する。

- 臨床研究の対象者の人権の保護、安全の確保が図られているか
- 臨床研究が最新の実施計画、研究計画書及び本規則を遵守して実施されているか
- 臨床研究の実施について臨床研究の対象者から文書により同意を得ているか
- 記録等が正確であるか

モニタリング従事者は当該臨床研究が研究計画書及び臨床研究法に従って実施されていることを確認するために、別途策定するモニタリング計画書に従って業務を実施する。

## 13. 倫理的事項

### 13.1. 遵守すべき諸規則

当該臨床研究に携わるすべての者は、人を対象とする全ての医学研究が準拠すべき「世界医師会ヘルシンキ宣言」及び臨床研究法と関連する省令・通知等の内容を熟読し理解した上で遵守し、臨床研究を施行する。

### 13.2. 研究開始に係る認定臨床研究審査委員会による審査と実施医療機関の管理者、厚生労働大臣への届出

当該臨床研究を実施する前に、研究責任医師は当該研究計画書及び実施計画（様式第一）、説明文書・同意文書、医薬品等の概要を記載した書類、当該臨床研究の実施に起因すると疑われる疾病等が発生したときの対応に関する手順書、利益相反管理基準及び利益相反管理計画、研究責任医師及び研究分担医師の氏名を記載した文書（統一書式1）、統計解析計画書を作成した場合にあっては、当該統計解析計画書、その他認定臨床研究審査委員会が求める書類を認定臨床研究審査委員会に提出し、意見を聴取する。研究責任医師は認定臨床研究審査委員会に提出した文書及び認定臨床研究審査委員会の意見を実施医療機関の管理者に提出して臨床研究実施の承認を得る。研究責任医師は認定臨床研究審査委員会の意見を反映した「実施計画」（様式第一）を厚生労働大臣に提出した後、臨床研究を開始する。

### 13.3. 説明文書・同意文書（様式）の作成と改訂

説明文書・同意文書は、研究責任医師が作成し、必要に応じてこれを改訂する。また、作成ある

いは改訂した説明文書・同意文書は、あらかじめ認定臨床研究審査委員会に提出する。また実施医療機関の管理者に認定臨床研究審査委員会の意見とともに報告し、承認を得る。

説明文書には、少なくとも以下に示す「臨床研究法施行規則」に定められた事項を掲載しなければならない。研究対象者を意図的に誘導するような記載をしてはならない。

- 実施する特定臨床研究の名称、当該特定臨床研究の実施について実施医療機関の管理者の承認を受けている旨及び厚生労働大臣に実施計画を提出している旨
- 実施医療機関の名称並びに研究責任医師の氏名及び職名
- 特定臨床研究の対象者として選定された理由
- 特定臨床研究の実施により予期される利益及び不利益
- 特定臨床研究への参加を拒否することは任意である旨
- 同意の撤回に関する事項
- 特定臨床研究への参加を拒否すること又は同意を撤回することにより不利益な取扱いを受けない旨
- 特定臨床研究に関する情報公開の方法
- 特定臨床研究の対象者又はその代諾者の求めに応じて、研究計画書その他の特定臨床研究の実施に関する資料を入手又は閲覧できる旨及びその入手又は閲覧の方法
- 特定臨床研究の対象者の個人情報の保護に関する事項
- 試料等の保管及び廃棄の方法
- 特定臨床研究に対する医薬品等製造販売業者等による研究資金等の提供その他の関与の状況、及び臨床研究に従事する者及び研究計画書に記載されている者に関する当該医薬品等製造販売業者等による寄附金、原稿執筆及び講演その他の業務に対する報酬の提供その他の関与の状況
- 苦情及び問合せへの対応に関する体制
- 特定臨床研究の実施に係る費用に関する事項
- 他の治療法の有無及び内容並びに他の治療法により予期される利益及び不利益との比較
- 特定臨床研究の実施による健康被害に対する補償及び医療の提供に関する事項
- 特定臨床研究の審査意見業務を行う認定臨床研究審査委員会における審査事項その他当該特定臨床研究に係る認定臨床研究審査委員会に関する事項
- その他特定臨床研究の実施に関し必要な事項

臨床研究開始後に研究責任医師が研究対象者の同意に関連する新たな知見を得て、説明文書・同意文書の変更が必要と判断した場合には、研究責任医師はそれを改訂し、認定臨床研究審査委員会に提出する。また実施医療機関の管理者に認定臨床研究審査委員会の意見とともに報告し、承認を得る。

なお、新たな知見とは、新たな安全性の情報又は当該疾患に関わる新治療法等の開発に関する情報等を指す。

### 13.4. インフォームド・コンセント

臨床研究についての説明を行い、十分に考える時間を与え、研究対象者が臨床研究の内容をよく理解したことを確認した上で、臨床研究への参加について依頼する。研究対象者本人が臨床研究参加に同意した場合、同意文書を用い、研究対象者本人による署名を得る。研究責

任医師又は研究分担医師は同意文書に、説明を行った医師名と説明日、説明を受け同意した研究対象者名、同意日の記載があることを確認する。*、**

同意文書は 2 部コピーし、1部は研究対象者本人に手渡し、1部は研究責任医師が保管する。原本は研究機関で定められた保管場所に保管する。

当該臨床研究において取得された個人情報は同意取得時点では特定されていない、将来の研究のために用いられる可能性があるため、その旨に関する同意を取得するとともに、将来の臨床研究実施の際には倫理審査委員会に研究計画書を提出して審査を受け、同意に関する再確認を行う。

* 説明文書には、研究対象者研究対象者となるべき者に権利を放棄させる旨又はそれを疑わせる記載並びに治験依頼者、自ら治験を実施する者、実施医療機関、治験責任医師等の責任を免除し若しくは軽減させる旨又はそれを疑わせる記載をしてはならない。

** 説明文書には、できる限り平易な表現を用いなければならない。

### 13.5. 試料・情報の二次利用

当該臨床研究で得られた研究対象者の試料・情報は、同意を受ける時点では特定されない将来の研究のために用いる可能性がある。その場合には、当該臨床研究で得られた試料をもとに研究を実施することに関して、研究内容に即した倫理審査委員会で審査を受けるとともに、別途研究対象者に説明した上で実施する。

## 14. 個人情報の取扱い

臨床研究実施に係る資料等を取り扱う際は、研究対象者の個人情報等は無関係の番号を付して管理し、研究対象者の秘密保護に十分配慮する。臨床研究の結果を公表する際は、研究対象者を特定できる情報を含まないようにする。また、臨床研究の目的以外に、研究で得られた研究対象者の試料等を使用しない。

## 15. 研究計画書の逸脱・変更・改訂

### 15.1. 研究計画書の逸脱又は変更

研究責任医師又は研究分担医師は認定臨床研究審査委員会の事前の審査に基づく病院長の承認を得る前に、研究計画書からの逸脱あるいは変更を行ってはならない。

研究責任医師又は研究分担医師は、緊急回避等のやむ得ない理由により、認定臨床研究審査委員会の事前の承認を得る前に、研究計画書からの逸脱あるいは変更を行うことができる。その際には、研究責任医師又は研究分担医師は、逸脱又は変更の内容及び理由ならびに研究計画書等の改訂が必要であればその案を速やかに、認定臨床研究審査委員会に提出し、認定臨床研究審査委員会及び病院長の承認を得るものとする。

研究責任医師又は研究分担医師は、研究計画書からの逸脱があった場合には逸脱事項をその理由とともにすべて記録しなければならない。

### 15.2. 研究計画書の改訂

研究計画書の改訂を行う場合、研究責任医師は、変更の妥当性及び臨床研究の評価への影響について、必要に応じて統計解析責任者等と協議した上で改訂を決定する。改訂の際には改

訂した研究計画書及び必要に応じて改訂した説明文書・同意文書を認定臨床研究審査委員会に提出し、意見を聴取する。また、改訂した研究計画書及び説明文書・同意文書と認定臨床研究審査委員会の意見を実施医療機関の管理者に報告して承認を得る。重大な変更の場合は、研究対象者保護の観点から症例登録の一時中止を検討する。利益相反管理基準又は利益相反管理計画を変更する場合も研究計画書を改訂する場合と同様に認定臨床研究審査委員会の意見聴取と実施医療機関の管理者の承認を得る。

改訂の承認を得た後、研究責任医師は、改訂内容を研究分担医師やデータセンター等に速やかに伝達するとともに研究計画書の変更を伴う場合は厚生労働大臣に実施計画事項変更届書（様式第二）を提出する。研究計画書の改訂に伴い、症例登録票や症例報告書の内容に影響がある場合は、速やかに当該箇所を改訂する。

尚、実施計画の軽微な変更（臨床研究従事者の変更を伴わない氏名の変更や、地名や番地の変更（移転ではなく地域名の変更））に該当する場合は、認定臨床研究審査委員会及び実施医療機関の管理者にその内容を通知するとともに、変更日から 10 日以内に厚生労働大臣に実施計画事項軽微変更届書（様式第三）を提出する。

# 16. 臨床研究の終了と早期中止

## 16.1. 臨床研究の終了

研究責任医師は研究計画書に記載した全ての評価項目に係る全てのデータの収集が終了してから原則 1 年以内に総括報告書及びその概要を作成し、認定臨床研究審査委員会に提出して意見を聴取する。また実施医療機関の管理者に総括報告書及びその概要、認定臨床研究審査委員会の意見を報告して承認を得る。研究責任医師は、「終了届」（別紙様式 1)を厚生労働大臣に提出し、認定臨床研究審査委員会が意見を述べた日から 1 ヵ月以内に jRCT に総括報告書の概要を公表する。jRCT による総括報告書の概要の発表に関し、当該臨床研究成果を論文等で公表する場合は、認定臨床研究審査委員会に論文投稿中の旨を報告した上で、当該論文等の公表後に jRCT による公表を行う。

## 16.2. 臨床研究の早期中止

以下のいずれかが認められた場合は当該臨床研究を中止する

中間解析により被験薬の有効性における優位性又は劣性が確認された。

中間解析により被験薬の優位性を証明できる確率が小さいことが判明した。

中間解析の結果、重篤な有害事象報告又は当該臨床研究以外の情報に基づき、被験薬の安全性に問題があると判定された。

当該臨床研究の安全性及び当局規制や研究計画書からの逸脱状況を鑑み、認定臨床研究審査委員会から中止の勧告又は指示があった、又は研究責任医師が中止すべきと判断した。

当該臨床研究以外の情報に基づき、被験薬の安全性に問題があると考えられたことから、認定臨床研究審査委員会から中止の勧告又は指示があった、又は研究責任医師が中止すべきと判断した。

その他、症例登録の遅れ、研究計画書からの逸脱の頻発などの理由により、臨床研究の完遂が困難と判断された。

研究責任医師は、以下の事項に該当する場合は臨床研究実施継続の可否を検討する。

被験薬の品質、安全性、有効性に関する重大な情報が得られたとき
研究対象者のリクルートが困難で予定症例を達成することが到底困難であると判断されたとき
予定症例数又は予定期間に達する前に、（中間解析等により）臨床研究の目的が達成されたとき
認定臨床研究審査委員会により、実施計画等の変更の指示があり、これを受入れることが困難と判断されたとき

研究責任医師は当該臨床研究を中止すべきと考えた場合、研究対象者に必要な処置を講じる。必要に応じて研究対象者の措置に伴う臨床研究終了時期やその方法について、認定臨床研究審査委員会の意見を聴取する。研究責任医師は、認定臨床研究審査委員会に「中止通知書」（統一書式 11）を、厚生労働大臣に「特定臨床研究中止届書」（様式第四）をそれぞれ提出する。なお「特定臨床研究中止届書」を厚生労働大臣に提出後も臨床研究が終了するまでは定期報告を継続的に提出し、当該臨床研究の進捗状況に関する事項の変更に該当する場合には、実施計画の変更の届出を行う。

# 17. 実施医療機関の管理者等への報告

## 17.1. 実施医療機関の管理者への報告事項

研究責任医師は、以下の事象について自らが所属する実施医療機関の管理者へ報告しなければならない。

- 不適合（研究責任医師から実施医療機関の管理者に報告されない懸念があるときは研究分担医師から実施医療機関の管理者に報告）
- 医薬品等製造販売業者等による研究資金等の提供その他の関与
- 認定臨床研究審査委員会の意見
- 総括報告書の概要
- 総括報告書の概要を公表したこと
- 実施計画を提出したこと
- 臨床研究実施によるものと思われる重篤な有害事象の発現及びその後
- 不具合
- 疾病等報告
- 定期報告

研究責任医師は、以下の事象について自らが所属する実施医療機関の管理者の報告に加え、承認を得なければならない。

- 臨床研究実施の可否
- 特定の条件を満たした 16 歳以上の未成年から同意を取得（代諾者なし）

なお、研究分担医師等は不適合を知った場合は速やかに研究責任医師に報告する。

## 17.2. 認定臨床研究審査委員会への報告事項

研究責任医師は、以下の事象について認定臨床研究審査委員会意見を求める又は報告しなければならない。

- 研究開始
- 臨床研究実施によるものと思われる重篤な有害事象の発現及びその後
- 疾病等報告

- 定期報告
- 実施計画の変更
- 研究計画書、利益相反管理基準、利益相反管理計画の変更
- 臨床研究の中止（必要に応じて）
- 重大な不適合
- 総括報告書の概要、公表（jRCT 登録）
- 特定の条件を満たした 16 歳以上の未成年から同意を取得（代諾者なし）

研究責任医師は、以下の事象について認定臨床研究審査委員会に通知しなければならない。

- 実施計画を厚労大臣へ提出したこと

### 17.3. 厚生労働大臣への報告事項

研究責任医師は、以下の事象を厚生労働大臣（地方厚生労働局長）に報告しなければならない。

- 実施計画（研究開始）
- 実施計画の変更
- 実施計画の軽微変更
- 臨床研究の中止
- 臨床研究の終了
- 定期報告

研究責任医師は、以下の事象を厚生労働大臣（独立行政法人医薬品医療機器総合機構　理事長）に報告しなければならない。

- 疾病等報告（未承認薬・適用外使用を含む特定臨床研究で臨床研究実施に起因すると思われる予測されない重篤な有害事象の情報）

## 18. 臨床研究に関する記録の取り扱い

研究責任医師は、臨床研究等の実施に係わる文書（申請書類の控え、認定臨床研究審査委員会からの通知文書、実施医療機関の管理者からの通知文書、各種申請書・報告書の控、研究対象者識別コードリスト、（スクリーニング名簿）、同意に係る文書、登録に係る文書、疾病等の対応に係る文書、症例報告書等の控え、臨床研究実施に係る契約書、被験薬、被験医療機器、被験再生医療製品の入手及び使用、廃棄等の処分に関する記録、各種手順書、モニタリング報告書、その他データの信頼性を保証するのに必要な書類又は記録など）を jRCT に総括報告書の概要が公表された日又は当該臨床研究の結果の最終公表（論文等）がなされた日のいずれか遅い時期から5年を経過した日までの期間、適切に保存し、その後は個人情報に注意して廃棄する。

診療録については、当院の規定に基づき、保管・廃棄を行う。

- 研究計画書、実施計画、特定臨床研究の対象者に対する説明及びその同意に係る文書、総括報告書その他のこの省令の規定により研究責任医師が作成した文書又はその写し
- 認定臨床研究審査委員会から受け取った審査意見業務に係る文書
- モニタリングに関する文書
- 原資料等
- 特定臨床研究の実施に係る契約書

- 特定臨床研究に用いる医薬品等の概要を記載した文書及び医薬品等の入手と処分の記録

# 19. 臨床研究の実施に係る金銭の支払及び補償

## 19.1. 臨床研究に関する費用

当該臨床研究における試験薬リスペリドンに関しては当該臨床研究グループ及び金沢大学附属病院の 2021 年度臨床研究等に係る公募研究助成からの助成から負担する。

研究対象者に対する謝礼（一人 1 visit につき 5000 円、Quo カードで支払い、合計 20000 円まで）に関しては当該臨床研究グループ及び金沢大学附属病院の 2021 年度臨床研究等に係る公募研究助成からの助成から負担し支払う。

## 19.2. 健康被害に対する補償

当該臨床研究に起因した健康被害が発生した場合には適切な治療を行う。医薬品副作用被害救済制度でいう死亡・後遺障害 1 級及び 2 級に該当する健康被害が発生した場合は、当該臨床研究グループが金沢大学附属病院の 2021 年度臨床研究等に係る公募研究助成により加入している「臨床研究に係る補償責任保険」にて対応する。

# 20. 研究資金及び利益相反管理

## 20.1. 利益相反管理

研究責任医師、研究分担医師、統計解析責任者は、本臨床研究に対する利益相反はない。

## 20.2. 研究資金源

当該臨床研究は、研究グループおよび金沢大学附属病院の 2021 年度臨床研究等に係る公募研究助成からの助成を受けて実施する。

# 21. 研究成果の帰属と結果の公表

当該臨床研究は臨床研究開始前に、jRCT に研究内容を登録し、公表する。また、総括報告書の概要に関し、特定臨床研究審査委員会の意見を聴取した日から 1 ヵ月以内に jRCT に登録し、公表する。jRCT による総括報告書の概要の公表に関し、当該臨床研究成果を論文等で公表する場合は、認定臨床研究審査委員会に論文投稿中の旨を報告した上で、当該論文等の公表後に jRCT による公表を行う。

当該臨床研究の成果は、研究グループに帰属するものとする。研究責任医師及び統計解析責任者が協議して著者を選出し、学会又は論文にて報告する。

# 22. 研究組織

## 22.1. 研究責任医師

金沢大学附属病院　検査部　特任助教　大島　恵

住所〒920-8641　石川県金沢市宝町 13-1

電話：076-265-2499

## 22.2. 研究事務局（調整管理実務担当者）

金沢大学附属病院　腎臓内科学　事務
住所　〒920-8641　石川県金沢市宝町 13-1
電話：076-265-2499　FAX：076-234-4273
E-mail：lab-med@med.kanazawa-u.ac.jp

## 22.3. データマネジメント責任者

金沢大学附属病院　先端医療開発センター　データセンター部門　高原　志津子
住所〒920-8641　石川県金沢市宝町 13-1
電話：076-265-2873

## 22.4. 統計解析責任者

金沢大学附属病院　先端医療開発センター　生物統計部門　遠山　直志
住所　〒920-8641　石川県金沢市宝町 13-1
電話：076-265-2090

## 22.5. モニタリング責任者

金沢大学附属病院　先端医療開発センター　モニタリング・監査部門　杉本　修治
住所　〒920-8641　石川県金沢市宝町 13-1
電話：076-265-2878

## 22.6. 被験薬管理者

金沢大学附属病院　先端医療開発センター　臨床研究推進部門　長瀬　克彦
住所〒920-8641　石川県金沢市宝町 13-1
電話：076-265-2049

## 22.7. 症例登録センター

金沢大学附属病院　先端医療開発センター　データセンター部門
住所〒920-8641　石川県金沢市宝町 13-1
電話：076-265-2090 FAX：076-234-4346（受付日時　月～金の 9-17 時（祝日を除く））
責任者：　高原　志津子
委託業務内容：症例登録票の受領と内容確認、登録番号の付与と内容確認票の発行、登録情報の管理

## 22.8. 検体検査測定センター

BML 株式会社金沢営業所
住所〒920-8202　石川県金沢市西都 1-52
電話：076-266-0600
委託検査内容：血液検査（白血球数、赤血球数、ヘモグロビン、ヘマトクリット、血小板数、TP、Alb、AST、ALT、BUN、Cre、UA、Na、K、Ca、P、Mg、空腹時血糖、HbA1c、T-Cho、HDL-Cho、TG、インスリン、C ペプチド）、尿検査（糖、潜血、Alb、Cre）

KAGAMI 株式会社
住所〒567-0085　大阪府茨木市彩都あさぎ 7 丁目 7 番 18 号
彩都バイオヒルズセンター　308 号室
電話：072-646-7059

担当者: 三田 真史
委託検査内容:キラルアミノ酸

タカラバイオ株式会社
住所〒525-0058 滋賀県草津市野路東七丁目 4 番 38 号
電話:077-565-6920
委託検査内容:16S rRNA 菌叢解析

## 23. 苦情及び相談窓口

研究対象者等及びその関係者からの苦情及び相談等への対応窓口として、研究事務局が対応する。

住所 〒920-8641 石川県金沢市宝町 13-1 担当 大島 恵
電話:076-265-2499
FAX:076-234-4273

## 24. 文献

1) Koppe L, Mafra D, Fouque D. 2015. Probiotics and chronic kidney disease. Kidney Int. 88(5):958-66.
2) Nakade Y, Iwata Y, Furuichi K, et al. 2018. Gut microbiota-derived D-serine protects against acute kidney injury. JCI Insight. 18;3(20). pii: 97957.
3) Kimura T, Hamase K, Miyoshi Y, et al. 2016. Chiral amino acid metabolomics for novel biomarker screening in the prognosis of chronic kidney disease. Sci Rep. 18;6:26137.

## 25. 付録

1. PMDA 資料(リスパダール)
2. 添付文書(リスパダール)

# Effects of risperidone on glucose metabolism and gut microbiota in healthy adults: an open-label, pilot, randomized controlled trial

## Research Protocol

[Abbreviated title: Risperidone Healthy Subject Pilot Study]

**Principal Investigator:** Megumi Oshima, Assistant Professor Department of Laboratory Medicine, Kanazawa University Hospital

**Research Secretariat:** Department of Nephrology, Kanazawa University Graduate School Address: 13-1 Takara-machi, Kanazawa, Ishikawa 920-8641, Japan Phone: 076-265-2499 FAX: 076-234-4273 E-mail: lab-med@med.kanazawa-u.ac.jp

Protocol Draft Ver. 0.1: December 1, 2021 Protocol Draft Ver. 0.2: February 8, 2022 Clinical Research Review Committee Approved Version Ver. 1.0: March 9, 2022 Ver. 1.1: November 11, 2022 Ver. 1.2: January 18, 2023

## Table of Contents

[TOC omitted for brevity]

# 0. Research Overview

## 0.1. Schema

[Schema would be here in the original document]

## 0.2. Summary of Clinical Research Design

Prospective, interventional study, specific clinical research, no-treatment control, randomized (minimization method), non-blinded, single-center

## 0.3. Type of Clinical Research

Specific clinical research (no funding from companies, off-label use)

# 1. Objective

This research is a non-blinded randomized controlled trial with a no-treatment control group to evaluate the effects of risperidone administration on glucose metabolism indicators and intestinal environment in healthy adults.

The primary endpoint is the change in insulin resistance, and the secondary endpoint is the change in intestinal bacterial flora.

# 2. Background

## 2.1. Status of Target Disease in Japan and Overseas (Including Epidemiological Data)

The number of chronic kidney disease (CKD) patients in Japan is estimated to be approximately 14 million. CKD not only leads to end-stage renal disease as it progresses but also leads to the development and death from cardiovascular disease, making its control an important issue. In particular, diabetic kidney disease, which has diabetes as its background, remains the number one cause of dialysis initiation (41.6%) (Current Status of Chronic Dialysis Therapy in Japan, 2019) and is the most important disease from the perspective of cardiovascular disease development and life prognosis.

## 2.2. History and Content of Standard Treatments Implemented to Date

For the treatment of diabetic kidney disease, the importance of strict blood glucose or blood pressure management has been demonstrated by DCCT/EDIC, Kumamoto Study, UKPDS, etc., conducted in the 1990s. In the 2000s, large-scale clinical trials using renin-angiotensin system (RAS) inhibitors as antihypertensive drugs revealed the renoprotective effect of RAS inhibitors, especially their effect on reducing albuminuria. As a result, RAS inhibitors are recommended as the first-choice antihypertensive drugs for diabetic patients in the guidelines for CKD and hypertension. In the 2010s, the renoprotective effects of diabetes drugs such as DPP-4 (dipeptidyl peptidase-4) inhibitors, GLP-1 (glucagon-like peptide-1) receptor agonists, and SGLT2 (sodium-glucose cotransporter 2) inhibitors have been verified by various large-scale clinical trials, and the treatment policy for diabetic kidney disease is changing.

## 2.3. Current Standard Treatment and Treatment Outcomes

Based on the above background, the "Evidence-based CKD Treatment Guidelines 2018" recommends intensive treatment, including appropriate management of blood glucose and blood pressure, to suppress the progression of CKD with diabetes as a background. On the other hand, in the CREDENCE trial, a large-scale clinical trial targeting CKD with type 2 diabetes, it is estimated that one in 49 patients per year will reach end-stage renal disease even if they take SGLT2 inhibitors in addition to RAS inhibitors, and it is important to reduce the residual risk.

## 2.4. Current Standard Treatment Issues and Unknowns Leading to the Need for This Clinical Research

Despite current treatments, how to reduce the number of patients who reach end-stage renal disease with diabetes as a background remains an important issue from the perspective of appropriate medical costs and life prognosis.

In recent years, it has been known that the intestinal bacterial flora changes in kidney disease and affects renal function, and amino acids have been attracting attention as one of its metabolites (1). With recent advances in analytical technology, it has become possible to separate amino acids into optical isomers, L-form and D-form, for measurement. It has been revealed that among D-amino acids, blood D-serine increases in mice with acute kidney injury and correlates with renal function, and that kidney damage is reduced by D-serine administration (2). D-amino acids are expected to be utilized for biomarker and therapeutic drug development for kidney diseases.

Risperidone is a representative drug with D-amino acid oxidase (DAO) inhibitory action and is already approved as a therapeutic drug for schizophrenia, a mental disorder that causes a decrease in the function of NMDAR (N-methyl-D-aspartate receptor: NMDAR), which is a receptor for D-amino acids. We have also observed improvement in insulin resistance by risperidone administration in mice with diabetic nephropathy (manuscript in preparation). Therefore, it is suggested that the D-amino acid oxidase inhibitory action of risperidone leads to renoprotection through improvement of glucose tolerance, but verification in humans has not been conducted, and the safety and effects on intestinal bacterial flora in patients with renal dysfunction have not been elucidated.

Based on the above background, this study evaluates the effects of risperidone administration on glucose metabolism indicators and intestinal environment in healthy adults with insulin resistance. As an indicator of insulin resistance, HOMA-IR > 1.6 is used because it has been reported that HOMA-IR correlates well with insulin resistance determined by the glucose clamp method when fasting blood glucose is 140 mg/dL or less.

If this study reveals the effects of risperidone on glucose tolerance and intestinal environment, as well as its safety, we plan to design a trial of risperidone administration for patients with diabetic kidney disease in the next stage.

**[Rationale for Dosage and Administration]**

In a Phase I pharmacokinetic study (RIS-P01-101 study) conducted to compare the pharmacokinetics, safety, and tolerability of risperidone tablets 1 mg after single (day 1) and repeated (days 5-11) administration in healthy Japanese and Caucasian adults (24 subjects each), the incidence of adverse events in the safety analysis population was 100% (24/24 subjects) in both groups (Appendix 1). Frequently reported adverse events in the Japanese group were somnolence (24/24 subjects, 100%), orthostatic hypotension (9/24 subjects, 38%), and dizziness (8/24 subjects, 33%). No deaths or other serious adverse events were observed in either group.

Since improvement in insulin resistance was observed with repeated administration in animal experiments, this study will conduct repeated administration after considering safety, as a single administration would not achieve the goal. Regarding the dose, 0.5 mg/day, which is the lowest dose, will be used considering safety. Schizophrenia, for which risperidone is covered by insurance, is not the target disease of this study.

# 2.5. Benefits and Disadvantages of Protocol Treatment

## 2.5.1. Burden and Predicted Risks for Research Subjects

Subjects will bear the burden of blood collection and the risk of adverse events from the test drug. There will be no economic burden.

## 2.5.2. Expected Benefits for Research Subjects

Research subjects will be paid a stipend. They may contribute to the advancement of medicine through research results.

## 2.5.3. Overall Evaluation and Measures to Minimize Burden and Risks

To minimize the burden on subjects, blood collection will be limited to the minimum amount and frequency. Because the test drug risperidone may cause drowsiness, decreased attention, concentration, and reflex motor abilities, it will be administered before bedtime, and subjects will be instructed not to engage in operating dangerous machinery such as driving a car while taking the medication and to refrain from drinking alcohol. In the event of health damage caused by this clinical research, appropriate treatment will be provided, and in the case of health damage corresponding to death or disability grades 1 and 2 as defined by the Pharmaceutical Affairs Relief System, it will be covered by the "Clinical Research Compensation Liability Insurance" that the clinical research group has joined through the 2021 Clinical Research Funding of Kanazawa University Hospital.

# 2.6. Research Design

This clinical research is a prospective non-blinded randomized controlled trial on the effects of risperidone on glucose metabolism indicators and intestinal environment in healthy adults. After registration, subjects will be randomly assigned to the test drug administration (risperidone) group or the non-administration group in a 1:1 ratio using the minimization method with age and gender as allocation adjustment factors. After allocation, the test drug risperidone will be taken for 4 days (protocol treatment period), and the change in HOMA-IR value will be evaluated. The protocol treatment period will be conducted under non-blinded conditions. After completion, there will be a 5-day post-observation period to confirm the presence or absence of adverse events.

The research will be conducted in accordance with the "World Medical Association Declaration of Helsinki" and the Clinical Trials Act, with consideration for the safety of research subjects and minimizing bias.

## 2.7. Overview of the Investigational Drug

- Investigational drug name: Risperidone (Trade name: Risperdal®)
- Manufacturer (or distributor): Janssen Pharmaceutical K.K.
- Pharmacological classification: Atypical antipsychotic (serotonin-dopamine antagonist)
- Mechanism of action: Mainly based on dopamine D2 receptor antagonism and serotonin 5-HT2 receptor antagonism, it is thought to work through regulation of the central nervous system
- Indications: Schizophrenia, irritability associated with autism spectrum disorder in childhood
- Route of administration, dosage and administration, method of use: For schizophrenia, oral administration usually starts with risperidone 1 mg once daily twice a day for adults and is gradually increased. The maintenance dose is usually 2-6 mg daily, in principle divided into two oral doses daily.
- Contraindications: Patients in a comatose state, patients under the strong influence of central nervous system depressants such as barbiturate derivatives, patients receiving adrenaline, patients with a history of hypersensitivity to the components of this drug and paliperidone
- Main clinical performance: In clinical trials conducted in Japan, including double-blind comparative trials, involving a total of 727 patients with schizophrenia and schizoaffective disorder, the improvement rate of moderate or greater improvement among 722 cases subject to efficacy evaluation was 51.5% (372/722 cases). In addition, the usefulness of this drug for schizophrenia has been confirmed by double-blind comparative trials. Adverse reactions were observed in 420 of 723 cases (58.1%) in the safety evaluation population. The main ones were akathisia in 126 cases (17.4%), tremor in 95 cases (13.1%), irritability in 92 cases (12.7%), insomnia in 87 cases (12.0%), muscle rigidity in 85 cases (11.8%), and hypersalivation in 81 cases (11.2%).
- Adverse reactions/malfunctions:
    1. Serious adverse reactions: Malignant syndrome, tardive dyskinesia, paralytic ileus, syndrome of inappropriate antidiuretic hormone secretion (SIADH), liver dysfunction, jaundice, rhabdomyolysis, arrhythmia, cerebrovascular disorder, hyperglycemia, diabetic ketoacidosis, diabetic coma, hypoglycemia, agranulocytosis, leukopenia, pulmonary embolism, deep vein thrombosis, priapism
    2. Other adverse reactions: Refer to the package insert (Appendix 2)
- Interactions: Refer to the package insert
- Precautions for use: Refer to the package insert

# 3. Eligibility Criteria

## 3.1. Selection Criteria

The following subjects who meet all of the following will be eligible:

1. Men and women aged 20 to 65 years
2. Those without mental disorders, neurological disorders, or a history of them
3. Fasting blood glucose less than 140 mg/dL
4. HOMA-IR (homeostasis model assessment-insulin resistance) 1.6 or higher
5. Estimated GFR 60 ml/min/1.73 $m^2$ or higher
6. AST and ALT less than 30 IU/L
7. Those without a history of arrhythmia and congenital QT prolongation syndrome
8. Those without a history of active cancer (disease-free period within 5 years)
9. Those who have bowel movements at least once every 4 days (regardless of whether or not laxatives are used)
10. Those who have given written consent of their own free will after sufficient informed consent for participation in this research

## 3.2. Exclusion Criteria

1. Those with diseases under medical treatment
2. Those with a history of hypersensitivity to the components of risperidone and paliperidone
3. Those who are pregnant or breastfeeding
4. Those who participated in other clinical research within 3 months before the start of the investigational drug administration
5. Those whom the principal investigator or sub-investigator judges to be inappropriate as research subjects
6. Faculty and staff of the same or related department as the principal investigator

[Setting rationale] 1)5)6) For the influence on efficacy evaluation and consideration for safety, 4) For the influence on efficacy evaluation, 2)3)4) For consideration for safety

# 4. Registration and Allocation

## 4.1. Procedure for Case Registration

The principal investigator or sub-investigator of this clinical research will register cases according to the following procedure:

Recruitment will be conducted through the Kanazawa University Acanthus Portal and other means for Kanazawa University faculty and staff, seeking voluntary research participants.

The research will be explained to participants who wish to participate, excluding faculty and staff of the same or related department as the principal investigator. They will be given sufficient

time to consider, and after confirming that the research subject understands the content of the clinical research well, they will be asked to participate in the clinical research, and written consent will be obtained.

1. After obtaining consent, the principal investigator or sub-investigator will conduct screening tests and confirm that the target patient meets all eligibility criteria and does not fall under any exclusion criteria. The principal investigator will record necessary items (date of consent, research subject identification code, research subject name, medical record number, etc.) in the research subject list kept by the principal investigator, matching the research subject with the research subject identification code.
2. The principal investigator or sub-investigator will create a case registration form using the research subject identification code and fax it to the case registration center.
3. A case registration confirmation form with the registration number and allocation group will be sent from the case registration center to the reply fax listed on the case registration form. The principal investigator or sub-investigator will begin the clinical research after receiving this case registration confirmation form.
4. If consent withdrawal, discontinuation, dropout, etc. occurs, it will be promptly reported to the principal investigator.
5. If registration errors, duplicate registrations, etc. occur, they will be promptly reported to the registration center.

## 4.2. Allocation Method and Allocation Adjustment Factors

The case registration center will confirm eligibility based on the contents of the case registration form and perform allocation. For allocation, dynamic allocation will be performed using the minimization method with 1) age and 2) gender as allocation adjustment factors so that the ratio will be 1:1 in principle.

# 5. Clinical Research Plan

## 5.1. Protocol Treatment

## 5.1.1. Drug Group/Surgery Group/Examination Group, etc.

- Risperidone group (12 cases)
- Non-administration group (12 cases)

## 5.1.2. Procedure and Chronological Schedule for Drug Administration/Surgery/Examination, etc.

## 5.1.2.1. Drug Administration Site/Surgery Site/Examination Site, etc.

The risperidone group will orally administer risperidone.

## 5.1.2.2. Timing and Period of Drug Administration/Surgery/Examination, etc.

The medication period for the risperidone group is 4 days.

## 5.1.2.3. Dosage and Administration, Frequency, Duration, etc.

The risperidone group will start taking risperidone 0.5 mg (Risperdal OD 0.5 mg) once daily, before bedtime, for 4 days within 30 days after registration. The risperidone group will bring the remaining medication at the end of medication and it will be collected. Considering safety, the minimum dose of 0.5 mg/day will be used.

All subjects will visit on days 1, 5, and 9 (or at discontinuation). From one week before medication until the end of post-observation, the intake of foods containing probiotics that affect intestinal bacteria (yogurt, lactic acid beverages, natto, miso, rice bran pickles, kimchi, and other fermented foods) will be restricted. During medication, to reduce the risk of adverse events, subjects will be instructed not to engage in operating dangerous machinery such as driving a car and not to drink alcohol. No hospitalization will be conducted.

The principal investigator or sub-investigator will also confirm the compliance with the agreed matters through an interview with the subjects.

# 5.2. Dose/Schedule Change Criteria

- If a disease requiring treatment is recognized after registration and allocation, medication will be postponed until the treatment of the disease is completed.
- Since the test drug risperidone uses the minimum dose, no dose reduction will be performed, and if adverse events that do not allow continuation of medication are recognized, it will be discontinued.

# 5.3. Concomitant Treatment/Supportive Therapy

Prohibited concomitant treatment: Drugs with the same indication or mechanism of action (antipsychotics), drugs with the same side effects on the nervous system (hypnotics, anxiolytics), drugs listed as contraindicated in the package insert (adrenaline) are prohibited. Concomitant use of blood glucose-lowering drugs (including SGLT2 inhibitors) that affect efficacy during the test period is prohibited. Concomitant use of antibiotics and lactic acid bacteria preparations that affect the intestinal bacterial flora during the test period is prohibited.

Restricted concomitant treatment: From one week before medication until the end of post-observation, the intake of foods containing probiotics that affect intestinal bacteria (yogurt, lactic acid beverages, natto, miso, rice bran pickles, kimchi, and other fermented foods) will be restricted. Caution should be exercised when combining drugs listed as requiring caution in the package insert (central nervous system depressants, dopamine agonists, antihypertensive drugs, alcohol, drugs that inhibit CYP2D6 (paroxetine, etc.), drugs that induce or inhibit CYP3A4 (rifampicin, itraconazole, etc.), drugs known to cause QT prolongation) during the test period.

## 5.4. Discontinuation of Protocol Treatment

If the principal investigator or sub-investigator determines that the continuation of clinical research is impossible for the following reasons, the administration of the investigational drug will be discontinued. The date of discontinuation, reason, and course will be recorded in the medical record and case report form (CRF), and necessary tests will be conducted at the time of discontinuation or dropout, and efficacy and safety will be evaluated. If discontinued due to the occurrence of an adverse event, follow-up will be continued as much as possible until recovery to the current status.

When there is a request to withdraw from clinical research participation or withdrawal of consent from the research subject

When it is found that eligibility is not met after registration

When continuation of clinical research is difficult due to complications

When continuation of clinical research is difficult due to adverse events

When pregnancy is discovered

When the research subject does not visit due to relocation, etc.

When the entire clinical research has been discontinued

When the principal investigator determines that it is appropriate to discontinue clinical research for other reasons

## 5.5. Post-treatment

Post-treatment is not specified.

## 5.6. Management Procedures for Investigational Drugs

The principal investigator will appoint an investigational drug manager after confirming the purchase status of the investigational drug (number purchased, date of purchase, etc.). The investigational drug manager will manage the prescription status (identification number of the

research subject prescribed, amount prescribed, date of prescription, etc.), disposal, or handling of unused products. The investigational drug will be stored under the conditions described in the package insert and will not be used for purposes other than this research.

## 5.7. Storage of Samples/Information and Use of Samples/Information from Other Institutions

The principal investigator will instruct the sub-investigators to store samples appropriately according to the specified storage method and will perform necessary management to prevent leakage, mixing, theft, loss, etc. of samples. Collected blood will be stored frozen in the Department of Nephrology until 10 years after the end of the clinical research. When discarding, it will be done with attention to personal information after anonymization.

This research will not provide materials or information to other institutions. There is also no possibility of obtaining important findings related to the health of research subjects or genetic characteristics that may be inherited by their descendants.

# 6. Observation/Examination/Reporting Items and Schedule

## 6.1. Observation/Examination Items and Treatment Information to be Reported

- At registration Research subject information: Date of birth, gender, previous medical history, concurrent diseases Blood tests (in principle, fasting or 6 hours after meal): Fasting blood glucose, insulin, creatinine (Cre), AST, ALT Physiological test: Electrocardiogram *Regarding Cre, AST, ALT, and electrocardiogram, if there is a history of examination within the past year, the examination results brought by the research subject can be confirmed by the principal investigator or sub-investigator and can be used as a substitute.
- Before medication administration Research subject information: Identification code, height/weight, blood pressure, pulse, lifestyle (current smoking/drinking status) Blood tests (in principle, fasting or after at least 6 hours have passed since a meal): White blood cell count, red blood cell count, hemoglobin, hematocrit, platelet count, TP, Alb, AST, ALT, BUN, Cre, UA, Na, K, Ca, P, Mg, fasting blood glucose, HbA1c, T-Cho, HDL-Cho, TG, insulin, C-peptide Early morning urine test: Qualitative (glucose, occult blood), quantitative (Alb, Cre) Special test: Chiral amino acids (blood, urine, feces, saliva), intestinal bacteria 16S rRNA flora analysis (feces, saliva) *Stool collection will be done after registration and before the start of medication.
- At the end of medication/discontinuation Research subject information: Weight, blood pressure, pulse Blood tests (in principle, fasting or 6 hours after meal): White blood cell count, red blood cell count, hemoglobin, hematocrit, platelet count, TP, Alb, AST, ALT,

BUN, Cre, UA, Na, K, Ca, P, Mg, fasting blood glucose, HbA1c, T-Cho, HDL-Cho, TG, insulin, C-peptide Early morning urine test: Qualitative (glucose, occult blood), quantitative (Alb, Cre) Special test: Chiral amino acids (blood, urine, feces, saliva), intestinal bacteria 16S rRNA flora analysis (feces, saliva) Physiological test: Electrocardiogram Presence of adverse events/medication status *Stool collection will be done after the end of medication (or discontinuation) and before post-observation.

- At post-observation Research subject information: Weight, blood pressure, pulse Blood tests (in principle, fasting or 6 hours after meal): Cre, fasting blood glucose, HbA1c, insulin, C-peptide Early morning urine test: Quantitative (Alb, Cre) Presence of adverse events *Medical interview, examination, and blood collection will be conducted at the laboratory of the Department of Nephrology, Kanazawa University Graduate School, or the Kanazawa University Health Service Center. Urine will be collected from the early morning urine of the same day.

## 6.2. Observation/Examination/Reporting Schedule

[Table showing visit schedule with timing and permitted deviations]

*Medical interview, examination, blood collection, urine collection, stool collection, saliva collection, and electrocardiogram examination will be conducted at the laboratory of the Department of Nephrology, Kanazawa University Graduate School, or the Kanazawa University Health Service Center.

# 7. Target Sample Size and Clinical Research Period

## 7.1. Target Sample Size

Number of participating facilities: 1 facility, target sample size: 24 cases (risperidone group: 12 cases, non-administration group: 12 cases)

## 7.2. Clinical Research Period

Registration period: From the first publication date on jRCT to March 31, 2024 Observation period: From the first publication date on jRCT to September 30, 2024 (period for collecting data on all evaluation items described as matters related to the content of clinical research from the time the first case is registered) Research implementation period: From the first publication date on jRCT to March 31, 2025 (period from registering the implementation plan in the Japan Registry of Clinical Trials (jRCT) to registering the summary of the comprehensive report in jRCT)

# 8. Evaluation and Reporting of Adverse Events (Diseases, etc.)

## 8.1. Definition of Adverse Events (Diseases, etc.)

An adverse event is any unfavorable or unintended disease or its sign (including abnormal laboratory values) that occurs in a research subject, regardless of whether there is a causal relationship with the research conducted.

A "serious adverse event" refers to any of the following among adverse events:

1. Those resulting in death
2. Those that are life-threatening
3. Those requiring hospitalization for treatment or extension of hospitalization period
4. Those resulting in permanent or significant disability/dysfunction
5. Those causing congenital abnormalities in offspring

An "unexpected serious adverse event" refers to a serious adverse event that is not described in the research protocol, informed consent explanation document, etc., or that does not match the described content in nature or severity even if it is described.

An adverse drug reaction (ADR) refers to an adverse event for which a causal relationship with the use of the drug in question cannot be ruled out (those determined to have a causal relationship with the use of the drug that "cannot be ruled out". Those with an unknown causal relationship are also included).

An unexpected adverse drug reaction refers to an adverse drug reaction that is not described in the investigational drug brochure or package insert, or that does not match the described content in nature or severity even if it is described.

## 8.2. Evaluation and Reporting of Adverse Events

Evaluation of adverse events will be judged according to MedDRA/J (Medical Dictionary for Regulatory Activities/J: Japanese version of ICH International Medical Term Dictionary).

If the principal investigator or sub-investigator recognizes the occurrence of an adverse event, they will take appropriate measures based on the Ministry of Health, Labour and Welfare's "Manuals for Handling Serious Adverse Drug Reactions by Disease," report it via a case report form, and observe it until the end of the clinical research period regardless of whether there is a causal relationship, and follow up even after that until recovery as much as possible. All adverse events that occur in the research subject up to 30 days after the end or discontinuation of the protocol treatment will be observed regardless of whether there is a causal relationship. However, this does not apply if symptoms associated with the research subject's complications

have become chronic or if continuous observation is difficult due to transfer to another hospital, etc.

When a serious adverse event occurs, the principal investigator will promptly report it to the head of the research institution according to the institution's procedures. The principal investigator will report the progress of the research and the occurrence of adverse events associated with the implementation of the research to the head of the research institution once a year.

# 8.3. Expected Adverse Events

The following adverse events are expected from the package insert:

- Serious adverse events (serious side effects in the package insert): Malignant syndrome (frequency unknown), tardive dyskinesia (0.55%), paralytic ileus (frequency unknown), syndrome of inappropriate antidiuretic hormone secretion (SIADH) (frequency unknown), liver dysfunction/jaundice (frequency unknown), rhabdomyolysis (frequency unknown), arrhythmia (frequency unknown), cerebrovascular disorder (frequency unknown), hyperglycemia/diabetic ketoacidosis/diabetic coma (frequency unknown), hypoglycemia (frequency unknown), agranulocytosis/leukopenia (frequency unknown), pulmonary embolism/deep vein thrombosis (frequency unknown), priapism (frequency unknown)
- Other side effects in the package insert (incidence 5% or more): Anorexia, insomnia/anxiety, akathisia/tremor/dysarthria/somnolence/dizziness/staggering, gastrointestinal disorders, muscle rigidity, menstrual disorders, irritability/fatigue/dry mouth

# 8.4. Reporting and Response to Serious Adverse Events

### 8.4.1. Reporting of Serious Adverse Events Occurring in the Implementation of This Clinical Research

1. If a serious adverse event occurs, the sub-investigator will take appropriate measures and immediately report it to the principal investigator, regardless of the causal relationship with the investigational drug.
2. The principal investigator will promptly determine whether there is a causal relationship between the serious adverse event and the implementation of this clinical research and whether it is a known event, and will respond in accordance with the "Procedures for Reporting Diseases and Malfunctions in Clinical Research under the Clinical Trials Act at Kanazawa University." That is, if a serious adverse event occurs, within 72 hours of knowing it, the "Pharmaceutical Disease Report (First Report)" (Unified Form 8) will be submitted using the Kanazawa University Clinical Research Electronic Application System (and the "Disease Report (Pharmaceutical)" (Separate Form 2-1) will be attached if it is a serious adverse event that needs to be reported to the Minister of Health, Labour and Welfare) and report to the hospital director (the content submitted via the Kanazawa

University Clinical Research Electronic Application System is also submitted to the secretariat of the Certified Clinical Research Review Committee for events suspected to have a causal relationship with this clinical research.)

3. The research secretariat will report the content reported via the Kanazawa University Clinical Research Electronic Application System to the administrator of Kanazawa University Hospital, the principal investigator, and the test drug manufacturer.
4. Other principal investigators will report to the administrator of the implementing medical institution to which they belong.
5. The administrator of the implementing medical institution will take necessary measures regarding the adverse event.
6. The secretariat of the Certified Clinical Research Review Committee will report the opinion of the Certified Clinical Research Review Committee to the research secretariat, and the research secretariat will report the content of that report to the administrator of Kanazawa University Hospital, the principal investigator, and the investigational drug manufacturer.
7. For further details of procedures related to adverse events, follow the procedures for serious adverse events established by each implementing institution.

**<Emergency Contact>** Principal Investigator: Megumi Oshima Department of Laboratory Medicine, Kanazawa University Hospital Address: 13-1 Takara-machi, Kanazawa, Ishikawa Phone: 076-265-2499

### 8.4.2. Reporting to the Minister of Health, Labour and Welfare, etc.

If an unexpected serious adverse event suspected to be caused by the implementation of this clinical research occurs, the principal investigator will submit a "Disease Report (Pharmaceutical)" (Separate Form 2-1) to the Minister of Health, Labour and Welfare within 7 days if it is related to death or the risk of death, and within 15 days for other cases, from the day they become aware of its occurrence.

### 8.4.3 Detailed Report and Additional Report

If additional information is obtained regarding a serious adverse event reported to the Certified Clinical Research Review Committee, the principal investigator will submit a follow-up report of the "Pharmaceutical Disease Report" and a follow-up report of the "Disease Report" to the Certified Clinical Research Review Committee, and perform 8.4.1. 3) to 7). The principal investigator will also submit a follow-up report of the "Disease Report" to the Minister of Health, Labour and Welfare. If the outcome of the adverse event is "not recovered" or "unknown," follow-up observation will be performed as much as possible.

# 9. Definition of Endpoints

## 9.1. Primary Endpoint

Change in HOMA-IR from before administration to day 5 of administration

Definition: HOMA-IR calculation formula HOMA-IR = Fasting plasma insulin value (μU/mL) × Fasting blood glucose value (mg/dL) / 405

Rationale: HOMA-IR is used as a general indicator of insulin resistance.

## 9.2. Secondary Endpoints

1. Changes in the following items from before administration to day 5 of administration and 5 days after the end of administration:

- Blood glucose, insulin, HbA1c, C-peptide
- Blood pressure, BMI
- eGFR

eGFR calculation formula: eGFR (mL/min/1.73 $m^2$) = 194 × Serum creatinine concentration (mg/dL)^-1.094^ × Age (years)^-0.287^ (For women, × 0.739) Calculated with the age at the date of consent.

- Urine albumin-creatinine ratio
- Chiral amino acids (blood, urine, feces, saliva)
- Intestinal bacteria 16S rRNA flora analysis (feces, saliva)

2. Occurrence of adverse events

Rationale: Blood glucose and HbA1c are common indicators of blood glucose management in diabetic patients, and insulin and C-peptide are used as indicators of insulin secretion. Poor blood pressure management, obesity, decreased eGFR, and increased urine albumin-creatinine ratio are indicators of diabetic complications. 16S rRNA flora analysis is a method to analyze the types and distribution of bacteria contained in specimens by amplifying the 16S rRNA gene that bacteria have with PCR and using a next-generation sequencer, enabling comprehensive evaluation of intestinal bacteria. Chiral amino acids are metabolites of intestinal bacteria, and by evaluating them together with 16S rRNA flora analysis, detailed analysis of changes in the intestinal environment becomes possible.

# 10. Statistical Considerations

## 10.1. Rationale for Target Sample Size

This clinical research is a pilot study targeting healthy individuals, and the sample size was set as the number of cases that can be implemented within the research period. With this number of cases, when examining the difference in the primary endpoint, HOMA-IR, a difference of 0.6 in the change from baseline between the risperidone group and the non-administration group, with a standard deviation of 0.5 (effect size 1.2), can maintain a detection power of 80% under a two-sided significance level of 5%.

## 10.2. Analysis Population

The analysis of the primary endpoint and secondary endpoints will be primarily performed on the Full Analysis Set (FAS). An analysis will also be performed on the Per Protocol Set (PPS) to confirm the stability of the analysis results.

FAS is defined as the population excluding cases that fall under any of the following from all randomized (registered) cases: 1) cases that are determined to be outside the target disease by definitive diagnosis, 2) cases that violate clearly defined objectively determinable important selection/exclusion criteria, 3) cases that have never taken the test drug since registration, 4) cases with no data at all since registration.

PPS is defined as cases that meet the minimum requirements for the main variables of the research protocol, complete the protocol treatment, and do not have significant protocol violations related to eligibility criteria, protocol treatment, prohibited concomitant drugs, etc.

## 10.3. Analysis Items/Methods

### 10.3.1. Main Analysis Method for the Primary Endpoint

Using FAS as the target, a Student's t-test will be performed for the null hypothesis that the change in HOMA-IR from baseline at the end of protocol treatment (5 days after the start of medication) is equal between the risperidone group and the non-administration group.

### 10.3.2. Main Secondary Analysis Method for the Primary Endpoint

Using FAS as the target, the means of the change in HOMA-IR from baseline at the end of test drug administration (5 days after the start of medication) will be compared after adjustment for background factors of subjects such as age, gender, and renal function.

### 10.3.3. Analysis Method for Secondary Endpoints

Using FAS as the target, a Student's t-test will be performed for the null hypothesis that the changes in blood glucose, insulin, HbA1c, C-peptide, blood pressure, BMI, eGFR, urine albumin-creatinine ratio, chiral amino acids, and 16S rRNA from baseline at the end of protocol treatment (5 days after the start of medication) are equal between the risperidone group and the non-administration group.

For the occurrence of adverse events, using FAS as the target, a chi-square test will be performed for the null hypothesis that the occurrence is equal between the risperidone group and the non-administration group at the end of protocol treatment (5 days after the start of medication).

### 10.3.4. Significance Level

The significance level will be 5% two-sided.

#### 10.3.5. Handling of Missing Data, etc.

Missing values will not be imputed and will be analyzed as missing.

## 10.4. Interim Analysis

In this clinical research, an interim analysis will be conducted to determine the need to terminate the research early from the perspective of subject safety. The interim analysis will be conducted when half of the target sample size has completed the protocol treatment. The principal investigator will determine whether to discontinue this clinical research by looking at the results of adverse events of risperidone in the test drug group.

## 10.5. Procedures for Changes to the Statistical Analysis Plan

When changing the contents described in "9. Definition of Endpoints" and "10. Statistical Considerations," the changes will be made according to "15.2. Revision of the Research Protocol." Changes to the statistical analysis plan other than these will be made according to the statistical analysis plan.

# 11. Completion and Submission of Case Report Forms

## 11.1. Types and Submission Deadlines

The case report form will include the following entries:

- Consent obtained
- Background of the research subject
- Test results
- Information on test drug administration, etc.
- Information on adverse events (seriousness, degree, outcome, end date)
- Discontinuation date, reason for discontinuation, adverse event that caused discontinuation, course after discontinuation, and results of follow-up investigation
- Principal (sub-)investigator comments

## 11.2. Recording Method

The principal (sub-)investigator or research collaborator will record the CRF according to the following regulations. The CRF will be recorded according to the "Guide for Creating and Modifying Case Report Forms" provided separately.

(1) Entries in the CRF will be made by the principal (sub-)investigator, etc. (2) CRFs will be created for cases where registration has been completed. (3) When changing or correcting the contents of the CRF, the person making the correction, the date of correction, and the reason for the change or correction will be recorded. (4) The principal investigator will sign the CRF after confirming that it has been created accurately and completely. (5) The principal investigator will keep a copy of the CRF.

## 11.3. Sending Method

Not applicable as this is a single-center study.

# 12. Access to Source Documents, etc., and Quality Control and Quality Assurance

## 12.1. Acceptance of Direct Access and Cooperation

The principal investigator and the implementing medical institution will make all clinical research-related records, including source documents, etc., available for direct access and will cooperate with monitoring, as well as investigations by the Certified Clinical Research Review Committee and regulatory authorities related to this clinical research.

## 12.2. Monitoring

The principal investigator will appoint a person to be in charge of monitoring. The monitoring personnel will conduct monitoring by directly reviewing source documents from the following perspectives:

- Whether the human rights protection and safety of the clinical research subjects are ensured
- Whether the clinical research is being conducted in compliance with the latest implementation plan, research protocol, and this rule
- Whether written consent has been obtained from the clinical research subjects regarding the implementation of clinical research
- Whether records, etc., are accurate

The monitoring personnel will perform their duties according to the monitoring plan formulated separately to confirm that this clinical research is being conducted in accordance with the research protocol and the Clinical Trials Act.

# 13. Ethical Matters

## 13.1. Regulations to be Observed

All those involved in this clinical research will comply with the "World Medical Association Declaration of Helsinki," to which all medical research involving humans should adhere, and the Clinical Trials Act and related ministerial ordinances and notifications, after thoroughly reading and understanding their contents.

## 13.2. Review by the Certified Clinical Research Review Committee and Notification to the Head of the Implementing Medical Institution and the Minister of Health, Labour and Welfare Prior to the Start of Research

Before conducting this clinical research, the principal investigator will create this research protocol and implementation plan (Form 1), explanatory document/consent form, documents describing the overview of pharmaceuticals, etc., procedures for handling suspected diseases, etc., that occur due to the implementation of this clinical research, conflict of interest management standards and conflict of interest management plan, document listing the names of the principal investigator and sub-investigators (Unified Form 1), statistical analysis plan if created, and other documents requested by the Certified Clinical Research Review Committee, submit them to the Certified Clinical Research Review Committee, and seek opinions. The principal investigator will submit the documents submitted to the Certified Clinical Research Review Committee and the opinions of the Certified Clinical Research Review Committee to the head of the implementing medical institution to obtain approval for the implementation of clinical research. The principal investigator will start the clinical research after submitting the "Implementation Plan" (Form 1) reflecting the opinions of the Certified Clinical Research Review Committee to the Minister of Health, Labour and Welfare.

## 13.3. Creation and Revision of Explanatory Document/Consent Form (Format)

The explanatory document/consent form will be created by the principal investigator and revised as necessary. Also, the created or revised explanatory document/consent form will be submitted to the Certified Clinical Research Review Committee in advance. It will also be reported to the head of the implementing medical institution along with the opinion of the Certified Clinical Research Review Committee, and approval will be obtained.

The explanatory document must include at least the following items specified in the "Clinical Trials Act Enforcement Regulations." It must not contain descriptions that intentionally guide research subjects.

- The name of the specific clinical research to be conducted, the fact that approval has been obtained from the head of the implementing medical institution for the implementation of

the specific clinical research, and the fact that an implementation plan has been submitted to the Minister of Health, Labour and Welfare

- The name of the implementing medical institution and the name and title of the principal investigator
- The reason for being selected as a subject for specific clinical research
- Expected benefits and disadvantages from the implementation of specific clinical research
- The fact that refusal to participate in specific clinical research is voluntary
- Matters related to withdrawal of consent
- The fact that no disadvantageous treatment will be received due to refusing to participate in specific clinical research or withdrawing consent
- Method of information disclosure regarding specific clinical research
- The fact that the subject of specific clinical research or their legal representative can obtain or view the research protocol and other materials related to the implementation of specific clinical research upon request, and the method of obtaining or viewing
- Matters related to the protection of personal information of the subject of specific clinical research
- Method of storage and disposal of samples, etc.
- Status of research funding, etc., provided by pharmaceutical product manufacturers, etc., for specific clinical research, and other involvement, and status of involvement such as the provision of donations, manuscript writing, and remuneration for lectures and other duties by pharmaceutical product manufacturers, etc., to those engaged in clinical research and those listed in the research protocol
- System for handling complaints and inquiries
- Matters related to costs associated with the implementation of specific clinical research
- Presence and content of other treatment methods, and comparison with expected benefits and disadvantages from other treatment methods
- Matters related to compensation and provision of medical care for health damage caused by the implementation of specific clinical research
- Review items of the Certified Clinical Research Review Committee that performs review opinion work for specific clinical research and other matters related to the Certified Clinical Research Review Committee for the specific clinical research
- Other necessary matters related to the implementation of specific clinical research

If the principal investigator obtains new findings related to the consent of research subjects after the start of clinical research and determines that a change in the explanatory document/consent form is necessary, the principal investigator will revise it and submit it to the Certified Clinical Research Review Committee. It will also be reported to the head of the implementing medical institution along with the opinion of the Certified Clinical Research Review Committee, and approval will be obtained.

New findings refer to new safety information or information related to the development of new treatment methods for the disease.

# 13.4. Informed Consent

An explanation of the clinical research will be provided, sufficient time will be given to consider, and after confirming that the research subject understands the content of the clinical research well, they will be asked to participate in the clinical research. If the research subject themselves consent to participate in the clinical research, a consent form will be used to obtain the signature of the research subject themselves. The principal investigator or sub-investigator will confirm that the consent form contains the name of the physician who provided the explanation and the date of explanation, the name of the research subject who received the explanation and consented, and the date of consent.*,**

The consent form will be copied in two copies, one will be handed to the research subject themselves, and one will be kept by the principal investigator. The original will be kept in the storage place specified by the research institution.

In this clinical research, personal information obtained may be used for future research that is not specified at the time of obtaining consent, so consent for this will be obtained, and when conducting future clinical research, the research protocol will be submitted to the ethics review committee for review, and reconfirmation regarding consent will be performed.

*The explanatory document must not contain descriptions that waive the rights of the research subject or potential research subject, or descriptions that suggest it, nor descriptions that exempt or reduce the responsibility of the sponsor, the person conducting the trial themselves, the implementing medical institution, the principal investigator, etc., or descriptions that suggest it.

**The explanatory document must use as plain expressions as possible.

## 13.5. Secondary Use of Samples/Information

The samples/information of research subjects obtained in this clinical research may be used for future research that is not specified at the time of obtaining consent. In that case, after receiving a review from an ethics review committee appropriate for the research content regarding conducting research using the samples obtained in this clinical research, it will be implemented after explaining to the research subjects separately.

# 14. Handling of Personal Information

When handling materials, etc., related to the implementation of clinical research, the personal information of research subjects will be managed with unrelated numbers, and sufficient consideration will be given to protecting the secrets of research subjects. When publishing the results of clinical research, information that can identify research subjects will not be included. Also, the samples, etc., of research subjects obtained in the research will not be used for purposes other than the purpose of clinical research.

# 15. Deviation, Change, and Revision of Research Protocol

## 15.1. Deviation or Change of Research Protocol

The principal investigator or sub-investigator must not deviate from or change the research protocol before obtaining the approval of the hospital director based on the prior review of the Certified Clinical Research Review Committee.

The principal investigator or sub-investigator may deviate from or change the research protocol without obtaining the prior approval of the Certified Clinical Research Review Committee for unavoidable reasons such as emergency avoidance. In such cases, the principal investigator or sub-investigator will promptly submit the content and reason for the deviation or change, and the draft of the revision of the research protocol, etc., if necessary, to the Certified Clinical Research Review Committee and obtain the approval of the Certified Clinical Research Review Committee and the hospital director.

The principal investigator or sub-investigator must record all deviation items from the research protocol along with their reasons if there is a deviation from the research protocol.

## 15.2. Revision of Research Protocol

When revising the research protocol, the principal investigator will decide on the revision after discussing with the statistical analysis responsible person, etc., as necessary, regarding the validity of the change and the impact on the evaluation of clinical research. When revising, the revised research protocol and, if necessary, the revised explanatory document/consent form will be submitted to the Certified Clinical Research Review Committee to seek opinions. Also, the revised research protocol and explanatory document/consent form, along with the opinion of the Certified Clinical Research Review Committee, will be reported to the head of the implementing medical institution to obtain approval. In the case of significant changes, temporary suspension of case registration will be considered from the perspective of research subject protection. When changing the conflict of interest management standards or conflict of interest management plan, the opinion of the Certified Clinical Research Review Committee and the approval of the head of the implementing medical institution will be obtained in the same way as when revising the research protocol.

After obtaining approval for the revision, the principal investigator will promptly communicate the revised content to the sub-investigators, data center, etc., and if the revision involves a change in the research protocol, submit an Implementation Plan Item Change Notification (Form 2) to the Minister of Health, Labour and Welfare. If the revision of the research protocol affects the content of the case registration form or case report form, those parts will be promptly revised.

In the case of minor changes to the implementation plan (changes in name without changes in clinical research staff, or changes in place names or street numbers (changes in regional names, not relocations)), the content will be notified to the Certified Clinical Research Review Committee and the head of the implementing medical institution, and an Implementation Plan Item Minor Change Notification (Form 3) will be submitted to the Minister of Health, Labour and Welfare within 10 days from the date of change.

# 16. Completion and Early Termination of Clinical Research

## 16.1. Completion of Clinical Research

The principal investigator will create a comprehensive report and its summary within one year, in principle, after the completion of collection of all data on all evaluation items described in the research protocol, and submit them to the Certified Clinical Research Review Committee to seek opinions. The comprehensive report and its summary, along with the opinion of the Certified Clinical Research Review Committee, will also be reported to the head of the implementing medical institution to obtain approval. The principal investigator will submit a "Completion Notification" (Separate Form 1) to the Minister of Health, Labour and Welfare and publish the summary of the comprehensive report on jRCT within one month from the date the Certified Clinical Research Review Committee expressed its opinion. Regarding the publication of the summary of the comprehensive report by jRCT, if the results of this clinical research are to be published in papers, etc., the principal investigator will report to the Certified Clinical Research Review Committee that the paper is being submitted, and then publish on jRCT after the publication of the paper.

## 16.2. Early Termination of Clinical Research

This clinical research will be terminated if any of the following is recognized:

Superiority or inferiority in the efficacy of the investigational drug was confirmed by interim analysis.

It was found from interim analysis that the probability of proving the superiority of the investigational drug is small.

Based on the results of interim analysis, serious adverse event reports, or information other than this clinical research, it was determined that there is a problem with the safety of the investigational drug.

The Certified Clinical Research Review Committee recommended or instructed termination, or the principal investigator determined that termination is appropriate, in light of the safety of this clinical research and the status of deviations from authority regulations or the research protocol.

The Certified Clinical Research Review Committee recommended or instructed termination, or the principal investigator determined that termination is appropriate, because it was thought that there is a problem with the safety of the investigational drug based on information other than this clinical research.

For other reasons, such as delays in case registration or frequent deviations from the research protocol, it was determined that completion of clinical research is difficult.

The principal investigator will consider whether it is possible to continue the implementation of clinical research if any of the following applies:

When important information regarding the quality, safety, or efficacy of the investigational drug is obtained

When it is determined that it is extremely difficult to achieve the planned number of cases because recruitment of research subjects is difficult

When the purpose of clinical research is achieved (e.g., by interim analysis) before reaching the planned number of cases or planned period

When there is an instruction from the Certified Clinical Research Review Committee to change the implementation plan, etc., and it is determined that it is difficult to accept this

If the principal investigator believes that this clinical research should be terminated, they will take necessary measures for the research subjects. If necessary, the opinion of the Certified Clinical Research Review Committee will be sought regarding the timing and method of the clinical research termination accompanying the measures for the research subjects. The principal investigator will submit a "Discontinuation Notification" (Unified Form 11) to the Certified Clinical Research Review Committee and a "Specific Clinical Research Discontinuation Notification" (Form 4) to the Minister of Health, Labour and Welfare. Even after submitting the "Specific Clinical Research Discontinuation Notification" to the Minister of Health, Labour and Welfare, periodic reports will continue to be submitted until the clinical research is completed, and if it corresponds to a change in matters related to the progress status of the clinical research, a notification of change in the implementation plan will be made.

# 17. Reporting to the Head of the Implementing Medical Institution, etc.

## 17.1. Matters to be Reported to the Head of the Implementing Medical Institution

The principal investigator must report the following events to the head of the implementing medical institution to which they belong:

- Non-compliance (if there is concern that it will not be reported from the principal investigator to the head of the implementing medical institution, it will be reported from the sub-investigator to the head of the implementing medical institution)
- Provision of research funds, etc., by pharmaceutical manufacturers, etc., and other involvement
- Opinion of the Certified Clinical Research Review Committee
- Summary of comprehensive report
- Publication of the summary of comprehensive report
- Submission of implementation plan
- Occurrence of serious adverse events thought to be due to the implementation of clinical research and their subsequent course
- Malfunctions
- Disease, etc., report
- Periodic report

The principal investigator must obtain approval in addition to reporting to the head of the implementing medical institution to which they belong for the following events:

- Whether clinical research can be implemented
- Obtaining consent from minors aged 16 or older who meet specific conditions (without a legal representative)

Sub-investigators, etc., will promptly report to the principal investigator if they become aware of non-compliance.

## 17.2. Matters to be Reported to the Certified Clinical Research Review Committee

The principal investigator must seek opinions from or report to the Certified Clinical Research Review Committee regarding the following events:

- Start of research
- Occurrence of serious adverse events thought to be due to the implementation of clinical research and their subsequent course
- Disease, etc., report
- Periodic report
- Change in implementation plan
- Change in research protocol, conflict of interest management standards, conflict of interest management plan
- Discontinuation of clinical research (as necessary)
- Significant non-compliance
- Summary of comprehensive report, publication (jRCT registration)
- Obtaining consent from minors aged 16 or older who meet specific conditions (without a legal representative)

The principal investigator must notify the Certified Clinical Research Review Committee of the following events:

- Submission of implementation plan to the Minister of Health, Labour and Welfare

## 17.3. Matters to be Reported to the Minister of Health, Labour and Welfare

The principal investigator must report the following events to the Minister of Health, Labour and Welfare (Director of the Regional Bureau of Health and Welfare):

- Implementation plan (start of research)
- Change in implementation plan
- Minor change in implementation plan
- Discontinuation of clinical research
- Completion of clinical research
- Periodic report

The principal investigator must report the following events to the Minister of Health, Labour and Welfare (President of the Pharmaceuticals and Medical Devices Agency):

- Disease, etc., report (information on unexpected serious adverse events thought to be due to the implementation of clinical research in specific clinical research including unapproved drugs/off-label use)

# 18. Handling of Records Related to Clinical Research

The principal investigator will appropriately preserve documents related to the implementation of clinical research, etc. (copies of application documents, notification documents from the Certified Clinical Research Review Committee, notification documents from the head of the implementing medical institution, copies of various application forms/reports, research subject identification code list, (screening list), documents related to consent, documents related to registration, documents related to response to diseases, etc., copies of case report forms, contracts related to clinical research implementation, records related to the acquisition and use, disposal, etc., of investigational drugs, investigational medical devices, investigational regenerative medicine products, various procedure manuals, monitoring reports, and other documents or records necessary to guarantee the reliability of data, etc.) for a period of 5 years from the later of the date the summary of the comprehensive report was published on jRCT or the date the final publication (paper, etc.) of the results of the clinical research was made, and then dispose of them with attention to personal information.

Medical records will be stored and disposed of according to the regulations of the hospital.

- Research protocol, implementation plan, documents related to explanation and consent to the subjects of specific clinical research, comprehensive report, and other documents created by the principal investigator according to the provisions of this ordinance, or copies thereof
- Documents related to review opinion work received from the Certified Clinical Research Review Committee
- Documents related to monitoring
- Source documents, etc.
- Contract documents related to the implementation of specific clinical research
- Documents describing the overview of pharmaceuticals, etc., used in specific clinical research, and records of the acquisition and disposal of pharmaceuticals, etc.

# 19. Payment and Compensation Related to the Implementation of Clinical Research

## 19.1. Costs Related to Clinical Research

The test drug risperidone in this clinical research will be paid from the grants from the clinical research group and the 2021 Clinical Research Grant of Kanazawa University Hospital.

Stipends for research subjects (5,000 yen per person per visit, paid with a Quo Card, up to a total of 20,000 yen) will be paid from the grants from the clinical research group and the 2021 Clinical Research Grant of Kanazawa University Hospital.

## 19.2. Compensation for Health Damage

If health damage occurs due to this clinical research, appropriate treatment will be provided. In the case of health damage corresponding to death or disability grades 1 and 2 as defined by the Relief System for Adverse Drug Reactions, it will be covered by the "Clinical Research Compensation Liability Insurance" that the clinical research group has joined through the 2021 Clinical Research Grant of Kanazawa University Hospital.

# 20. Research Funding and Conflict of Interest Management

## 20.1. Conflict of Interest Management

The principal investigator, sub-investigators, and statistical analysis responsible person have no conflicts of interest for this clinical research.

## 20.2. Research Funding Source

This clinical research will be conducted with grants from the research group and the 2021 Clinical Research Grant of Kanazawa University Hospital.

# 21. Attribution of Research Results and Publication of Results

This clinical research will be registered and published on jRCT before the start of clinical research. Also, regarding the summary of the comprehensive report, it will be registered and published on jRCT within one month from the date the opinions of the Certified Clinical Research Review Committee were sought. Regarding the publication of the summary of the comprehensive report by jRCT, if the results of this clinical research are to be published in papers, etc., the principal investigator will report to the Certified Clinical Research Review Committee that the paper is being submitted, and then publish on jRCT after the publication of the paper.

The results of this clinical research will belong to the research group. The principal investigator and the statistical analysis responsible person will discuss and select authors, and report at academic conferences or in papers.

# 22. Research Organization

## 22.1. Principal Investigator

Megumi Oshima, Assistant Professor, Department of Laboratory Medicine, Kanazawa University Hospital Address: 13-1 Takara-machi, Kanazawa, Ishikawa 920-8641 Phone: 076-265-2499

## 22.2. Research Secretariat (Coordination Management Practitioner)

Department of Nephrology, Kanazawa University Hospital, Administration Address: 13-1 Takara-machi, Kanazawa, Ishikawa 920-8641 Phone: 076-265-2499 FAX: 076-234-4273 E-mail: lab-med@med.kanazawa-u.ac.jp

## 22.3. Data Management Responsible Person

Shizuko Takahara, Data Center Division, Innovative Clinical Research Center, Kanazawa University Hospital Address: 13-1 Takara-machi, Kanazawa, Ishikawa 920-8641 Phone: 076-265-2873

## 22.4. Statistical Analysis Responsible Person

Naoshi Tohyama, Biostatistics Division, Innovative Clinical Research Center, Kanazawa University Hospital Address: 13-1 Takara-machi, Kanazawa, Ishikawa 920-8641 Phone: 076-265-2090

## 22.5. Monitoring Responsible Person

Shuji Sugimoto, Monitoring and Audit Division, Innovative Clinical Research Center, Kanazawa University Hospital Address: 13-1 Takara-machi, Kanazawa, Ishikawa 920-8641 Phone: 076-265-2878

## 22.6. Investigational Drug Manager

Katsuhiko Nagase, Clinical Research Promotion Division, Innovative Clinical Research Center, Kanazawa University Hospital Address: 13-1 Takara-machi, Kanazawa, Ishikawa 920-8641 Phone: 076-265-2049

## 22.7. Case Registration Center

Data Center Division, Innovative Clinical Research Center, Kanazawa University Hospital Address: 13-1 Takara-machi, Kanazawa, Ishikawa 920-8641 Phone: 076-265-2090 FAX: 076-234-4346 (Reception time: 9-17 on weekdays (excluding holidays)) Responsible person: Shizuko Takahara

Commissioned tasks: Receipt and content confirmation of case registration forms, assignment of registration numbers and issuance of content confirmation forms, management of registration information

## 22.8. Specimen Test Measurement Center

BML Co., Ltd. Kanazawa Branch Address: 1-52 Saito, Kanazawa, Ishikawa 920-8202 Phone: 076-266-0600

Commissioned test content: Blood tests (white blood cell count, red blood cell count, hemoglobin, hematocrit, platelet count, TP, Alb, AST, ALT, BUN, Cre, UA, Na, K, Ca, P, Mg, fasting blood glucose, HbA1c, T-Cho, HDL-Cho, TG, insulin, C-peptide), urine tests (glucose, occult blood, Alb, Cre)

KAGAMI Co., Ltd. Address: 7-7-18-308 Saito Asagi, Ibaraki, Osaka 567-0085 Saito Bio Hills Center, Room 308 Phone: 072-646-7059 Contact person: Masafumi Mita Commissioned test content: Chiral amino acids

Takara Bio Inc. Address: 7-4-38 Nojihigashi, Kusatsu, Shiga 525-0058 Phone: 077-565-6920 Commissioned test content: 16S rRNA flora analysis

# 23. Complaint and Consultation Window

The research secretariat will respond as a contact point for complaints and consultations from research subjects and their related parties.

Address: 13-1 Takara-machi, Kanazawa, Ishikawa 920-8641 Contact person: Megumi Oshima Phone: 076-265-2499 FAX: 076-234-4273

# 24. References


1. Koppe L, Mafra D, Fouque D. 2015. Probiotics and chronic kidney disease. Kidney Int. 88(5):958-66.
2. Nakade Y, Iwata Y, Furuichi K, et al. 2018. Gut microbiota-derived D-serine protects against acute kidney injury. JCI Insight. 18;3(20). pii: 97957.
3. Kimura T, Hamase K, Miyoshi Y, et al. 2016. Chiral amino acid metabolomics for novel biomarker screening in the prognosis of chronic kidney disease. Sci Rep. 18;6:26137.


# 25. Appendices

1. PMDA materials (Risperdal)
2. Package insert (Risperdal)
