# Supplementary figures and images for: Effects of risperidone on amino acid metabolism, glucose, and kidney function in healthy adults: A pilot randomized controlled trial
Source: PLoS One. 2025 Dec 5;20(12):e0324222. doi: 10.1371/journal.pone.0324222 (PMC12680216; doi:10.1371/journal.pone.0324222)

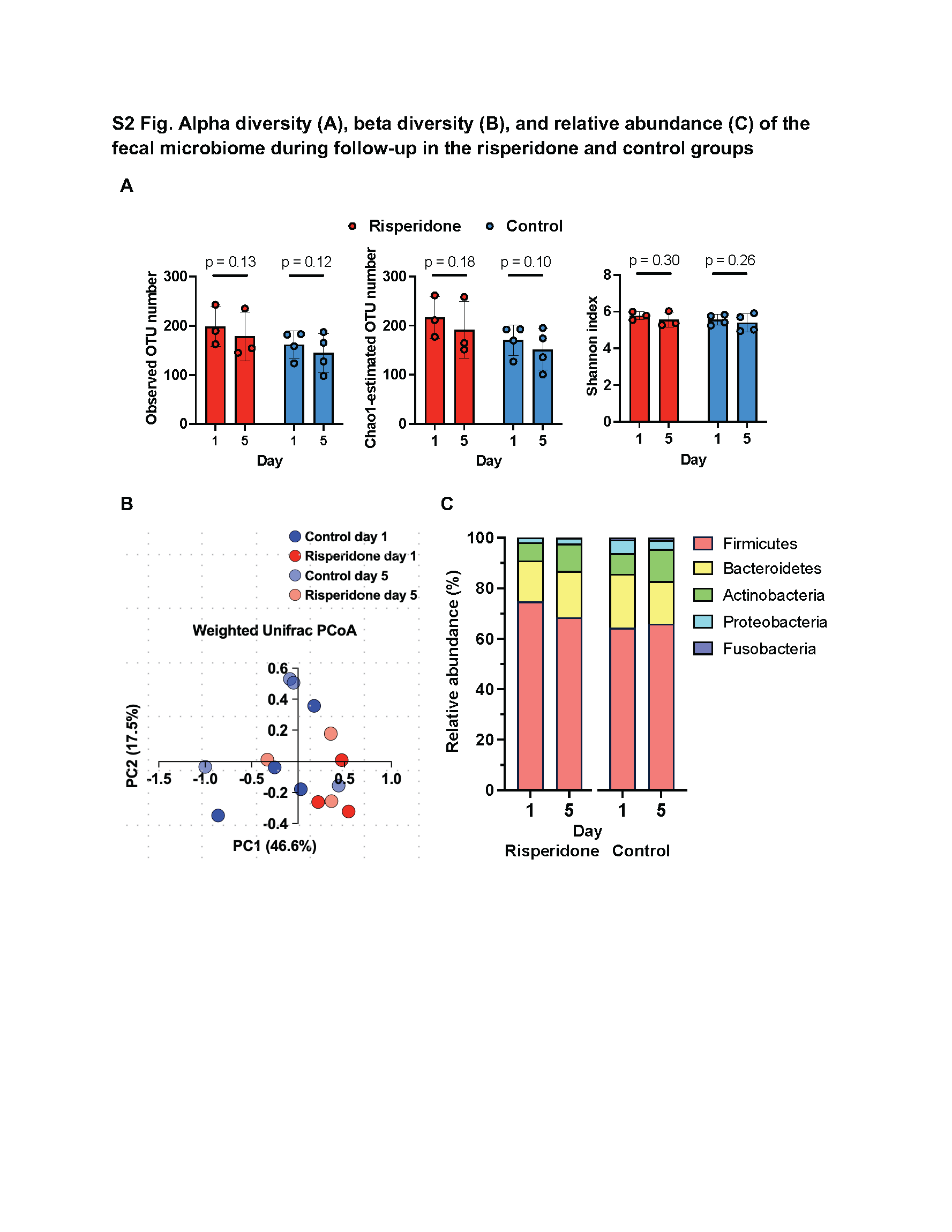

Supplement: S2 Fig — (TIFF) [file pone.0324222.s004.tiff]

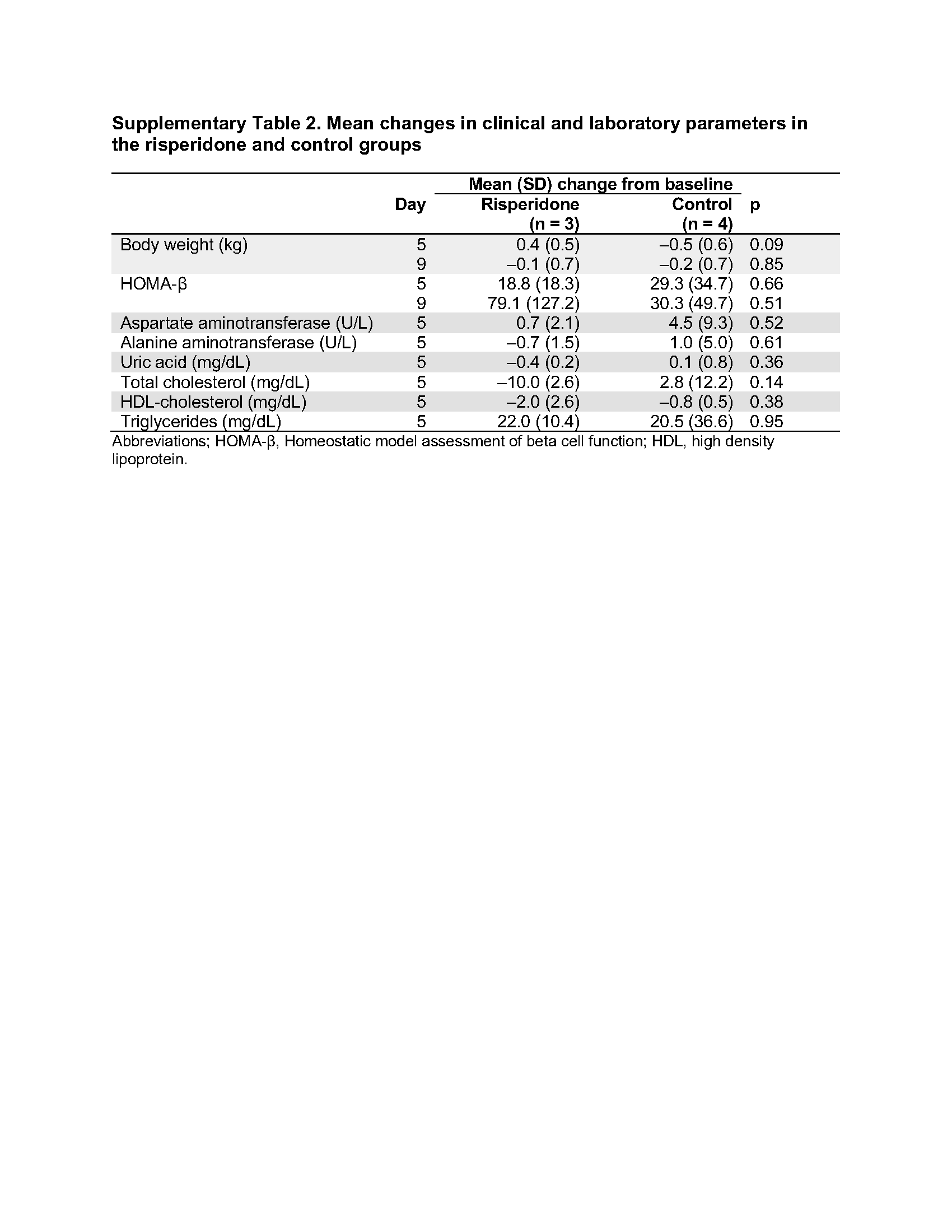

Supplement: S2 Table — Abbreviations; HOMA-β, Homeostatic model assessment of beta cell function; HDL, high density lipoprotein. (TIFF) [file pone.0324222.s006.tiff]

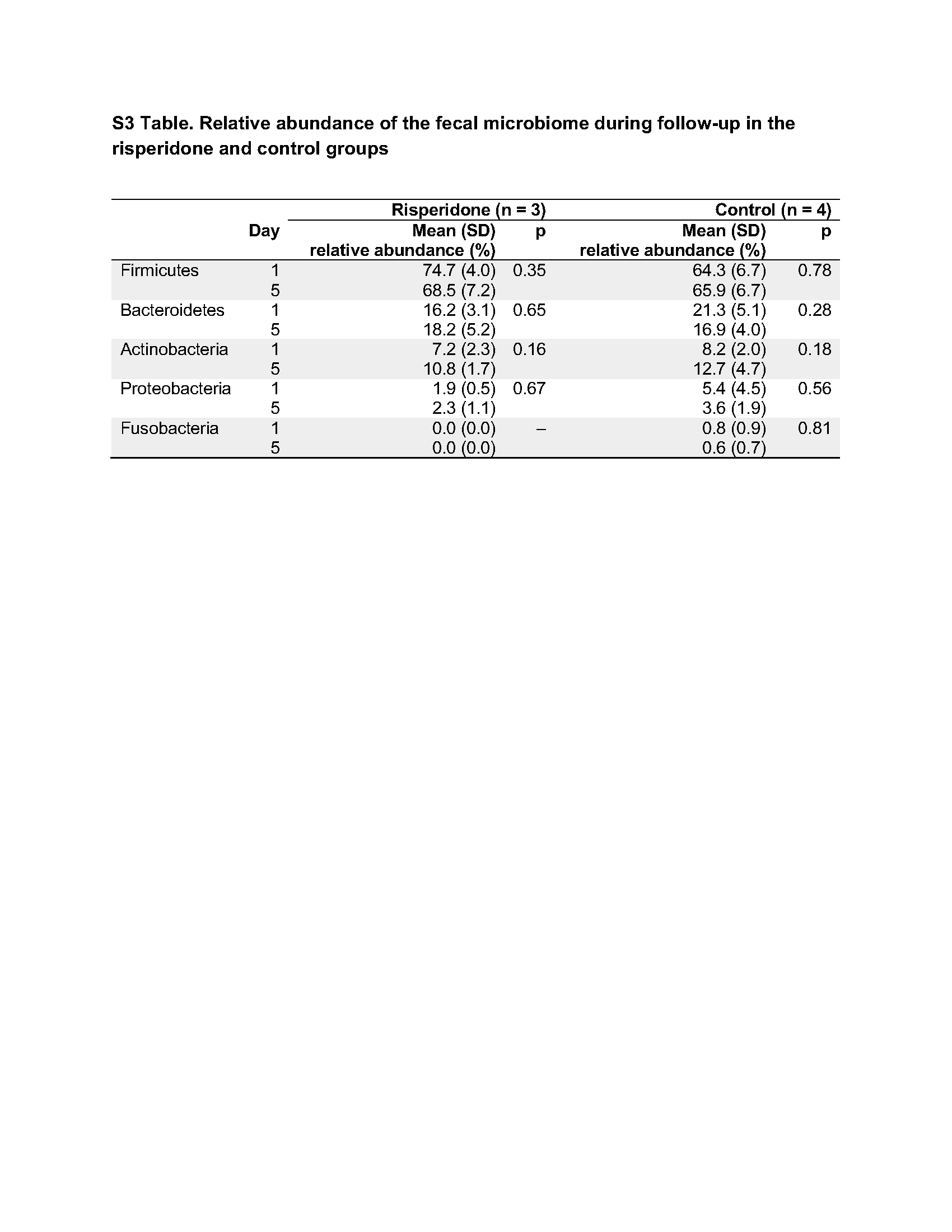

Supplement: S3 Table — (TIFF) [file pone.0324222.s007.tiff]
