# Supplementary material for: Effects of risperidone on amino acid metabolism, glucose, and kidney function in healthy adults: A pilot randomized controlled trial
Source: PLoS One. 2025 Dec 5;20(12):e0324222. doi: 10.1371/journal.pone.0324222 (PMC12680216; doi:10.1371/journal.pone.0324222)
Supplement: S1 Table — Abbreviations; Cr, creatinine. (PDF) [file pone.0324222.s005.pdf]

S1 Table. Mean changes in plasma and urine DL-serine and alanine levels in the risperidone and control groups

		Mean (SD) change from baseline		Between groups		Within-group p	
	Day	Risperidone (n = 3)	Control (n = 4)	p*	p**	Risperidone	Control
**Plasma amino acid**							
D-serine (nmol/ml)	5	0.24 (0.36)	–0.32 (0.13)	0.03	0.03	0.36	0.02
L-serine (nmol/ml)	5	2.93 (15.28)	–12.65 (29.22)	0.44	0.29	0.77	0.45
D/L-serine (%)	5	0.12 (0.05)	–0.11 (0.23)	0.16	0.03	0.06	0.42
D-alanine (nmol/ml)	5	0.54 (1.03)	–0.31 (0.70)	0.25	0.40	0.46	0.44
L-alanine (nmol/ml)	5	–24.67 (44.09)	–23.25 (69.26)	0.98	0.43	0.43	0.55
D/L-alanine (%)	5	0.17 (0.24)	–0.09 (0.28)	0.25	0.13	0.33	0.57
**Urine amino acid**							
D-serine/Cr (mmol/g Cr)	5	38.16 (19.77)	–25.84 (23.41)	0.01	0.03	0.08	0.11
L-serine/Cr (mmol/g Cr)	5	53.57 (95.66)	–70.40 (47.96)	0.07	0.08	0.43	0.06
D-alanine/Cr (mmol/g Cr)	5	24.98 (38.59)	–9.42 (23.35)	0.20	0.16	0.38	0.49
L-alanine/Cr (mmol/g Cr)	5	32.20 (52.86)	11.85 (71.62)	0.70	1.00	0.40	0.76

Abbreviations; Cr, creatinine. P* values were calculated using Student's t-test and p** values were calculated using Mann-Whitney U test as non-parametric analysis between groups. Within-group p values were calculated using paired t-tests comparing day 5 to baseline within each group.
