# Supplementary material for: Effects of risperidone on amino acid metabolism, glucose, and kidney function in healthy adults: A pilot randomized controlled trial
Source: PLoS One. 2025 Dec 5;20(12):e0324222. doi: 10.1371/journal.pone.0324222 (PMC12680216; doi:10.1371/journal.pone.0324222)
Supplement: Attachment — Submitted filename: PONE-D-25-19801_R1.pdf [file pone.0324222.s009.pdf]

PLOS ONE

Effects of risperidone on amino acid metabolism, glucose, and kidney function in healthy adults: A pilot randomized controlled trial

--Manuscript Draft--

Manuscript Number:	PONE-D-25-19801R1
Article Type:	Clinical Trial
Full Title:	Effects of risperidone on amino acid metabolism, glucose, and kidney function in healthy adults: A pilot randomized controlled trial
Short Title:	Effects of risperidone on amino acid metabolism
Corresponding Author:	Yasunori Iwata<br>Kanazawa University: Kanazawa Daigaku<br>Kanazawa, JAPAN
Keywords:	D-amino acid; risperidone; kidney
Abstract:	d-serine administration prevents kidney damage in murine models of acute kidney injury, and risperidone inhibits the activity of d-amino acid oxidase, which regulate plasma d-amino acid levels. This pilot randomized controlled trial investigated the effects of risperidone on glucose, amino acid metabolism, and kidney function in healthy adults. Healthy adults with a homeostasis model assessment of insulin resistance (HOMA-IR) of ≥ 1.6 and estimated glomerular filtration rate (eGFR) of ≥ 60 mL/min/1.73m2 were randomly assigned to the risperidone and control groups. The risperidone group received 0.5 mg/day risperidone for 4 days. The primary outcome was mean change in HOMA-IR on day 5, and the secondary outcomes were changes in d-amino acid levels, eGFR, and urinary albumin. Seven participants were randomized to the risperidone and control groups. The changes in HOMA-IR, eGFR, and urinary albumin on day 5 were not significantly different between the two groups (all p>0.05). Mean changes in plasma d-serine level and urinary d-serine/creatinine ratio were significantly higher in the risperidone group than in the control group (0.2 vs. −0.3 nmol/mL, p=0.03 and 38.2 vs. −25.8 nmol/mL, p=0.01, respectively). Short-term risperidone affects d-serine metabolism without instigating acute adverse effects on kidney or glucose homeostasis in healthy individuals.<br><br>Clinical Trial Registry number<br><br>This study was registered with the Japan Registry for Clinical Trials (jRCTs041210165).
Order of Authors:	Megumi Oshima
	Tadashi Toyama
	Yusuke Nakade
	Sakae Miyagi
	Hisayuki Ogura
	Shiori Nakagawa
	Takahiro Yuasa
	Akihiko Koshino
	Keisuke Horikoshi
	Taichiro Minami
	Keisuke Sako
	Shunsuke Tsuge
	Akira Tamai
	Ryo Nishioka

Powered by Editorial Manager® and ProduXion Manager® from Aries Systems Corporation

	Taro Miyagawa
	Kiyoaki Ito
	Shinji Kitajima
	Ichiro Mizushima
	Akinori Hara
	Norihiko Sakai
	Miho Shimizu
	Toshiaki Tokumaru
	Makoto Tsubomoto
	Mitsuru Kikuchi
	Masashi Kinoshita
	Mitsutoshi Nakada
	Masashi Mita
	Yasunori Iwata
	Takashi Wada
**Response to Reviewers:**	COMMENTS TO THE AUTHOR:<br><br>Reviewer 1:<br>1. It would be informative to know whether there is any difference in the baseline characters between the two groups.<br><br>Response: Thank you for this important comment. We have added statistical comparisons of baseline characteristics between the two groups to the results section and Table 1 in the revised manuscript.<br><br>Results (page 10):<br>The baseline participant characteristics were comparable between the two groups (all $p > 0.10$), except for body mass index, which was likely to be higher in the risperidone group than in the control group ($p = 0.03$).<br><br>2. The sample size is extremely small. The inference based on this pilot study is questionable to some extent.<br><br>Response: We acknowledge that the small sample size is a significant limitation of this pilot study and have emphasized the limitation in the discussion section in the revised manuscript.<br><br>Discussion (page 15):<br>This study has several important limitations…. Second, we focused exclusively on a healthy population in a pilot study with a small sample size and short follow-up period, which limited the robustness of the safety data. The small sample size limits our findings and any inferences should be interpreted with caution…. Longer-term clinical trials with larger sample sizes in patients with CKD are warranted to assess the sustained effects and safety of risperidone in this patient population.<br><br>3. Student's t-test assumes normality and equal variances. How to check the normality with such a small sample?<br><br>Response: We agree with the reviewer’s comment and have added Mann-Whitney U tests as non-parametric analyses to complement Student's t-tests. The results remained consistent between parametric and non-parametric approaches, except for fasting blood glucose where the Mann-Whitney U test did not show a significant difference. We have added these results and limitation to the revised manuscript.<br><br>Methods, Statistical analysis (page 8):

Powered by Editorial Manager® and ProduXion Manager® from Aries Systems Corporation

….For the primary and secondary endpoints, Student's t-test was used to compare mean changes in clinical and laboratory parameters from baseline to days 5 and 9 between the risperidone and control groups. Given the small sample size, Mann-Whitney U test was also performed as a non-parametric analysis to complement the parametric tests.

Results (page 10):
…. The significant differences in plasma d-serine levels and urinary D-serine-to-creatinine ratio between groups remained consistent when analyzed using Mann-Whitney U tests (S1 Table).

Results (page 11):
…. The mean change in fasting blood glucose was marginally higher in the risperidone group than in the control group by Student's t-test (4.0 ± 2.0 vs. −1.3 ± 3.0 mg/dL, p = 0.048), however this difference was not significant when analyzed using the Mann-Whitney U test (p = 0.07). …. All other parameters except fasting blood glucose showed consistent results between parametric and non-parametric analyses.

Discussion (page 15):
…. Third, the assumption of normality for Student's t-test may not be reliably verified with small sample sizes, which represents a limitation of our statistical approach. ….

4. With such a small sample size, the focus still seems to be on the inference or significance for the current version. However, its effect sizes might be more valuable than determining the significance.

Response: Thank you for this important suggestion. We have added discussion emphasizing the magnitude of effects rather than statistical significance in the discussion section.

Discussion (page 14):
Given the small sample size of this pilot study, the magnitude of observed effects may be more informative than statistical significance. The substantial increase in plasma D-serine levels (0.56 nmol/mL difference, 95% CI 0.07–1.05) and urinary D-serine-to-creatinine ratio (64.0 mmol/g Cr difference, 95% CI 20.8–107.2) represent clinically meaningful changes that warrant further investigation in larger studies. Similarly, the numerical increase observed in plasma D-alanine levels and urinary D-alanine-to-creatinine ratio, while not reaching statistical significance, provide valuable preliminary data for effect size calculations in future trial designs.

Reviewer 2:
1. I think the background could benefit from some general information and references regarding the effects of antipsychotics on kidney function to help link your two topics here. A paragraph would be plenty to highlight current evidence regarding various antipsychotics and their influence on eGFR, CrCl, etc

Response: Thank you for this valuable suggestion. We have added the sentences in the introduction section discussing the current evidence regarding various antipsychotics and their influence on kidney function.

Introduction (page 4-5):
Several studies have investigated the effects of antipsychotic medications on kidney function with mixed results. While some retrospective studies suggest that atypical antipsychotics, including risperidone, may not increase the risk of acute kidney injury compared to typical antipsychotics,[14, 15] other population-based studies have reported increased risks in elderly patients.[16] The mechanisms underlying these effects remain unclear, but may involve direct nephrotoxic effects or indirect effects through metabolic changes.

2. there needs to be several rationale given for methodological choices: why that duration for risp treatment? why sample size even if you did
not perform a power calculation? why 140 as the cutoff for glucose which doesn't appear to rule out pre-diabetes? Why the significant instruction

and concern for the effect of the microbiome which isn't mentioned in the background? This becomes even more important since there is a sudden introduction of microbiome analysis in the methods. This should be in the background with rationale for its study.

Response: We have added detailed rationale for our methodological choices throughout the methods section in the revised manuscript.

Introduction (page 5):
….Therefore, we conducted a pilot randomized controlled trial to investigate the impact of short-term risperidone administration on the amino acid profile, kidney function, and glucose metabolism in healthy adults. Microbiome analysis was included because gut microbiome can metabolize D-amino acids, and we aimed to control for potential confounding effects of microbial D-amino acid metabolizm on our study endpoints.

Methods, Study design and participants (page 6):
….The glucose cutoff was chosen to exclude participants with overt diabetes while including those with potential insulin resistance, as measured by HOMA-IR.
….Sample size calculation was not performed due to the absence of prior data on effect size. This pilot study was designed to generate preliminary data for future power calculations.

Methods, Interventions (page 6):
….The lowest available dosage of 0.5 mg/day was chosen to prioritize participant safety. The 4-day treatment duration was chosen based on pharmacokinetic studies showing that steady-state levels are achieved within 3-4 days, allowing us to observe steady-state effects while minimizing exposure in healthy volunteers.

3. You have some instruction about dietary restrictions in this study, was protein intake a concern given its potential effects on renal labs?

Response: Thank you for this important consideration. We did not specifically restrict protein intake in this study, which is a limitation. We have added this to the limitations section in the revised manuscript.

Discussion (page 15):
…. Fourth, we did not specifically control for protein intake during the study period, which may have influenced renal laboratory parameters and represents a limitation of our study design.

4. The focus of the background is on renal function but the primary endpoint was HOMA-IR? Is it possible to explain why this is the primary endpoint?

Response: We have clarified why HOMA-IR was chosen as the primary endpoint in the methods section.

Methods, Study endpoints (page 8):
The primary study endpoint was the mean change in HOMA-IR from baseline to 5 days between groups. HOMA-IR was selected as the primary endpoint because risperidone has the potential to increase glucose levels and insulin resistance, and we first aimed to assess the metabolic safety profile of risperidone in healthy individuals.

5. How and when were fecal samples collected for microbiome analysis?

Response: We have added detailed information about fecal sample collection procedures in the methods section.

Methods, Laboratory measurements (page 7):
Intestinal bacterial flora analysis was conducted based on 16S ribosomal ribonucleic acid (rRNA) sequencing of fecal samples using QIIME2 (version 2021.2) by Takara Bio’s Biomedical Center (Shiga, Japan). Fecal samples were collected by participants at home using a collection kit (FS-0017, TechnoSuruga Lab. Co., Ltd., Shizuoka, Japan) at baseline and on day 5.

Powered by Editorial Manager® and ProduXion Manager® from Aries Systems Corporation

6. Did between group comparisons of mean changes correct for baseline values?

Response: We did not adjust for baseline values in our between-group comparisons. The baseline characteristics were generally comparable between the two groups, which reduced the necessity for baseline adjustment.

7. For the demographic/baseline comparisons between the groups, can you put statistical analyses to these statements of being comparable and the BMI "likely" being different? Were any identified differences controlled for in a sensitivity analysis to assess for potential effects on primary
and secondary outcomes. Perhaps this should be included in the statistics of table 1?

Response: We have added statistical analyses to the demographic comparisons in Table 1 as mentioned in response to Reviewer 1, comment 1.

8. Table 1 footnote should explain what data is (%, means, s.d., etc).

Response: We have expanded the Table 1 footnote to explain the data presentation format.

Table 1 footnote (page 9-10):
Data are described as mean (standard deviation) for continuous variables or n (%) for categorical variables.

9. Is it possible to utilize the supplementary material to provide in-depth data of your microbiome analysis? The presentation of
microbiome results is underwhelming given the breadth of data generally obtained from microbiome analysis.

Response: We agree that the microbiome analysis presentation could be enhanced and have added more detailed microbiome results to the supplementary material.

Results, Effects of risperidone on the fecal microbiome (page 12):
…. Phylum-level analysis revealed that both groups were dominated by Firmicutes and Bacteroidetes, accounting for over 80% of the microbiome composition at baseline and no significant changes were observed in the composition at day 5 in either group (S2 Fig and S3 Table).

10. The discussion is lacking in depth and detail, especially as it regards the microbiome analysis, why your renal findings might contradict that
of other studies.

Response: We have substantially expanded the discussion section to provide more depth and detail, particularly regarding microbiome analysis and potential mechanisms explaining our findings.

Discussion (page 13-14):
…. Although fecal d-serine levels were not measured in the present study, we aimed to minimize confounding mediated dietary factors through the restriction of the intake of fermented foods, antibiotics, and lactic acid supplements during the study period. In addition, the lack of significant changes in gut microbiome diversity during the short treatment period suggests that the increases in D-serine levels were primarily due to direct enzyme inhibition rather than microbial effects. These findings ensured that the observed changes in d-serine were attributable to risperidone administration rather than the impact of dietary or gut microbiota-related factors.
….However, a population-based cohort study reported an increased risk of acute kidney injury-related hospitalizations in elderly patients treated with atypical antipsychotics, including risperidone.[16] The absence of acute adverse effects on kidney function in healthy participants in our study contrasts with some previous reports of antipsychotic-associated nephrotoxicity, which may be related to our study population (healthy individuals vs. patients with comorbidities) or the short duration of exposure. These discrepancies underscore the need for further studies to elucidate the kidney safety profile of risperidone particularly in vulnerable populations such as elderly individuals and patients with CKD.

Powered by Editorial Manager® and ProduXion Manager® from Aries Systems Corporation

Additional Information:	
**Question**	**Response**
**Financial Disclosure**<br><br>Enter a financial disclosure statement that describes the sources of funding for the work included in this submission. Review the submission guidelines for detailed requirements. View published research articles from *PLOS ONE* for specific examples.<br><br>This statement is required for submission and **will appear in the published article** if the submission is accepted. Please make sure it is accurate.<br><br>**Funded studies**<br>Enter a statement with the following details:<br>• Initials of the authors who received each award<br>• Grant numbers awarded to each author<br>• The full name of each funder<br>• URL of each funder website<br>• Did the sponsors or funders play any role in the study design, data collection and analysis, decision to publish, or preparation of the manuscript?<br><br>Did you receive funding for this work?	Yes
Please add funding details.<br>as follow-up to "**Financial Disclosure**<br><br>Enter a financial disclosure statement that describes the sources of funding for the work included in this submission. Review the submission guidelines for detailed requirements. View published research articles from *PLOS ONE* for specific examples.<br><br>This statement is required for submission and **will appear in the published article** if the submission is accepted. Please make sure it is accurate.	This study was supported by KAGAMI INC.. MM is the founder and CEO of KAGAMI INC., a startup company working on chiral amino acid analysis and research for medical applications. The funder provided blinded amino acid level measurements, but had no role in study design, data collection and analysis. MO is supported by a grant from the 2021 public offering research on clinical research by Kanazawa University Hospital and Initiative for Realizing Diversity in the Research Environment, MEXT. Other authors did not receive any specific grant from funding agencies in the public, commercial, or not-for-profit sectors.

**Funded studies**<br>Enter a statement with the following details:<br>• Initials of the authors who received each award<br>• Grant numbers awarded to each author<br>• The full name of each funder<br>• URL of each funder website<br>• Did the sponsors or funders play any role in the study design, data collection and analysis, decision to publish, or preparation of the manuscript?<br><br>Did you receive funding for this work?"	
Please select the country of your main research funder (please select carefully as in some cases this is used in fee calculation).<br>as follow-up to "**Financial Disclosure**<br><br>Enter a financial disclosure statement that describes the sources of funding for the work included in this submission. Review the submission guidelines for detailed requirements. View published research articles from *PLOS ONE* for specific examples.<br><br>This statement is required for submission and **will appear in the published article** if the submission is accepted. Please make sure it is accurate.<br><br>**Funded studies**<br>Enter a statement with the following details:<br>• Initials of the authors who received each award<br>• Grant numbers awarded to each author<br>• The full name of each funder<br>• URL of each funder website<br>• Did the sponsors or funders play any role in the study design, data collection and analysis, decision to publish, or preparation of the manuscript?<br><br>Did you receive funding for this work?"	JAPAN - JP
**Competing Interests**	MM is the founder and CEO of KAGAMI INC. This does not alter our adherence to

Use the instructions below to enter a competing interest statement for this submission. On behalf of all authors, disclose any competing interests that could be perceived to bias this work—acknowledging all financial support and any other relevant financial or non-financial competing interests.<br><br>This statement is required for submission and **will appear in the published article** if the submission is accepted. Please make sure it is accurate and that any funding sources listed in your Funding Information later in the submission form are also declared in your Financial Disclosure statement.<br><br>View published research articles from *PLOS ONE* for specific examples.<br><br>**NO authors have competing interests**<br><br>Enter: *The authors have declared that no competing interests exist.*<br><br>**Authors with competing interests**<br><br>Enter competing interest details beginning with this statement:<br><br>*I have read the journal's policy and the authors of this manuscript have the following competing interests: [insert competing interests here]*<br><br>* typeset	PLOS ONE policies on sharing data and materials.
**Ethics Statement**<br><br>Enter an ethics statement for this submission. This statement is required if the study involved:<br><br>• Human participants<br>• Human specimens or tissue<br>• Vertebrate animals or cephalopods<br>• Vertebrate embryos or tissues	The study protocol was approved by the Medical Ethics Committee of Kanazawa University (approval no: 2021-004) and was registered at jRCT (jRCTs041210165).). The study was conducted in accordance with the the Declaration of Helsinki. Written informed consent was obtained from all participants prior to enrollment.

• Field research<br><br>Write "N/A" if the submission does not require an ethics statement.<br><br>General guidance is provided below. Consult the submission guidelines for detailed instructions. **Make sure that all information entered here is included in the Methods section of the manuscript.**	

Powered by Editorial Manager® and ProduXion Manager® from Aries Systems Corporation

**Format for specific study types**<br><br>**Human Subject Research (involving human participants and/or tissue)**<br>• Give the name of the institutional review board or ethics committee that approved the study<br>• Include the approval number and/or a statement indicating approval of this research<br>• Indicate the form of consent obtained (written/oral) or the reason that consent was not obtained (e.g. the data were analyzed anonymously)<br><br>**Animal Research (involving vertebrate animals, embryos or tissues)**<br>• Provide the name of the Institutional Animal Care and Use Committee (IACUC) or other relevant ethics board that reviewed the study protocol, and indicate whether they approved this research or granted a formal waiver of ethical approval<br>• Include an approval number if one was obtained<br>• If the study involved *non-human primates*, add *additional details* about animal welfare and steps taken to ameliorate suffering<br>• If anesthesia, euthanasia, or any kind of animal sacrifice is part of the study, include briefly which substances and/or methods were applied<br><br>**Field Research**<br><br>Include the following details if this study involves the collection of plant, animal, or other materials from a natural setting:<br>• Field permit number<br>• Name of the institution or relevant body that granted permission	
**Data Availability**<br><br>Authors are required to make all data underlying the findings described fully available, without restriction, and from the time of publication. PLOS allows rare exceptions to address legal and ethical concerns. See the PLOS Data Policy and FAQ for detailed information.	Yes - all data are fully available without restriction

A Data Availability Statement describing where the data can be found is required at submission. Your answers to this question constitute the Data Availability Statement and **will be published in the article**, if accepted.<br><br>**Important:** Stating 'data available on request from the author' is not sufficient. If your data are only available upon request, select 'No' for the first question and explain your exceptional situation in the text box.<br><br>Do the authors confirm that all data underlying the findings described in their manuscript are fully available without restriction?	
**Describe where the data may be found in full sentences. If you are copying our sample text, replace any instances of XXX with the appropriate details.**<br><br>• If the data are **held or will be held in a public repository**, include URLs, accession numbers or DOIs. If this information will only be available after acceptance, indicate this by ticking the box below. For example: *All XXX files are available from the XXX database (accession number(s) XXX, XXX.)*.<br>• If the data are all contained **within the manuscript and/or Supporting Information files**, enter the following: *All relevant data are within the manuscript and its Supporting Information files.*<br>• If neither of these applies but you are able to provide **details of access elsewhere**, with or without limitations, please do so. For example:<br><br>*Data cannot be shared publicly because of [XXX]. Data are available from the XXX Institutional Data Access / Ethics Committee (contact via XXX) for researchers who meet the criteria for access to confidential data.*<br><br>*The data underlying the results presented in the study are available from (include the name of the third party*	The dataset of this study is available from the Figshare repository at the following DOI: https://doi.org/10.6084/m9.figshare.30000049.v1.

and contact information or URL).<br>• This text is appropriate if the data are owned by a third party and authors do not have permission to share the data.<br><br>* typeset	
Additional data availability information:	

Manuscript

Click here to access/download;Manuscript;Risperidone_pilot_PLOSone_2025

Title page

Original Research

Effects of risperidone on amino acid metabolism, glucose, and kidney function in healthy adults: A pilot randomized controlled trial

Short title

Effects of risperidone on amino acid

Authors

Megumi Oshima[1], Tadashi Toyama[1,2], Yusuke Nakade[3], Sakae Miyagi[4], Hisayuki Ogura[1], Shiori Nakagawa[1], Takahiro Yuasa[1], Akihiko Koshino[1], Keisuke Horikoshi[1], Taichiro Minami[1], Keisuke Sako[1], Shunsuke Tsuge[1], Akira Tamai[1], Ryo Nishioka[1], Taro Miyagawa[1], Kiyoaki Ito[1], Shinji Kitajima[1], Ichiro Mizushima[1], Akinori Hara[1], Norihiko Sakai[1], Miho Shimizu[1], Toshiaki Tokumaru[5], Makoto Tsubomoto[6], Mitsuru Kikuchi[6,7], Masashi Kinoshita[8], Mitsutoshi Nakada[8], Masashi Mita[9], Yasunori Iwata[1*], Takashi Wada[1]

Affiliations

[1] Department of Nephrology and Rheumatology, Kanazawa University, Kanazawa, Japan

[2] Department of Nephrology, Faculty of Medical Sciences, University of Fukui, Fukui, Japan

[3] Department of Clinical Laboratory, Kanazawa University, Kanazawa, Japan.

[4] Innovative Clinical Research Center, Kanazawa University, Kanazawa, Japan

[5] Department of Nutrition, Kanazawa University Hospital, Kanazawa, Japan

[6] Department of Psychiatry and Behavioral Science, Kanazawa University Graduate School of Medical Sciences, Kanazawa, Japan.

[7] Research Center for Child Mental Development, Kanazawa University, Kanazawa, Japan.

[8] Department of Neurosurgery, Kanazawa University, Kanazawa, Japan.

[9] KAGAMI INC., 7-7-15, Saito-Asagi, Ibaraki, Osaka, Japan

***Corresponding author**

Yasunori Iwata

13-1 Takara-machi, Kanazawa, Ishikawa, Japan 920-8641

Tel: +81-76-265-2499

Email: iwatay@staff.kanazawa-u.ac.jp

Word count

Word count abstract: 199 words (limit 200 words)

Word count manuscript: 2531 words (limit 6000 words)

Table and Figure: 3 Figures and 3 Tables

Supplemental Material: 2 Figures and 2 Table

Abbreviations

CKD: chronic kidney disease

eGFR: estimated glomerular filtration rate

HOMA-IR: homeostasis model assessment of insulin resistance

UACR: urine albumin to creatinine ratio

rRNA: ribosomal ribonucleic acid

Abstract

D-serine administration prevents kidney damage in murine models of acute kidney injury, and risperidone inhibits the activity of D-amino acid oxidase, which regulate plasma D-amino acid levels. This pilot randomized controlled trial investigated the effects of risperidone on glucose, amino acid metabolism, and kidney function in healthy adults. Healthy adults with a homeostasis model assessment of insulin resistance (HOMA-IR) of ≥ 1.6 and estimated glomerular filtration rate (eGFR) of ≥ 60 mL/min/1.73m^2 were randomly assigned to the risperidone and control groups. The risperidone group received 0.5 mg/day risperidone for 4 days. The primary outcome was mean change in HOMA-IR on day 5, and the secondary outcomes were changes in D-amino acid levels, eGFR, and urinary albumin. Seven participants were randomized to the risperidone and control groups. The changes in HOMA-IR, eGFR, and urinary albumin on day 5 were not significantly different between the two groups (all $p>0.05$). Mean changes in plasma D-serine level and urinary D-serine/creatinine ratio were significantly higher in the risperidone group than in the control group (0.2 vs. −0.3 nmol/mL, $p=0.03$ and 38.2 vs. −25.8 nmol/mL, $p=0.01$, respectively). Short-term risperidone affects D-serine metabolism without instigating acute adverse effects on kidney or glucose homeostasis in healthy individuals.

Clinical Trial Registry number: This study was registered with the Japan Registry for Clinical Trials (jRCTs041210165).

Introduction

Chronic kidney disease (CKD), which impacts more than 10% of the global population, is associated with severe outcomes, including end-stage kidney disease and cardiovascular complications.[1] Despite the demonstrated efficacy of several therapeutic agents, such as renin-angiotensin system inhibitors and sodium-glucose cotransporter-2 inhibitors, in slowing CKD progression, the residual risk for disease progression remains significant.[2, 3] Thus, the development of novel therapeutic approaches are urgently needed to mitigate the risk and progression of CKD in the general population.

The renoprotective roles of D-amino acids, such as D-serine and D-alanine, have been demonstrated in several recent in vitro and in vivo studies.[4-6] For example, the oral administration of D-serine and D-alanine alleviated kidney damage in a murine model of acute kidney injury induced by ischemia/reperfusion,[4, 5] whereas elevated D-serine levels were associated with tissue remodeling in a nephrectomy model.[6] Clinical studies have also found that the plasma D-serine and D-alanine concentrations are higher in patients with CKD than in healthy individuals,[7-9] suggesting their potential as biomarkers and therapeutic targets in kidney diseases.

Risperidone, an atypical antipsychotic agent widely used for the treatment of schizophrenia, exerts its primary effects through the antagonism of D2 dopamine and 5-HT2A serotonin receptors.[10] Additionally, risperidone also inhibits D-amino acid oxidase, which degrades D-serine and D-alanine.[11-13] In a previous observational study of patients in routine clinical care, we reported that risperidone use was associated with a reduced risk of decline in kidney function in patients with schizophrenia,[14] raising the possibility that the effects of risperidone on D-amino acid metabolism might be a contributor to its protective effect against kidney injury.

Several studies have investigated the effects of antipsychotic medications on kidney function with mixed results. While some retrospective studies suggest that atypical antipsychotics, including risperidone, may not increase the risk of acute kidney injury compared to typical antipsychotics,[15, 16] other population-based studies have reported increased risks in elderly patients.[17] The mechanisms underlying these

effects remain unclear, but may involve direct nephrotoxic effects or indirect effects through metabolic changes.

Despite the evidence supporting the renoprotective effect of risperidone, its impact on the amino acid profile of humans remains unclear. Additionally, the interplay of risperidone with kidney function and glucose metabolism has not been well-studied in individuals without psychiatric conditions. Therefore, we conducted a pilot randomized controlled trial to investigate the impact of short-term risperidone administration on the amino acid profile, kidney function, and glucose metabolism in healthy adults. Microbiome analysis was included because gut microbiome can metabolize D-amino acids, and we aimed to control for potential confounding effects of microbial D-amino acid metabolizm on our study endpoints. Our findings offer insights into the pharmacological effects of risperidone on D-serine and D-alanine levels, laying the foundation for novel therapeutic strategies targeting D-amino acid in patients with CKD.

Materials and Methods

Study design and participants

This pilot, open-label, randomized controlled trial evaluated the efficacy of administration of risperidone for 4 days in healthy volunteers. The study protocol (S1 Protocol and S1 CONSORT Checklist) was approved by the Medical Ethics Committee of Kanazawa University (approval no: 2021-004) and was registered at jRCT (jRCTs041210165). The study was conducted in accordance with the the Declaration of Helsinki. The participants were recruited between March 22, 2022 and July 20, 2023. Written informed consent was obtained from all participants prior to enrollment.

The study enrolled healthy adults aged 20–65 years who met the following criteria: 1) no history of psychiatric or neurologic disorders, 2) fasting blood glucose levels < 140 mg/dL, 3) homeostasis model assessment of insulin resistance (HOMA-IR) ≥ 1.6, 4) estimated glomerular filtration rate (eGFR) ≥ 60 mL/min/1.73 m^2, 5) both aspartate and alanine transaminase levels < 30 IU/L, 6) no history of arrhythmias or congenital long QT syndrome, 7) no history of cancer within the past five years, and 8) presence of bowel movements at least once every four days, regardless of laxative use.

The glucose cutoff was chosen to exclude participants with overt diabetes while including those with potential insulin resistance, as measured by HOMA-IR.

Participants fulfilling the following criteria were excluded from the study: 1) any medically diagnosed condition currently under treatment, 2) history of hypersensitivity to risperidone, paliperidone, or their components, 3) pregnancy or breastfeeding, 4) participation in another clinical study within the last 3 months prior to the initiation of risperidone administration, 5) deemed unsuitable for study participation for any reason by the principal or sub-investigator, and 6) currently holding a faculty or staff position of the department in which the principal investigator is affiliated.

Eligible participants were randomized at a 1:1 ratio to the risperidone and control groups within 30 days of the study enrollment (S1 Fig). Randomization was centrally performed in the registration center using minimization, with age and sex as allocation adjustment factors. A sample size estimation was not performed due to the absence of prior data on effect size. This pilot study was designed to generate preliminary data for future power calculations.

Interventions

Participants in the risperidone group were administered 0.5 mg risperidone (Risperdal® OD, 0.5 mg) once daily at bedtime for 4 days. The lowest available dosage of 0.5 mg/day was chosen to prioritize participant safety. The 4-day treatment duration was chosen based on pharmacokinetic studies showing that steady-state levels are achieved within 3-4 days, allowing us to observe steady-state effects while minimizing exposure in healthy volunteers. To ensure compliance, participants in the risperidone group were instructed to return any remaining tablets at the end of the study for collection.

The study was conducted in an outpatient setting, and no participants was hospitalized during the intervention. Regardless of the group assignment, all participants visited the study site on days 1, 5, and 9 after study initiation. To minimize interference with the gut microbiota, participants were asked to restrict the consumption of foods containing probiotics, such as yogurt, fermented beverages, natto, miso, pickles, and kimchi, starting one week before the study initiation and continuing throughout the follow-up period. Participants were also restricted from using

antimicrobials and *Lactobacillus* preparations. Furthermore, participants were advised to avoid activities requiring alertness, such as driving or operating hazardous machinery, and to abstain from alcohol to reduce the risk of adverse events during the risperidone administration period.

Laboratory measurements

Participants underwent blood tests after fasting or at least 6 hours after meals and early morning urine tests at baseline and on days 5 and 9 after study initiation. Blood parameters that were assessed included leukocytes, erythrocytes, and platelet counts; hemoglobin; hematocrit; total protein; serum albumin; aspartate and alanine aminotransferases; blood urea nitrogen; creatinine; uric acid; sodium; potassium; calcium; inorganic phosphorus; fasting blood glucose; hemoglobin A1c; insulin; C-peptide; total cholesterol; high-density lipoprotein cholesterol; and triglycerides.

The following equation proposed by the Japanese Society of Nephrology was used to calculate eGFR: $\text{eGFR (mL/min/1.73 m}^2\text{)} = 194 \times \text{serum creatinine (mg/dL)}^{-1.094} \times \text{age (years)}^{-0.287} \times 0.739 \text{ (if female)}$.[18] The urine albumin to creatinine ratio (UACR) was also assessed. All blood and urinary parameters were measured by an independent laboratory at BML Inc. (Tokyo, Japan) using automated clinical testing technology. The results of all urinary parameters were normalized to urine creatinine to correct for differences in concentrations uniquely related to the hydration status or the urine volume of the participant.[19]

Plasma and urinary levels of DL-serine and alanine at baseline and on day 5 were determined using a two-dimensional high-performance liquid chromatography system (DASH 27B3X00322000001) by KAGAMI INC. (Osaka, Japan), according to a previously described protocol with modifications.[4, 5, 8]

Intestinal bacterial flora analysis was conducted based on 16S ribosomal ribonucleic acid (rRNA) sequencing of fecal samples using QIIME2 (version 2021.2) by Takara Bio's Biomedical Center (Shiga, Japan). Fecal samples were collected by participants at home using a collection kit (FS-0017, TechnoSuruga Lab. Co., Ltd., Shizuoka, Japan) at baseline and on day 5. Microbiome analysis included alpha diversity, such as number of operational taxonomic units, Chao-1 index, and Shannon

index, and beta diversity, such as weighted Unifrac and Bray-Curtis distances, and relative abundance, with a sampling depth of 10,000 sequences.

Study endpoints

The primary study endpoint was the mean change in HOMA-IR from baseline to 5 days between groups. HOMA-IR was selected as the primary endpoint because risperidone has the potential to increase glucose levels and insulin resistance,[20, 21] and we first aimed to assess the metabolic safety profile of risperidone in healthy individuals. Intention-to-treat analyses were performed according to the randomly assigned groups. The secondrary endpoints included the mean changes in the following parameters from baseline to 5 days between groups: plasma and urine DL-serine and DL-alanine levels, eGFR, blood pressure, body mass index, blood glucose, hemoglobin A1c, insulin, C-peptide, and UACR. Safety was monitored by assessing adverse events, medical interviews, and the review of laboratory values during the study period.

Statistical analysis

For baseline participant characteristics, continuous variables were presented as means with standard deviations and categorical variables were presented as numbers with percentages. For the primary and secondary endpoints, Student's *t*-test was used to compare mean changes in clinical and laboratory parameters from baseline to days 5 and 9 between the risperidone and control groups. Given the small sample size, Mann-Whitney U test was also performed as a non-parametric analysis to complement the parametric tests. All analyses were performed using Stata version 18 (Stata Corp, College Station, TX). A two-sided p value of <0.05 was considered statistically significan[illegible]

Results

Baseline characteristics of the participants

Between March and November 2022, a total of 8 participants were randomized to the risperidone and control groups (n = 4 participants per group), 1 participant declined to complete the study, and 7 completed the protocol treatment (Fig 1). In the risperidone and control groups, the mean participant age was 35 and 29 years, 1 (33%) and 2 (50%) of the participants were male, the mean HOMA-IR was 2.0 and 1.6, and the

mean eGFR was 90 and 92 mL/min/1.73 m^2, respectively (Table 1). The baseline participant characteristics were comparable between the two groups (all $p > 0.10$), except for body mass index, which was likely to be higher in the risperidone group than in the control group ($p = 0.03$).

Fig 1. Flowchart of the study

Table 1. Baseline participant characteristics in the risperidone and control groups

	**Risperidone (n = 3)**	**Control (n = 4)**	**p**
Age (years)	35 (12)	29 (8)	0.49
Male	1 (33)	2 (50)	0.66
Current smoking	0 (0)	0 (0)	–
Drinking habit	1 (33)	1 (25)	0.81
Body weight (kg)	66 (9)	61 (7)	0.41
Body mass index (kg/m^2)	23.8 (1.4)	21.2 (0.8)	0.03
Systolic blood pressure (mmHg)	105 (6)	113 (16)	0.45
Diastolic blood pressure (mmHg)	71 (9)	74 (5)	0.69
Aspartate aminotransferase (U/L)	16 (4)	19 (6)	0.62
Alanine aminotransferase (U/L)	12 (6)	16 (7)	0.50
eGFR (mL/min/1.73 m^2)	90 (20)	92 (13)	0.89
Uric acid (mg/dL)	4.6 (1.0)	6.3 (2.0)	0.25
Fasting blood glucose (mg/dL)	92 (9)	90 (4)	0.67
Hemoglobin A1c (%)	4.9 (0.1)	5.0 (0.2)	0.61
Insulin (μU/mL)	8.9 (2.0)	7.3 (2.9)	0.44
C peptide (ng/mL)	1.9 (0.4)	1.6 (0.5)	0.33
HOMA-IR	2.0 (0.4)	1.6 (0.6)	0.36
HOMA-β (%)	119 (57)	100 (43)	0.64
Total cholesterol (mg/dL)	227 (35)	183 (23)	0.10
HDL-cholesterol (mg/dL)	66 (21)	67 (16)	0.97
Triglycerides (mg/dL)	82 (19)	73 (23)	0.60
Urine albumin/Cr (mg/g Cr)	2.9 (1.1)	4.7 (2.9)	0.38
**Plasma amino acid**			
D-serine (nmol/mL)	1.8 (0.3)	1.9 (0.1)	0.52
L-serine (nmol/mL)	120.4 (18.4)	133.3 (17.8)	0.39
D/L-serine (%)	1.5 (0.5)	1.4 (0.2)	0.77
D-alanine (nmol/mL)	1.0 (0.2)	0.8 (0.7)	0.75
L-alanine (nmol/mL)	354.3 (50.4)	288.5 (59.8)	0.19
D/L-alanine (%)	0.3 (0.1)	0.3 (0.2)	0.96
**Urine amino acid**			
D-serine/Cr (mmol/g Cr)	123.4 (10.0)	136.6 (18.3)	0.31
L-serine/Cr (mmol/g Cr)	118.9 (53.3)	256.6 (252.1)	0.40

D-alanine/Cr (mmol/g Cr)	27.1 (9.5)	23.7 (19.6)	0.80
L-alanine/Cr (mmol/g Cr)	142.3 (75.5)	113.7 (46.3)	0.56

Abbreviations; eGFR, estimated glomerular filtration rate; HOMA-IR, Homeostatic model assessment of insulin resistance; HOMA-β, Homeostatic model assessment of beta cell function; HDL, high density lipoprotein; Cr, creatinine.
Data are described as mean (standard deviation) for continuous variables or n (%) for categorical variables. Statistical comparisons were performed using Student's *t*-test for continuous variables and Fisher's exact test for categorical variables.

Comparison of changes in DL-amino acid levels

At baseline, the mean (± SD) plasma D-serine levels were 1.8 ± 0.3 nmol/mL and 1.9 ± 0.1 nmol/mL in the risperidone and control groups, respectively. The mean plasma D-serine level increased by 0.24 ± 0.36 nmol/mL on day 5 compared to the baseline in the risperidone group and decreased by −0.32 ± 0.13 nmol/mL during the same time period in the control group (Fig 2A and S1 Table). The mean plasma D-serine level was 0.56 (95% CI 0.07–1.05) nmol/mL higher in the risperidone group than in the control group on day 5 after the study initiation (p = 0.03). Similarly, the mean change in urinary D-serine-to-creatinine ratio was significantly higher in the risperidone group than in the control group (38.2 ± 19.8 and −25.8 ± 23.4 mmol/g Cr; respectively; between-group difference: 64.0 mmol/g Cr, 95% CI 20.8–107.2; p = 0.01) compared to the control group. The significant differences in plasma D-serine levels and urinary D-serine-to-creatinine ratio between groups remained consistent when analyzed using Mann-Whitney U tests (S1 Table).

Fig 2. Mean plasma and urine DL-serine (A) and DL-alanine (B) levels during follow-up in the risperidone and control groups

The mean changes in plasma D-alanine level and urinary D-alanine-to-creatinine ratio were numerically higher in the risperidone group than in the control group, with no statistical difference (0.54 vs. −0.31 nmol/mL, p = 0.25 and 24.98 vs. −9.42 nmol/mL, p = 0.20, respectively) (Fig 2B and S1 Table). Conversely, no significant changes were observed in plasma and urinary L-serine and L-alanine levels during the follow-up period and these parameters did not significantly differ between the two groups (Fig 2 and S1 Table).

Effects of risperidone on clinical and laboratory parameters

The mean change in HOMA-IR was not statistically significantly different between the risperidone and control groups during the treatment period (0.88 ± 0.58 and 0.42 ± 1.00, p = 0.51) (Fig 3 and Table 2). The change in mean blood glucose from the baseline to day 5 after study initiation was marginally higher in the risperidone group than in the control group by Student's t-test (4.0 ± 2.0 vs. −1.3 ± 3.0 mg/dL, respectively; p = 0.048), however this difference was not significant when analyzed using the Mann-Whitney U test (p = 0.07). The mean change in eGFR did not differ between the risperidone and control groups (1.0 ± 8.7 and −0.4 ± 15.6 mL/min/1.73 m^2, respectively; p = 0.90). The changes in mean blood pressure, body mass index, hemoglobin A1c, insulin, C-peptide, and UACR were not statistically significantly different between the two groups (Table 2). All other parameters except fasting blood glucose showed consistent results between parametric and non-parametric analyses.

Fig 3. Mean homeostatic model assessment of insulin resistance (HOMA-IR) (A), fasting blood glucose (B), and estimated glomerular filtration rate (eGFR) (C) levels during follow-up in the risperidone and control groups

Table 2. Mean changes in clinical and laboratory parameters in the risperidone and control groups

		Mean (SD) change from baseline			
	Day	Risperidone (n = 3)	Control (n = 4)	p*	p**
HOMA-IR	5	0.88 (0.58)	0.42 (1.0)	0.51	0.29
	9	0.03 (1.25)	0.15 (0.88)	0.88	1.00
Systolic blood pressure (mmHg)	5	7.7 (18.0)	–3.3 (7.7)	0.32	0.29
	9	8.0 (19.5)	1.8 (15.3)	0.65	0.48
Diastolic blood pressure (mmHg)	5	–2.3 (13.7)	–3.5 (2.5)	0.87	0.48
	9	1.0 (8.9)	–2.3 (8.7)	0.65	0.48
Body mass index (kg/m^2)	5	0.1 (0.2)	–0.2 (0.2)	0.10	0.08
	9	–0.03 (0.2)	–0.06 (0.2)	0.87	1.00
eGFR (mL/min/1.73 m^2)	5	1.0 (8.7)	–0.4 (15.6)	0.90	0.72
	9	1.8 (10.7)	0.02 (2.8)	0.76	0.72
Fasting blood glucose (mg/dL)	5	4.0 (2.0)	–1.3 (3.0)	0.048	0.07
	9	–7.7 (4.7)	–3.0 (3.8)	0.21	0.21
Hemoglobin A1c (%)	5	0.00 (0.10)	0.08 (0.05)	0.24	0.29
	9	0.07 (0.06)	0.05 (0.06)	0.72	1.00

Insulin (μU/mL)	5	3.2 (2.1)	1.9 (4.0)	0.62	0.48
	9	1.2 (5.8)	0.9 (3.8)	0.95	1.00
C peptide (ng/mL)	5	0.90 (0.95)	0.20 (0.59)	0.28	0.16
	9	0.03 (0.67)	0.03 (0.59)	0.99	0.86
Urine albumin/Cr (mg/g Cr)	5	–0.1 (1.0)	–0.3 (2.4)	0.92	0.72
	9	–0.4 (1.1)	–1.4 (2.2)	0.50	0.48

Abbreviations; HOMA-IR, Homeostatic model assessment of insulin resistance; eGFR, estimated glomerular filtration rate; Cr, creatinine. P* values were calculated using Student's t-test and p** values were calculated using Mann-Whitney U test as non-parametric analysis.

Exploratory analysis to assess the effects of risperidone on additional clinical and laboratory parameters (S2 Table) revealed no significant changes in body weight, HOMA for β-cell function, aspartate and alanine aminotransferase, uric acid, total and high-density lipoprotein cholesterol, and triglycerides after 4 days of risperidone administration.

Effects of risperidone on the fecal microbiome

In the 16S rRNA analysis, no significant differences were observed in alpha diversity (species richness) metrics of the fecal microbiome on day 5 compared to the baseline in both risperidone and control group (paired *t*-test, $p > 0.10$) (S2 Fig). For beta diversity (overall structural similarity and variation), the weighted Unifrac distances were visually unchanged, and the Gray-Curtis distances were similar ($p = 0.81$) between the risperidone and control groups during the treatment period (S2 Fig). Phylum-level analysis revealed that both groups were dominated by Firmicutes and Bacteroidetes, accounting for over 80% of the microbiome composition at baseline and no significant changes were observed in the composition at day 5 in either group (S2 Fig and S3 Table).

Safety

All adverse events observed during the study are presented in Table 3. All participants reported a total of eight non-serious adverse events. In the risperidone group, two participants experienced somnolence, and one participant experienced abdominal distension. One participant in each group reported blood pressure of > 140/90 mmHg. No participant in the risperidone group reported hypotension or dizziness, and no serious adverse events were recorded.

Table 3. Safety in the risperidone and control groups

	N (%) Risperidone (n = 3)	Control (n = 4)
Somnolence	2 (67)	0 (0)
Hypertension	1 (33)	1 (25)
Abdominal distension	1 (33)	0 (0)
Hypertriglyceridemia	0 (0)	1 (25)
Hypercholesterolemia	0 (0)	1 (25)
Liver injury	0 (0)	1 (25)
Hypotension	0 (0)	0 (0)
Dizziness	0 (0)	0 (0)

Discussion

In this open-label, randomized pilot clinical trial, we found that the short-term administration of risperidone led to an increase in plasma and urinary D-serine levels in healthy adults with normal kidney function. Additionally, risperidone did not adversely eGFR levels or insulin resistance. Overall, these results suggest that risperidone impacts D-serine metabolism without an acute adverse impact on kidney function or glucose homeostasis in healthy individuals.

The significant elevation in D-serine levels observed immediately after risperidone administration is a notable finding, which is likely attributable to risperidone-mediated inhibition of D-amino acid oxidase, preventing D-serine degradation.[11, 22] The contribution of dietary and microbial factors to D-serine levels should also be considered. D-serine is present in various food sources, including fermented products rich in D-amino acids due to microbial fermentation by serine racemase.[23-26] Although fecal D-serine levels were not measured in the present study, we aimed to minimize confounding mediated dietary factors through the restriction of the intake of fermented foods, antibiotics, and lactic acid supplements during the study period. In addition, the lack of significant changes in gut microbiome diversity during the short treatment period suggests that the increases in D-serine levels were primarily due to direct enzyme inhibition rather than microbial effects. These findings ensured that the observed changes in D-serine were attributable to risperidone administration rather than the impact of dietary or gut microbiota-related factors.

Given the small sample size of this pilot study, the magnitude of observed effects may be more informative than statistical significance. The substantial increase in plasma D-serine levels (0.56 nmol/mL difference, 95% CI 0.07–1.05) and urinary D-serine-to-creatinine ratio (64.0 mmol/g Cr difference, 95% CI 20.8–107.2) represent clinically meaningful changes that warrant further investigation in larger studies. Similarly, the numerical increase observed in plasma D-alanine levels and urinary D-alanine-to-creatinine ratio, while not reaching statistical significance, provide valuable preliminary data for effect size calculations in future trial designs.

Short-term risperidone administration did not affect kidney function in healthy individuals enrolled in our study. Few studies have investigated the efficacy and safety of risperidone on kidney function, with contradictory findings. Results from several retrospective cohort studies suggest that risperidone does not increase the risk of acute kidney injury compared to typical antipsychotics.[15, 16] In a previous retrospective observational study, we also found that risperidone use was associated with a lower risk of decline in kidney function in patients with schizophrenia.[14] However, a population-based cohort study reported an increased risk of acute kidney injury-related hospitalizations in elderly patients treated with atypical antipsychotics, including risperidone.[17] The absence of acute adverse effects on kidney function in healthy participants in our study contrasts with some previous reports of antipsychotic-associated nephrotoxicity, which may be related to our study population (healthy individuals vs. patients with comorbidities) or the short duration of exposure. These discrepancies underscore the need for further studies to elucidate the kidney safety profile of risperidone particularly in vulnerable populations such as elderly individuals and patients with CKD.

We also found a slight increase in blood glucose levels during risperidone administration. Given the high variability of glucose levels influenced by diet and lifestyle, this change might not be directly related to risperidone administration. Additionally, no significant changes were observed in other glucose metabolism markers, including HOMA-IR and insulin, indicating that risperidone might have a limited impact on glycemic control during short-term use. These results align with previous studies reporting the relatively modest effect of risperidone on glucose metabolism

compared to other atypical antipsychotics, which are known for their association with metabolic side effects.[27, 28]

This study has several limitations. First, the open-label design might have introduced performance bias due to the lack of blinding. Second, we focused exclusively on a healthy population in a pilot study with a small samle size and short follow-up period, which limited the robustness of the safety data. Any inferences should be interpreted with caution. The small sample size limits our findings and any inferences should be interpreted with caution. Third, the assumption of normality for Student's *t*-test may not be reliably verified with small sample sizes, which represents a limitation of our statistical approach. Longer-term clinical trials with larger sample sizes in patients with CKD are warranted to assess the sustained effects and safety of risperidone in this patient population. Fourth, we did not specifically control for protein intake during the study period, which may have influenced kidney parameters. Finally, the study was conducted in a Japanese population and our findings might not be generalizable to other ethnic groups with different genetic or dietary backgrounds.

Conclusion

In conclusion, short-term administration of risperidone at 0.5 mg over 4 days effectively increased plasma D-serine concentrations in healthy participants. Although a slight increase in blood glucose levels was observed, other markers of glucose metabolism and kidney function remained stable during the study period. These findings suggest that risperidone modulates D-serine metabolism without acute adverse effects on kidney function or glucose homeostasis in healthy individuals. These findings provide a foundation for future studies exploring the therapeutic applications of risperidone in kidney disease, and the potential safety and efficacy of risperidone in modulating D-serine metabolism warrant further investigation in clinical trials including patients with CKD.

Acknowledgments

The authors thank the participants who took part in the study. We would like to acknowledge Yuko Oyama, Mari Shimizu, and Eri Umeda at Kanazawa University, who

have assisted in sample collection and processing. We also thank Hiroshi Imoto, Eiichi Negishi, Maiko Nakane, and Shoto Ishigo in KAGAMI INC. who have contributed to the development of alanine powder formulation and chiral amino acid analysis.

Financial Disclosure Statement

This study was supported by KAGAMI INC.. MM is the founder and CEO of KAGAMI INC., a startup company working on chiral amino acid analysis and research for medical applications. The funder provided blinded amino acid level measurements, but had no role in study design, data collection and analysis. MO is supported by a grant from the 2021 public offering research on clinical research by Kanazawa University Hospital and Initiative for Realizing Diversity in the Research Environment, MEXT. Other authors did not receive any specific grant from funding agencies in the public, commercial, or not-for-profit sectors.

References

1. United States Renal Data System. 2023 USRDS Annual Data Report: Epidemiology of kidney disease in the United States. National Institutes of Health, National Institute of Diabetes and Digestive and Kidney Diseases, Bethesda, MD: , 2023. Report No.

2. Xie X, Liu Y, Perkovic V, Li X, Ninomiya T, Hou W, et al. Renin-Angiotensin System Inhibitors and Kidney and Cardiovascular Outcomes in Patients With CKD: A Bayesian Network Meta-analysis of Randomized Clinical Trials. Am J Kidney Dis. 2016;67(5):728-41. Epub 2015/11/26. doi: 10.1053/j.ajkd.2015.10.011. PubMed PMID: 26597926.

3. Nuffield Department of Population Health Renal Studies G, Consortium SiM-AC-RT. Impact of diabetes on the effects of sodium glucose co-transporter-2 inhibitors on kidney outcomes: collaborative meta-analysis of large placebo-controlled trials. Lancet. 2022;400(10365):1788-801. Epub 2022/11/10. doi: 10.1016/S0140-6736(22)02074-8. PubMed PMID: 36351458; PubMed Central PMCID: PMCPMC7613836.

4. Nakade Y, Iwata Y, Furuichi K, Mita M, Hamase K, Konno R, et al. Gut microbiota-derived D-serine protects against acute kidney injury. JCI Insight. 2018;3(20). Epub 2018/10/20. doi: 10.1172/jci.insight.97957. PubMed PMID: 30333299; PubMed Central PMCID: PMCPMC6237464.

5. Iwata Y, Nakade Y, Kitajima S, Yoneda-Nakagawa S, Oshima M, Sakai N, et al. Protective effect of d-alanine against acute kidney injury. Am J Physiol Renal Physiol. 2022;322(6):F667-F79. Epub 2022/04/19. doi: 10.1152/ajprenal.00198.2021. PubMed PMID: 35435002.

6. Hesaka A, Tsukamoto Y, Nada S, Kawamura M, Ichimaru N, Sakai S, et al. d-Serine Mediates Cellular Proliferation for Kidney Remodeling. Kidney360. 2021;2(10):1611-24. Epub 2022/04/05. doi: 10.34067/KID.0000832021. PubMed PMID: 35372967; PubMed Central PMCID: PMCPMC8785787.

7. Nakade Y, Iwata Y, Sakai N, Mita M, Nakane M, Hamase K, et al. Increased levels of oral Streptococcus-derived D-alanine in patients with chronic kidney disease and diabetes mellitus. Sci Rep. 2022;12(1):21773. Epub 2022/12/17. doi: 10.1038/s41598-022-26175-1. PubMed PMID: 36526888; PubMed Central PMCID: PMCPMC9758232.

8. Ishii C, Akita T, Mita M, Ide T, Hamase K. Development of an online two-dimensional high-performance liquid chromatographic system in combination with tandem mass spectrometric detection for enantiomeric analysis of free amino acids in human physiological fluid. J Chromatogr A. 2018;1570:91-8. Epub 2018/08/15. doi: 10.1016/j.chroma.2018.07.076. PubMed PMID: 30104059.

9. Iwata Y, Okushima H, Hesaka A, Kawamura M, Imamura R, Takahara S, et al. Identification of Diabetic Nephropathy in Patients Undergoing Kidney Biopsy through Blood and Urinary Profiles of d-Serine. Kidney360. 2021;2(11):1734-42. Epub 2022/04/05. doi: 10.34067/KID.0004282021. PubMed PMID: 35372995; PubMed Central PMCID: PMCPMC8785851.

10. Tandon R. Antipsychotics in the treatment of schizophrenia: an overview. J Clin Psychiatry. 2011;72 Suppl 1:4-8. Epub 2012/01/11. doi: 10.4088/JCP.10075su1.01. PubMed PMID: 22217436.

11. Shishikura M, Hakariya H, Iwasa S, Yoshio T, Ichiba H, Yorita K, et al. Evaluation of human D-amino acid oxidase inhibition by anti-psychotic drugs in vitro. Biosci Trends. 2014;8(3):149-54. Epub 2014/07/18. doi: 10.5582/bst.2014.01034. PubMed PMID: 25030849.
12. Onozato M, Nakazawa H, Hakariya H, Shishikura M, Nagashima C, Ishimaru K, et al. Effect of risperidone on plasma d-serine concentration in rats post-administered with d-serine. Life Sci. 2016;158:98-103. Epub 2016/06/30. doi: 10.1016/j.lfs.2016.06.021. PubMed PMID: 27352936.
13. Abou El-Magd RM, Park HK, Kawazoe T, Iwana S, Ono K, Chung SP, et al. The effect of risperidone on D-amino acid oxidase activity as a hypothesis for a novel mechanism of action in the treatment of schizophrenia. J Psychopharmacol. 2010;24(7):1055-67. Epub 2009/03/31. doi: 10.1177/0269881109102644. PubMed PMID: 19329549.
14. Oshima M, Toyama T, Nakade Y, Yomogida D, Yuasa T, Horikoshi K, et al. Association Between Risperidone Use and Kidney Function Decline in Patients with Schizophrenia: A Retrospective Cohort Study. Clin Ther. 2023;45(9):889-93. Epub 2023/07/25. doi: 10.1016/j.clinthera.2023.07.002. PubMed PMID: 37487866.
15. Jiang Y, McCombs JS, Park SH. A Retrospective Cohort Study of Acute Kidney Injury Risk Associated with Antipsychotics. CNS Drugs. 2017;31(4):319-26. Epub 2017/03/16. doi: 10.1007/s40263-017-0421-4. PubMed PMID: 28290080.
16. Sharon R, Lange T, Aakjaer M, Brogger Kristiansen S, Baltzer Houlind M, Andersen M. Incidence of hospital contacts with acute kidney injury after initiation of second-generation antipsychotics in older adults: a Danish population-based cohort study. Eur J Clin Pharmacol. 2022;78(8):1341-9. Epub 2022/06/01. doi: 10.1007/s00228-022-03339-6. PubMed PMID: 35639132; PubMed Central PMCID: PMCPMC9283184.
17. Hwang YJ, Dixon SN, Reiss JP, Wald R, Parikh CR, Gandhi S, et al. Atypical antipsychotic drugs and the risk for acute kidney injury and other adverse outcomes in older adults: a population-based cohort study. Ann Intern Med. 2014;161(4):242-8. Epub 2014/08/19. doi: 10.7326/M13-2796. PubMed PMID: 25133360.
18. Matsuo S, Imai E, Horio M, Yasuda Y, Tomita K, Nitta K, et al. Revised equations for estimated GFR from serum creatinine in Japan. Am J Kidney Dis. 2009;53(6):982-92. Epub 2009/04/03. doi: 10.1053/j.ajkd.2008.12.034. PubMed PMID: 19339088.
19. Bienaime F, Muorah M, Metzger M, Broeuilh M, Houiller P, Flamant M, et al. Combining robust urine biomarkers to assess chronic kidney disease progression. EBioMedicine. 2023;93:104635. Epub 2023/06/07. doi: 10.1016/j.ebiom.2023.104635. PubMed PMID: 37285616; PubMed Central PMCID: PMCPMC10279781.
20. Boyda HN, Procyshyn RM, Tse L, Yuen JWY, Honer WG, Barr AM. A comparison of the metabolic side-effects of the second-generation antipsychotic drugs risperidone and paliperidone in animal models. PLoS One. 2021;16(1):e0246211. Epub 2021/01/29. doi: 10.1371/journal.pone.0246211. PubMed PMID: 33508013; PubMed Central PMCID: PMCPMC7842964 following competing interests: WGH has received consulting fees or sat on paid advisory boards for Otsuka/Lundbeck, Newron, AlphaSights, Guidepoint, Translational Life Sciences and holds shares in Translational Life Sciences and Eli Lilly. He was additionally supported by the Jack Bell Chair in Schizophrenia. RMP has been a member of the following advisory boards in the past 3

years: Janssen, Lundbeck, and Otsuka; a member of the following speaker's bureaus in the past 3 years: Janssen, Lundbeck, and Otsuka; and received grants from the Canadian Institutes of Health Research. All other authors declare that no competing interests exist. This does not alter our adherence to PLOS ONE policies on sharing data and materials.

21. Koller EA, Cross JT, Doraiswamy PM, Schneider BS. Risperidone-associated diabetes mellitus: a pharmacovigilance study. Pharmacotherapy. 2003;23(6):735-44. Epub 2003/06/25. doi: 10.1592/phco.23.6.735.32178. PubMed PMID: 12820816.

22. Szilagyi B, Ferenczy GG, Keseru GM. Drug discovery strategies and the preclinical development of D-amino-acid oxidase inhibitors as antipsychotic therapies. Expert Opin Drug Discov. 2018;13(10):973-82. Epub 2018/09/18. doi: 10.1080/17460441.2018.1524459. PubMed PMID: 30220232.

23. Kato S, Ishihara T, Hemmi H, Kobayashi H, Yoshimura T. Alterations in D-amino acid concentrations and microbial community structures during the fermentation of red and white wines. J Biosci Bioeng. 2011;111(1):104-8. Epub 2010/09/28. doi: 10.1016/j.jbiosc.2010.08.019. PubMed PMID: 20869309.

24. Tian H, Zheng N, Li S, Zhang Y, Zhao S, Wen F, et al. Characterization of chiral amino acids from different milk origins using ultra-performance liquid chromatography coupled to ion-mobility mass spectrometry. Sci Rep. 2017;7:46289. Epub 2017/04/11. doi: 10.1038/srep46289. PubMed PMID: 28393862; PubMed Central PMCID: PMCPMC5385494.

25. Suzuki M, Shimizu-Hirota R, Mita M, Hamase K, Sasabe J. Chiral resolution of plasma amino acids reveals enantiomer-selective associations with organ functions. Amino Acids. 2022;54(3):421-32. Epub 2022/03/01. doi: 10.1007/s00726-022-03140-w. PubMed PMID: 35226151.

26. Sasabe J, Miyoshi Y, Rakoff-Nahoum S, Zhang T, Mita M, Davis BM, et al. Interplay between microbial d-amino acids and host d-amino acid oxidase modifies murine mucosal defence and gut microbiota. Nat Microbiol. 2016;1(10):16125. Epub 2016/09/28. doi: 10.1038/nmicrobiol.2016.125. PubMed PMID: 27670111; PubMed Central PMCID: PMCPMC5074547.

27. Yanik T, Kursungoz C, Sutcigil L, Ak M. Weight gain in risperidone therapy: investigation of peripheral hypothalamic neurohormone levels in psychotic patients. J Clin Psychopharmacol. 2013;33(5):608-13. Epub 2013/06/29. doi: 10.1097/JCP.0b013e318297980e. PubMed PMID: 23807284.

28. Stogios N, Smith E, Bowden S, Tran V, Asgariroozbehani R, McIntyre WB, et al. Metabolic adverse effects of off-label use of second-generation antipsychotics in the adult population: a systematic review and meta-analysis. Neuropsychopharmacology. 2022;47(3):664-72. Epub 2021/08/28. doi: 10.1038/s41386-021-01163-7. PubMed PMID: 34446830; PubMed Central PMCID: PMCPMC8782876.

Supporting information

S1 Protocol. Study protocol in Japanese and Japanese to English translation.

S1 CONSORT Checklist.

S1 Fig. Study design

S2 Fig. Alpha (A) and beta (B) diversities of the fecal microbiome in the risperidone and control groups

S1 Table. Mean changes in plasma and urine dl-serine and alanine levels in the risperidone and control groups. Abbreviations; Cr, creatinine.

S2 Table. Mean changes in clinical and laboratory parameters in the risperidone and control groups. Abbreviations; HOMA-β, Homeostatic model assessment of beta cell function; HDL, high density lipoprotein.

S3 Table. Relative abundance of the fecal microbiome during follow-up in the risperidone and control groups。

Fig 1

Click here to access/download;Figure;Fig 1.tiff

Figure 1. Flowchart of the study

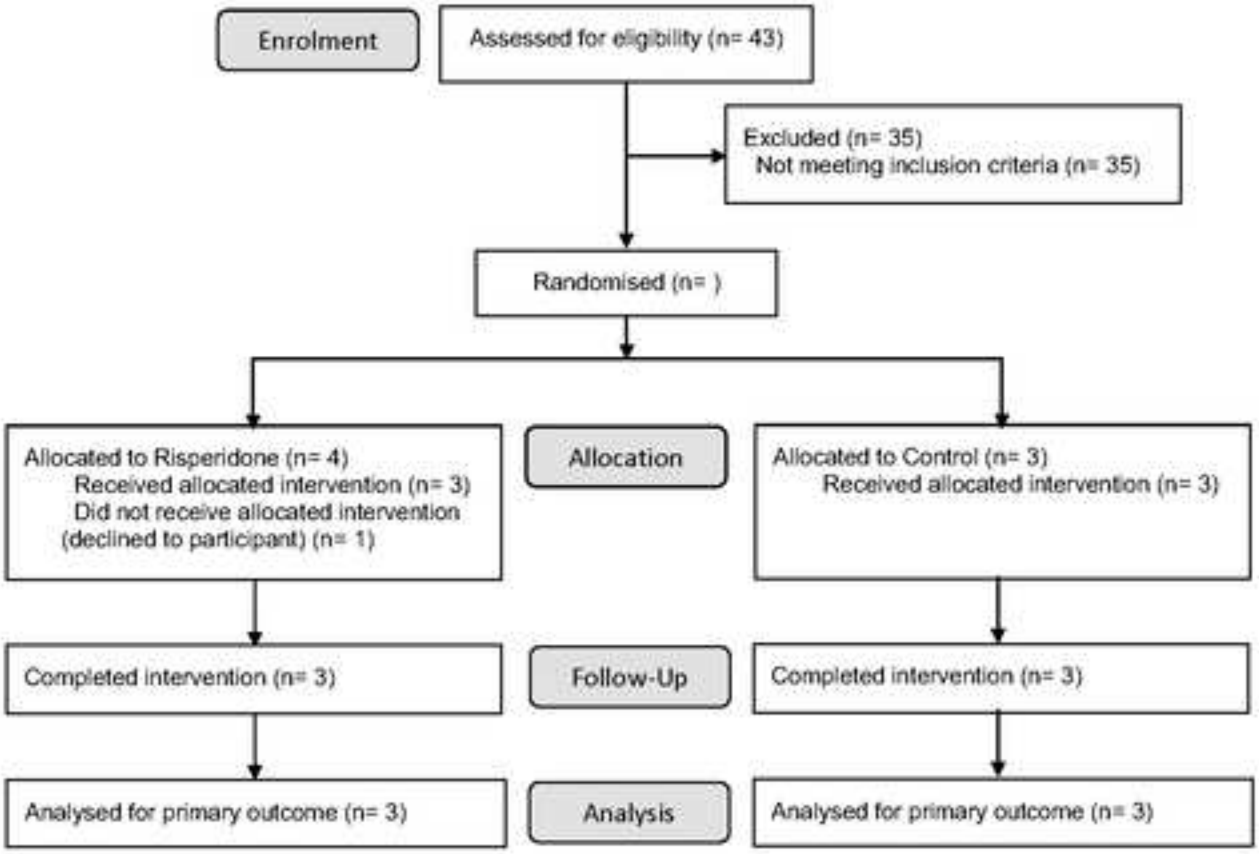

Fig 2

Click here to access/download;Figure;Fig 2.tiff

Figure 2. Mean plasma and urine DL-serine (A) and DL-alanine (B) levels during follow-up in the risperidone and control groups

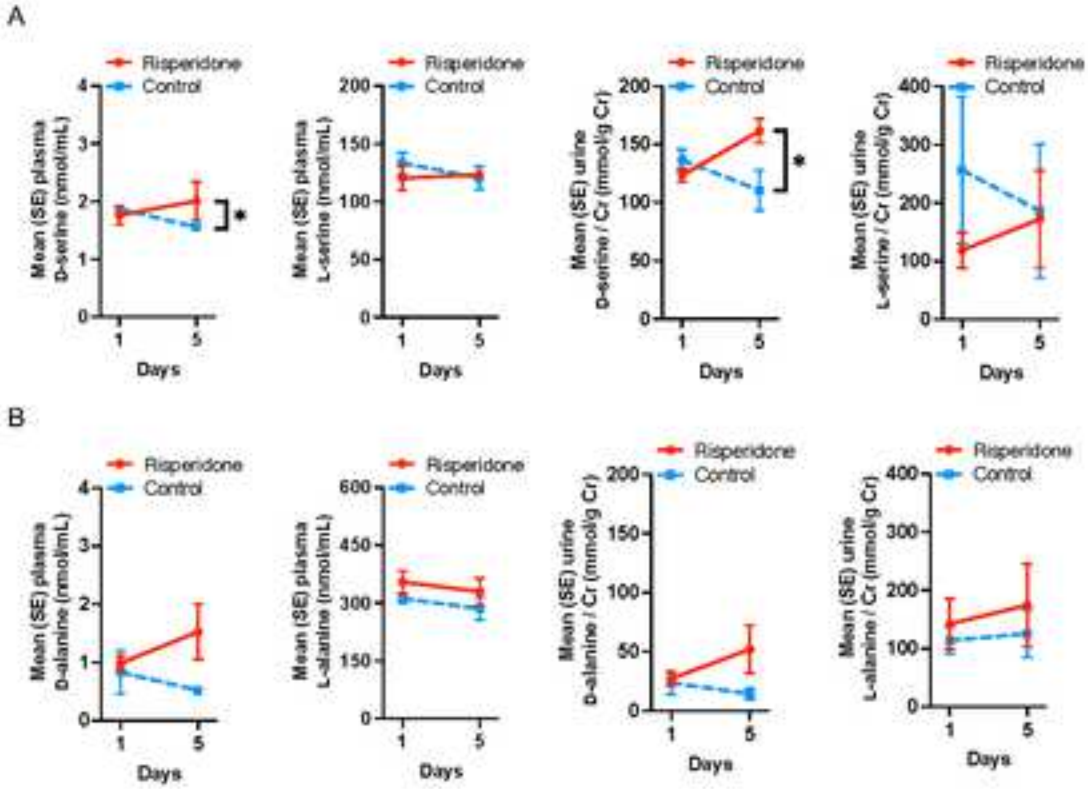

* $P < 0.05$

Fig 3

Click here to access/download;Figure;Fig 3.tiff

Figure 3. Mean homeostatic model assessment of insulin resistance (HOMA-IR) (A), fasting blood glucose (B), and estimated glomerular filtration rate (eGFR) (C) levels during follow-up in the risperidone and control groups

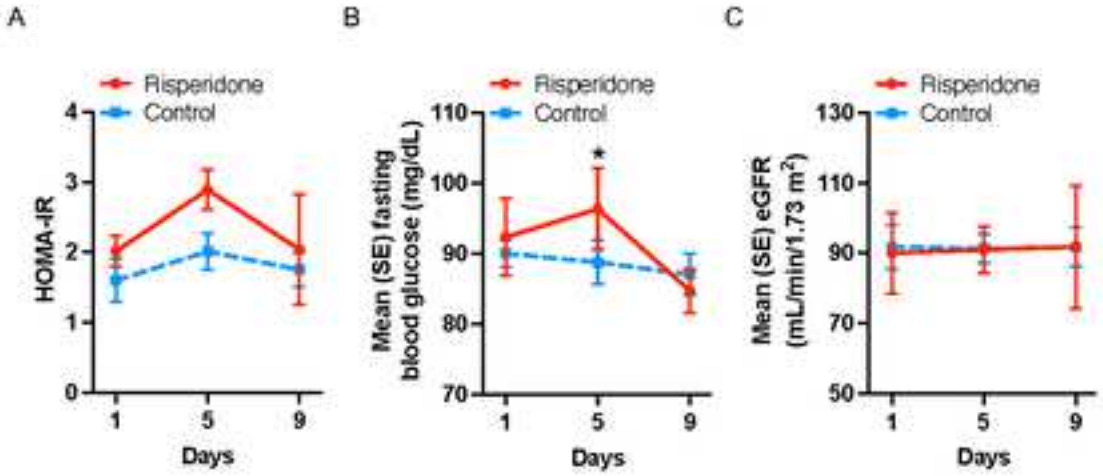

∗ P < 0.05

Click here to access/download
Supporting Information - Compressed/ZIP File Archive
Supportin information 3.zip

Approved document original

Click here to access/download
Other
Approval document_202203_japanese.pdf

Click here to access/download
Other
Approval document_202203.pdf

Title page

Original Research

Effects of risperidone on amino acid metabolism, glucose, and kidney function in healthy adults: A pilot randomized controlled trial

Short title

Effects of risperidone on amino acid

Authors

Megumi Oshima[1], Tadashi Toyama[1,2], Yusuke Nakade[3], Sakae Miyagi[4], Hisayuki Ogura[1], Shiori Nakagawa[1], Takahiro Yuasa[1], Akihiko Koshino[1], Keisuke Horikoshi[1], Taichiro Minami[1], Keisuke Sako[1], Shunsuke Tsuge[1], Akira Tamai[1], Ryo Nishioka[1], Taro Miyagawa[1], Kiyoaki Ito[1], Shinji Kitajima[1], Ichiro Mizushima[1], Akinori Hara[1], Norihiko Sakai[1], Miho Shimizu[1], Toshiaki Tokumaru[5], Makoto Tsubomoto[6], Mitsuru Kikuchi[6,7], Masashi Kinoshita[8], Mitsutoshi Nakada[8], Masashi Mita[9], Yasunori Iwata[1*], Takashi Wada[1]

Affiliations

[1] Department of Nephrology and Rheumatology, Kanazawa University, Kanazawa, Japan

[2] Department of Nephrology, Faculty of Medical Sciences, University of Fukui, Fukui, Japan

[3] Department of Clinical Laboratory, Kanazawa University, Kanazawa, Japan.

[4] Innovative Clinical Research Center, Kanazawa University, Kanazawa, Japan

[5] Department of Nutrition, Kanazawa University Hospital, Kanazawa, Japan

[6] Department of Psychiatry and Behavioral Science, Kanazawa University Graduate School of Medical Sciences, Kanazawa, Japan.

[7] Research Center for Child Mental Development, Kanazawa University, Kanazawa, Japan.

[8] Department of Neurosurgery, Kanazawa University, Kanazawa, Japan.

[9] KAGAMI INC., 7-7-15, Saito-Asagi, Ibarak~~g~~i, Osaka, Japan

***Corresponding author**
Yasunori Iwata
13-1 Takara-machi, Kanazawa, Ishikawa, Japan 920-8641
Tel: +81-76-265-2499
Email: iwatay@staff.kanazawa-u.ac.jp

Word count
Word count abstract: 199 words (limit 200 words)
Word count manuscript: 2531 words (limit 6000 words)
Table and Figure: 3 Figures and 3 Tables
Supplemental Material: 2 Figures and 2 Table

Abbreviations
CKD: chronic kidney disease
eGFR: estimated glomerular filtration rate
HOMA-IR: homeostasis model assessment of insulin resistance
UACR: urine albumin to creatinine ratio
rRNA: ribosomal ribonucleic acid

Abstract

D-serine administration prevents kidney damage in murine models of acute kidney injury, and risperidone inhibits the activity of D-amino acid oxidase, which regulate plasma D-amino acid levels. This pilot randomized controlled trial investigated the effects of risperidone on glucose, amino acid metabolism, and kidney function in healthy adults. Healthy adults with a homeostasis model assessment of insulin resistance (HOMA-IR) of ≥ 1.6 and estimated glomerular filtration rate (eGFR) of ≥ 60 mL/min/1.73m^2 were randomly assigned to the risperidone and control groups. The risperidone group received 0.5 mg/day risperidone for 4 days. The primary outcome was mean change in HOMA-IR on day 5, and the secondary outcomes were changes in D-amino acid levels, eGFR, and urinary albumin. Seven participants were randomized to the risperidone and control groups. The changes in HOMA-IR, eGFR, and urinary albumin on day 5 were not significantly different between the two groups (all $p>0.05$). Mean changes in plasma D-serine level and urinary D-serine/creatinine ratio were significantly higher in the risperidone group than in the control group (0.2 vs. −0.3 nmol/mL, $p=0.03$ and 38.2 vs. −25.8 nmol/mL, $p=0.01$, respectively). Short-term risperidone affects D-serine metabolism without instigating acute adverse effects on kidney or glucose homeostasis in healthy individuals.

Clinical Trial Registry number: This study was registered with the Japan Registry for Clinical Trials (jRCTs041210165).

Introduction

Chronic kidney disease (CKD), which impacts more than 10% of the global population, is associated with severe outcomes, including end-stage kidney disease and cardiovascular complications.[1] Despite the demonstrated efficacy of several therapeutic agents, such as renin-angiotensin system inhibitors and sodium-glucose cotransporter-2 inhibitors, in slowing CKD progression, the residual risk for disease progression remains significant.[2, 3] Thus, the development of novel therapeutic approaches are urgently needed to mitigate the risk and progression of CKD in the general population.

The renoprotective roles of D-amino acids, such as D-serine and D-alanine, have been demonstrated in several recent in vitro and in vivo studies.[4-6] For example, the oral administration of D-serine and D-alanine alleviated kidney damage in a murine model of acute kidney injury induced by ischemia/reperfusion,[4, 5] whereas elevated D-serine levels were associated with tissue remodeling in a nephrectomy model.[6] Clinical studies have also found that the plasma D-serine and D-alanine concentrations are higher in patients with CKD than in healthy individuals,[7-9] suggesting their potential as biomarkers and therapeutic targets in kidney diseases.

Risperidone, an atypical antipsychotic agent widely used for the treatment of schizophrenia, exerts its primary effects through the antagonism of D2 dopamine and 5-HT2A serotonin receptors.[10] Additionally, risperidone also inhibits D-amino acid oxidase, which degrades D-serine and D-alanine.[11-13] In a previous observational study of patients in routine clinical care, we reported that risperidone use was associated with a reduced risk of decline in kidney function in patients with schizophrenia,[14] raising the possibility that the effects of risperidone on D-amino acid metabolism might be a contributor to its protective effect against kidney injury.

Several studies have investigated the effects of antipsychotic medications on kidney function with mixed results. While some retrospective studies suggest that atypical antipsychotics, including risperidone, may not increase the risk of acute kidney injury compared to typical antipsychotics,[15, 16] other population-based studies have reported increased risks in elderly patients.[17] The mechanisms underlying these

Formatted: Font: Not Italic

Formatted: Font: Not Italic

Formatted: Font: Not Italic

effects remain unclear, but may involve direct nephrotoxic effects or indirect effects through metabolic changes.

Despite the evidence supporting the renoprotective effect of risperidone, its impact on the amino acid profile of humans remains unclear. Additionally, the interplay of risperidone with kidney function and glucose metabolism has not been well-studied in individuals without psychiatric conditions. Therefore, we conducted a pilot randomized controlled trial to investigate the impact of short-term risperidone administration on the amino acid profile, kidney function, and glucose metabolism in healthy adults. Microbiome analysis was included because gut microbiome can metabolize D-amino acids, and we aimed to control for potential confounding effects of microbial D-amino acid metabolizm on our study endpoints. Our findings offer insights into the pharmacological effects of risperidone on D-serine and D-alanine levels, laying the foundation for novel therapeutic strategies targeting D-amino acid in patients with CKD.

Formatted: Afar (Ethiopia)

Formatted: Font: Not Bold

Formatted: Font: Not Bold

Formatted: Font: Not Bold

Formatted: Font: Not Bold

Formatted: Font: Not Bold

Formatted: Font: Not Bold

Materials and Methods

Study design and participants

This pilot, open-label, randomized controlled trial evaluated the efficacy of administration of risperidone for 4 days in healthy volunteers. The study protocol (S1 Protocol and S1 CONSORT Checklist) was approved by the Medical Ethics Committee of Kanazawa University (approval no: 2021-004) and was registered at jRCT (jRCTs041210165). The study was conducted in accordance with the the Declaration of Helsinki. The participants were recruited between March 22, 2022 and July 20, 2023. Written informed consent was obtained from all participants prior to enrollment.

The study enrolled healthy adults aged 20–65 years who met the following criteria: 1) no history of psychiatric or neurologic disorders, 2) fasting blood glucose levels < 140 mg/dL, 3) homeostasis model assessment of insulin resistance (HOMA-IR) ≥ 1.6, 4) estimated glomerular filtration rate (eGFR) ≥ 60 mL/min/1.73 m², 5) both aspartate and alanine transaminase levels < 30 IU/L, 6) no history of arrhythmias or congenital long QT syndrome, 7) no history of cancer within the past five years, and 8) presence of bowel movements at least once every four days, regardless of laxative use.

The glucose cutoff was chosen to exclude participants with overt diabetes while including those with potential insulin resistance, as measured by HOMA-IR.

Formatted: Font: Not Italic

Participants fulfilling the following criteria were excluded from the study: 1) any medically diagnosed condition currently under treatment, 2) history of hypersensitivity to risperidone, paliperidone, or their components, 3) pregnancy or breastfeeding, 4) participation in another clinical study within the last 3 months prior to the initiation of risperidone administration, 5) deemed unsuitable for study participation for any reason by the principal or sub-investigator, and 6) currently holding a faculty or staff position of the department in which the principal investigator is affiliated.

Eligible participants were randomized at a 1:1 ratio to the risperidone and control groups within 30 days of the study enrollment (S1 Fig). Randomization was centrally performed in the registration center using minimization, with age and sex as allocation adjustment factors. A sample size estimation was not performed due to the absence of prior data on effect size. This pilot study was designed to generate preliminary data for future power calculations.

Formatted: Font: Not Italic

Interventions

Participants in the risperidone group were administered 0.5 mg risperidone (Risperdal® OD, 0.5 mg) once daily at bedtime for 4 days. The lowest available dosage of 0.5 mg/day was chosen to prioritize participant safety. The 4-day treatment duration was chosen based on pharmacokinetic studies showing that steady-state levels are achieved within 3-4 days, allowing us to observe steady-state effects while minimizing exposure in healthy volunteers. To ensure compliance, participants in the risperidone group were instructed to return any remaining tablets at the end of the study for collection.

The study was conducted in an outpatient setting, and no participants was hospitalized during the intervention. Regardless of the group assignment, all participants visited the study site on days 1, 5, and 9 after study initiation. To minimize interference with the gut microbiota, participants were asked to restrict the consumption of foods containing probiotics, such as yogurt, fermented beverages, natto, miso, pickles, and kimchi, starting one week before the study initiation and continuing throughout the follow-up period. Participants were also restricted from using

antimicrobials and *Lactobacillus* preparations. Furthermore, participants were advised to avoid activities requiring alertness, such as driving or operating hazardous machinery, and to abstain from alcohol to reduce the risk of adverse events during the risperidone administration period.

Laboratory measurements

Participants underwent blood tests after fasting or at least 6 hours after meals and early morning urine tests at baseline and on days 5 and 9 after study initiation. Blood parameters that were assessed included leukocytes, erythrocytes, and platelet counts; hemoglobin; hematocrit; total protein; serum albumin; aspartate and alanine aminotransferases; blood urea nitrogen; creatinine; uric acid; sodium; potassium; calcium; inorganic phosphorus; fasting blood glucose; hemoglobin A1c; insulin; C-peptide; total cholesterol; high-density lipoprotein cholesterol; and triglycerides.

The following equation proposed by the Japanese Society of Nephrology was used to calculate eGFR: eGFR (mL/min/1.73 m^2) = 194 × serum creatinine (mg/dL) $^{-1.094}$ × age (years)$^{-0.287}$ × 0.739 (if female).[18] The urine albumin to creatinine ratio (UACR) was also assessed. All blood and urinary parameters were measured by an independent laboratory at BML Inc. (Tokyo, Japan) using automated clinical testing technology. The results of all urinary parameters were normalized to urine creatinine to correct for differences in concentrations uniquely related to the hydration status or the urine volume of the participant.[19]

Plasma and urinary levels of DL-serine and alanine at baseline and on day 5 were determined using a two-dimensional high-performance liquid chromatography system (DASH 27B3X00322000001) by KAGAMI INC. (Osaka, Japan), according to a previously described protocol with modifications.[4, 5, 8]

Intestinal bacterial flora analysis was conducted based on 16S ribosomal ribonucleic acid (rRNA) sequencing of fecal samples using QIIME2 (version 2021.2) by Takara Bio's Biomedical Center (Shiga, Japan). Fecal samples were collected by participants at home using a collection kit (FS-0017, TechnoSuruga Lab. Co., Ltd., Shizuoka, Japan) at baseline and on day 5. Microbiome analysis included alpha diversity, such as number of operational taxonomic units, Chao-1 index, and Shannon

Formatted: Font: Not Italic
Formatted: Font: Not Bold
Formatted: Font: Not Bold
Formatted: Font: Not Bold
Formatted: Font: Not Italic

index, and beta diversity, such as weighted Unifrac and Bray-Curtis distances, and relative abundance, with a sampling depth of 10,000 sequences.

Formatted: Afar (Ethiopia)

Study endpoints

The primary study endpoint was the mean change in HOMA-IR from baseline to 5 days between groups. HOMA-IR was selected as the primary endpoint because risperidone has the potential to increase glucose levels and insulin resistance,[20, 21] and we first aimed to assess the metabolic safety profile of risperidone in healthy individuals. Intention-to-treat analyses were performed according to the randomly assigned groups. The secondrary endpoints included the mean changes in the following parameters from baseline to 5 days between groups: plasma and urine DL-serine and DL-alanine levels, eGFR, blood pressure, body mass index, blood glucose, hemoglobin A1c, insulin, C-peptide, and UACR. Safety was monitored by assessing adverse events, medical interviews, and the review of laboratory values during the study period.

Statistical analysis

For baseline participant characteristics, continuous variables were presented as means with standard deviations and categorical variables were presented as numbers with percentages. For the primary and secondary endpoints, Student's *t*-test was used to compare mean changes in clinical and laboratory parameters from baseline to days 5 and 9 between the risperidone and control groups. Given the small sample size, Mann-Whitney U test was also performed as a non-parametric analysis to complement the parametric tests. All analyses were performed using Stata version 18 (Stata Corp, College Station, TX). A two-sided p value of <0.05 was considered statistically significant.

Formatted: Font: Not Italic, No underline

Formatted: Font: Not Italic, No underline

Formatted: Font: Not Italic, No underline

Formatted: Font: Not Italic, No underline

Results

Baseline characteristics of the participants

Between March and November 2022, a total of 8 participants were randomized to the risperidone and control groups (n = 4 participants per group), 1 participant declined to complete the study, and 7 completed the protocol treatment (Fig 1). In the risperidone and control groups, the mean participant age was 35 and 29 years, 1 (33%) and 2 (50%) of the participants were male, the mean HOMA-IR was 2.0 and 1.6, and the

mean eGFR was 90 and 92 mL/min/1.73 m^2, respectively (Table 1). The baseline participant characteristics were comparable between the two groups (all $p > 0.10$), except for body mass index, which was likely to be higher in the risperidone group than in the control group ($p = 0.03$).

Fig 1. Flowchart of the study

Table 1. Baseline participant characteristics in the risperidone and control groups

	**Risperidone (n = 3)**	**Control (n = 4)**	**p**
Age (years)	35 (12)	29 (8)	0.49
Male	1 (33)	2 (50)	0.66
Current smoking	0 (0)	0 (0)	–
Drinking habit	1 (33)	1 (25)	0.81
Body weight (kg)	66 (9)	61 (7)	0.41
Body mass index (kg/m^2)	23.8 (1.4)	21.2 (0.8)	0.03
Systolic blood pressure (mmHg)	105 (6)	113 (16)	0.45
Diastolic blood pressure (mmHg)	71 (9)	74 (5)	0.69
Aspartate aminotransferase (U/L)	16 (4)	19 (6)	0.62
Alanine aminotransferase (U/L)	12 (6)	16 (7)	0.50
eGFR (mL/min/1.73 m^2)	90 (20)	92 (13)	0.89
Uric acid (mg/dL)	4.6 (1.0)	6.3 (2.0)	0.25
Fasting blood glucose (mg/dL)	92 (9)	90 (4)	0.67
Hemoglobin A1c (%)	4.9 (0.1)	5.0 (0.2)	0.61
Insulin (μU/mL)	8.9 (2.0)	7.3 (2.9)	0.44
C peptide (ng/mL)	1.9 (0.4)	1.6 (0.5)	0.33
HOMA-IR	2.0 (0.4)	1.6 (0.6)	0.36
HOMA-β (%)	119 (57)	100 (43)	0.64
Total cholesterol (mg/dL)	227 (35)	183 (23)	0.10
HDL-cholesterol (mg/dL)	66 (21)	67 (16)	0.97
Triglycerides (mg/dL)	82 (19)	73 (23)	0.60
Urine albumin/Cr (mg/g Cr)	2.9 (1.1)	4.7 (2.9)	0.38
**Plasma amino acid**			
D-serine (nmol/mL)	1.8 (0.3)	1.9 (0.1)	0.52
L-serine (nmol/mL)	120.4 (18.4)	133.3 (17.8)	0.39
D/L-serine (%)	1.5 (0.5)	1.4 (0.2)	0.77
D-alanine (nmol/mL)	1.0 (0.2)	0.8 (0.7)	0.75
L-alanine (nmol/mL)	354.3 (50.4)	288.5 (59.8)	0.19
D/L-alanine (%)	0.3 (0.1)	0.3 (0.2)	0.96
**Urine amino acid**			
D-serine/Cr (mmol/g Cr)	123.4 (10.0)	136.6 (18.3)	0.31
L-serine/Cr (mmol/g Cr)	118.9 (53.3)	256.6 (252.1)	0.40

D-alanine/Cr (mmol/g Cr)	27.1 (9.5)	23.7 (19.6)	0.80
L-alanine/Cr (mmol/g Cr)	142.3 (75.5)	113.7 (46.3)	0.56

Abbreviations; eGFR, estimated glomerular filtration rate; HOMA-IR, Homeostatic model assessment of insulin resistance; HOMA-β, Homeostatic model assessment of beta cell function; HDL, high density lipoprotein; Cr, creatinine.

Data are described as mean (standard deviation) for continuous variables or n (%) for categorical variables. Statistical comparisons were performed using Student's *t*-test for continuous variables and Fisher's exact test for categorical variables.

Formatted Table

Formatted: Font: Italic

Comparison of changes in DL-amino acid levels

At baseline, the mean (± SD) plasma D-serine levels were 1.8 ± 0.3 nmol/mL and 1.9 ± 0.1 nmol/mL in the risperidone and control groups, respectively. The mean plasma D-serine level increased by 0.24 ± 0.36 nmol/mL on day 5 compared to the baseline in the risperidone group and decreased by −0.32 ± 0.13 nmol/mL during the same time period in the control group (Fig 2A and S1 Table). The mean plasma D-serine level was 0.56 (95% CI 0.07–1.05) nmol/mL higher in the risperidone group than in the control group on day 5 after the study initiation (p = 0.03). Similarly, the mean change in urinary D-serine-to-creatinine ratio was significantly higher in the risperidone group than in the control group (38.2 ± 19.8 and −25.8 ± 23.4 mmol/g Cr; respectively; between-group difference: 64.0 mmol/g Cr, 95% CI 20.8–107.2; p = 0.01) compared to the control group. The significant differences in plasma D-serine levels and urinary D-serine-to-creatinine ratio between groups remained consistent when analyzed using Mann-Whitney U tests (S1 Table).

Formatted: English (United States)

Fig 2. Mean plasma and urine DL-serine (A) and DL-alanine (B) levels during follow-up in the risperidone and control groups

The mean changes in plasma D-alanine level and urinary D-alanine-to-creatinine ratio were numerically higher in the risperidone group than in the control group, with no statistical difference (0.54 vs. −0.31 nmol/mL, p = 0.25 and 24.98 vs. −9.42 nmol/mL, p = 0.20, respectively) (Fig 2B and S1 Table). Conversely, no significant changes were observed in plasma and urinary L-serine and L-alanine levels during the follow-up period and these parameters did not significantly differ between the two groups (Fig 2 and S1 Table).

Effects of risperidone on clinical and laboratory parameters

The mean change in HOMA-IR was not statistically significantly different between the risperidone and control groups during the treatment period (0.88 ± 0.58 and 0.42 ± 1.00, p = 0.51) (Fig 3 and Table 2). The change in mean blood glucose from the baseline to day 5 after study initiation was marginally higher in the risperidone group than in the control group, by Student's t-test (4.0 ± 2.0 vs. −1.3 ± 3.0 mg/dL, respectively; p = 0.048), however this difference was not significant when analyzed using the Mann-Whitney U test (p = 0.07). The mean change in eGFR did not differ between the risperidone and control groups (1.0 ± 8.7 and −0.4 ± 15.6 mL/min/1.73 m^2, respectively; p = 0.90). The changes in mean blood pressure, body mass index, hemoglobin A1c, insulin, C-peptide, and UACR were not statistically significantly different between the two groups (Table 2). All other parameters except fasting blood glucose showed consistent results between parametric and non-parametric analyses.

Formatted: Underline

Formatted: Font: Not Italic, Underline

Formatted: Underline

Formatted: Font: Not Italic

Formatted: Font: Not Italic

Fig 3. Mean homeostatic model assessment of insulin resistance (HOMA-IR) (A), fasting blood glucose (B), and estimated glomerular filtration rate (eGFR) (C) levels during follow-up in the risperidone and control groups

Table 2. Mean changes in clinical and laboratory parameters in the risperidone and control groups

		**Mean (SD) change from baseline**			
	**Day**	**Risperidone (n = 3)**	**Control (n = 4)**	**p***	**p****
HOMA-IR	5	0.88 (0.58)	0.42 (1.0)	0.51	0.29
	9	0.03 (1.25)	0.15 (0.88)	0.88	1.00
Systolic blood pressure (mmHg)	5	7.7 (18.0)	–3.3 (7.7)	0.32	0.29
	9	8.0 (19.5)	1.8 (15.3)	0.65	0.48
Diastolic blood pressure (mmHg)	5	–2.3 (13.7)	–3.5 (2.5)	0.87	0.48
	9	1.0 (8.9)	–2.3 (8.7)	0.65	0.48
Body mass index (kg/m^2)	5	0.1 (0.2)	–0.2 (0.2)	0.10	0.08
	9	–0.03 (0.2)	–0.06 (0.2)	0.87	1.00
eGFR (mL/min/1.73 m^2)	5	1.0 (8.7)	–0.4 (15.6)	0.90	0.72
	9	1.8 (10.7)	0.02 (2.8)	0.76	0.72
Fasting blood glucose (mg/dL)	5	4.0 (2.0)	–1.3 (3.0)	0.048	0.07
	9	–7.7 (4.7)	–3.0 (3.8)	0.21	0.21
Hemoglobin A1c (%)	5	0.00 (0.10)	0.08 (0.05)	0.24	0.29
	9	0.07 (0.06)	0.05 (0.06)	0.72	1.00

Insulin (μU/mL)	5	3.2 (2.1)	1.9 (4.0)	0.62	0.48
	9	1.2 (5.8)	0.9 (3.8)	0.95	1.00
C peptide (ng/mL)	5	0.90 (0.95)	0.20 (0.59)	0.28	0.16
	9	0.03 (0.67)	0.03 (0.59)	0.99	0.86
Urine albumin/Cr (mg/g Cr)	5	–0.1 (1.0)	–0.3 (2.4)	0.92	0.72
	9	–0.4 (1.1)	–1.4 (2.2)	0.50	0.48

Abbreviations; HOMA-IR, Homeostatic model assessment of insulin resistance; eGFR, estimated glomerular filtration rate; Cr, creatinine. P* values were calculated using Student's t-test and p** values were calculated using Mann-Whitney U test as non-parametric analysis.

Formatted: Font: 11 pt

Exploratory analysis to assess the effects of risperidone on additional clinical and laboratory parameters (S2 Table) revealed no significant changes in body weight, HOMA for β-cell function, aspartate and alanine aminotransferase, uric acid, total and high-density lipoprotein cholesterol, and triglycerides after 4 days of risperidone administration.

Effects of risperidone on the fecal microbiome

In the 16S rRNA analysis, no significant differences were observed in alpha diversity (species richness) metrics of the fecal microbiome on day 5 compared to the baseline in both risperidone and control group (paired *t*-test, $p > 0.10$) (S2 Fig). For beta diversity (overall structural similarity and variation), the weighted Unifrac distances were visually unchanged, and the Gray-Curtis distances were similar ($p = 0.81$) between the risperidone and control groups during the treatment period (S2 Fig). Phylum-level analysis revealed that both groups were dominated by Firmicutes and Bacteroidetes, accounting for over 80% of the microbiome composition at baseline and no significant changes were observed in the composition at day 5 in either group (S2 Fig and S3 Table).

Safety

All adverse events observed during the study are presented in Table 3. All participants reported a total of eight non-serious adverse events. In the risperidone group, two participants experienced somnolence, and one participant experienced abdominal distension. One participant in each group reported blood pressure of > 140/90 mmHg. No participant in the risperidone group reported hypotension or dizziness, and no serious adverse events were recorded.

Table 3. Safety in the risperidone and control groups

	N (%) Risperidone (n = 3)	Control (n = 4)
Somnolence	2 (67)	0 (0)
Hypertension	1 (33)	1 (25)
Abdominal distension	1 (33)	0 (0)
Hypertriglyceridemia	0 (0)	1 (25)
Hypercholesterolemia	0 (0)	1 (25)
Liver injury	0 (0)	1 (25)
Hypotension	0 (0)	0 (0)
Dizziness	0 (0)	0 (0)

Discussion

In this open-label, randomized pilot clinical trial, we found that the short-term administration of risperidone led to an increase in plasma and urinary D-serine levels in healthy adults with normal kidney function. Additionally, risperidone did not adversely eGFR levels or insulin resistance. Overall, these results suggest that risperidone impacts D-serine metabolism without an acute adverse impact on kidney function or glucose homeostasis in healthy individuals.

The significant elevation in D-serine levels observed immediately after risperidone administration is a notable finding, which is likely attributable to risperidone-mediated inhibition of D-amino acid oxidase, preventing D-serine degradation.[11, 22] The contribution of dietary and microbial factors to D-serine levels should also be considered. D-serine is present in various food sources, including fermented products rich in D-amino acids due to microbial fermentation by serine racemase.[23-26] Although fecal D-serine levels were not measured in the present study, we aimed to minimize confounding mediated dietary factors through the restriction of the intake of fermented foods, antibiotics, and lactic acid supplements during the study period. In addition, the lack of significant changes in gut microbiome diversity during the short treatment period suggests that the increases in D-serine levels were primarily due to direct enzyme inhibition rather than microbial effects.~~,~~ These findings ensured~~ing~~ that the observed changes in D-serine were attributable to risperidone administration rather than the impact of dietary or gut microbiota-related factors.

Formatted: English (United States)

Given the small sample size of this pilot study, the magnitude of observed effects may be more informative than statistical significance. The substantial increase in plasma D-serine levels (0.56 nmol/mL difference, 95% CI 0.07–1.05) and urinary D-serine-to-creatinine ratio (64.0 mmol/g Cr difference, 95% CI 20.8–107.2) represent clinically meaningful changes that warrant further investigation in larger studies. Similarly, the numerical increase observed in plasma D-alanine levels and urinary D-alanine-to-creatinine ratio, while not reaching statistical significance, provide valuable preliminary data for effect size calculations in future trial designs.

Short-term risperidone administration did not affect kidney function in healthy individuals enrolled in our study. Few studies have investigated the efficacy and safety of risperidone on kidney function, with contradictory findings. Results from several retrospective cohort studies suggest that risperidone does not increase the risk of acute kidney injury compared to typical antipsychotics.[15, 16] In a previous retrospective observational study, we also found that risperidone use was associated with a lower risk of decline in kidney function in patients with schizophrenia.[14] However, a population-based cohort study reported an increased risk of acute kidney injury-related hospitalizations in elderly patients treated with atypical antipsychotics, including risperidone.[17] The absence of acute adverse effects on kidney function in healthy participants in our study contrasts with some previous reports of antipsychotic-associated nephrotoxicity, which may be related to our study population (healthy individuals vs. patients with comorbidities) or the short duration of exposure. These discrepancies underscore the need for further studies to elucidate the kidney safety profile of risperidone particularly in vulnerable populations such as elderly individuals and patients with CKD.

We also found a slight increase in blood glucose levels during risperidone administration. Given the high variability of glucose levels influenced by diet and lifestyle, this change might not be directly related to risperidone administration. Additionally, no significant changes were observed in other glucose metabolism markers, including HOMA-IR and insulin, indicating that risperidone might have a limited impact on glycemic control during short-term use. These results align with previous studies reporting the relatively modest effect of risperidone on glucose metabolism

compared to other atypical antipsychotics, which are known for their association with metabolic side effects.[27, 28]

This study has several limitations. First, the open-label design might have introduced performance bias due to the lack of blinding. Second, we focused exclusively on a healthy population in a pilot study with a small samle size and short follow-up period, which limited the robustness of the safety data. Any inferences should be interpreted with caution. The small sample size limits our findings and any inferences should be interpreted with caution. Third, the assumption of normality for Student's *t*-test may not be reliably verified with small sample sizes, which represents a limitation of our statistical approach. Longer-term clinical trials with larger sample sizes in patients with CKD are warranted to assess the sustained effects and safety of risperidone in this patient population. Fourth, we did not specifically control for protein intake during the study period, which may have influenced kidney parameters. Finally, the study was conducted in a Japanese population and our findings might not be generalizable to other ethnic groups with different genetic or dietary backgrounds.

Formatted: No underline

Formatted: Font: Not Italic, No underline

Formatted: Font: Italic

Formatted: Font: Not Italic

Formatted: Font: Not Italic

Conclusion

In conclusion, short-term administration of risperidone at 0.5 mg over 4 days effectively increased plasma D-serine concentrations in healthy participants. Although a slight increase in blood glucose levels was observed, other markers of glucose metabolism and kidney function remained stable during the study period. These findings suggest that risperidone modulates D-serine metabolism without acute adverse effects on kidney function or glucose homeostasis in healthy individuals. These findings provide a foundation for future studies exploring the therapeutic applications of risperidone in kidney disease, and the potential safety and efficacy of risperidone in modulating D-serine metabolism warrant further investigation in clinical trials including patients with CKD.

Acknowledgments

The authors thank the participants who took part in the study. We would like to acknowledge Yuko Oyama, Mari Shimizu, and Eri Umeda at Kanazawa University, who

have assisted in sample collection and processing. We also thank Hiroshi Imoto, Eiichi Negishi, Maiko Nakane, and Shoto Ishigo in KAGAMI INC. who have contributed to the development of alanine powder formulation and chiral amino acid analysis.

Financial Disclosure Statement

This study was supported by KAGAMI INC.. MM is the founder and CEO of KAGAMI INC., a startup company working on chiral amino acid analysis and research for medical applications. The funder provided blinded amino acid level measurements, but had no role in study design, data collection and analysis. MO is supported by a grant from the 2021 public offering research on clinical research by Kanazawa University Hospital and Initiative for Realizing Diversity in the Research Environment, MEXT. Other authors did not receive any specific grant from funding agencies in the public, commercial, or not-for-profit sectors.

References

1. United States Renal Data System. 2023 USRDS Annual Data Report: Epidemiology of kidney disease in the United States. National Institutes of Health, National Institute of Diabetes and Digestive and Kidney Diseases, Bethesda, MD: , 2023. Report No.

2. Xie X, Liu Y, Perkovic V, Li X, Ninomiya T, Hou W, et al. Renin-Angiotensin System Inhibitors and Kidney and Cardiovascular Outcomes in Patients With CKD: A Bayesian Network Meta-analysis of Randomized Clinical Trials. Am J Kidney Dis. 2016;67(5):728-41. Epub 2015/11/26. doi: 10.1053/j.ajkd.2015.10.011. PubMed PMID: 26597926.

3. Nuffield Department of Population Health Renal Studies G, Consortium SiM-AC-RT. Impact of diabetes on the effects of sodium glucose co-transporter-2 inhibitors on kidney outcomes: collaborative meta-analysis of large placebo-controlled trials. Lancet. 2022;400(10365):1788-801. Epub 2022/11/10. doi: 10.1016/S0140-6736(22)02074-8. PubMed PMID: 36351458; PubMed Central PMCID: PMCPMC7613836.

4. Nakade Y, Iwata Y, Furuichi K, Mita M, Hamase K, Konno R, et al. Gut microbiota-derived D-serine protects against acute kidney injury. JCI Insight. 2018;3(20). Epub 2018/10/20. doi: 10.1172/jci.insight.97957. PubMed PMID: 30333299; PubMed Central PMCID: PMCPMC6237464.

5. Iwata Y, Nakade Y, Kitajima S, Yoneda-Nakagawa S, Oshima M, Sakai N, et al. Protective effect of d-alanine against acute kidney injury. Am J Physiol Renal Physiol. 2022;322(6):F667-F79. Epub 2022/04/19. doi: 10.1152/ajprenal.00198.2021. PubMed PMID: 35435002.

6. Hesaka A, Tsukamoto Y, Nada S, Kawamura M, Ichimaru N, Sakai S, et al. d-Serine Mediates Cellular Proliferation for Kidney Remodeling. Kidney360. 2021;2(10):1611-24. Epub 2022/04/05. doi: 10.34067/KID.0000832021. PubMed PMID: 35372967; PubMed Central PMCID: PMCPMC8785787.

7. Nakade Y, Iwata Y, Sakai N, Mita M, Nakane M, Hamase K, et al. Increased levels of oral Streptococcus-derived D-alanine in patients with chronic kidney disease and diabetes mellitus. Sci Rep. 2022;12(1):21773. Epub 2022/12/17. doi: 10.1038/s41598-022-26175-1. PubMed PMID: 36526888; PubMed Central PMCID: PMCPMC9758232.

8. Ishii C, Akita T, Mita M, Ide T, Hamase K. Development of an online two-dimensional high-performance liquid chromatographic system in combination with tandem mass spectrometric detection for enantiomeric analysis of free amino acids in human physiological fluid. J Chromatogr A. 2018;1570:91-8. Epub 2018/08/15. doi: 10.1016/j.chroma.2018.07.076. PubMed PMID: 30104059.

9. Iwata Y, Okushima H, Hesaka A, Kawamura M, Imamura R, Takahara S, et al. Identification of Diabetic Nephropathy in Patients Undergoing Kidney Biopsy through Blood and Urinary Profiles of d-Serine. Kidney360. 2021;2(11):1734-42. Epub 2022/04/05. doi: 10.34067/KID.0004282021. PubMed PMID: 35372995; PubMed Central PMCID: PMCPMC8785851.

10. Tandon R. Antipsychotics in the treatment of schizophrenia: an overview. J Clin Psychiatry. 2011;72 Suppl 1:4-8. Epub 2012/01/11. doi: 10.4088/JCP.10075su1.01. PubMed PMID: 22217436.

11. Shishikura M, Hakariya H, Iwasa S, Yoshio T, Ichiba H, Yorita K, et al. Evaluation of human D-amino acid oxidase inhibition by anti-psychotic drugs in vitro. Biosci Trends. 2014;8(3):149-54. Epub 2014/07/18. doi: 10.5582/bst.2014.01034. PubMed PMID: 25030849.
12. Onozato M, Nakazawa H, Hakariya H, Shishikura M, Nagashima C, Ishimaru K, et al. Effect of risperidone on plasma d-serine concentration in rats post-administered with d-serine. Life Sci. 2016;158:98-103. Epub 2016/06/30. doi: 10.1016/j.lfs.2016.06.021. PubMed PMID: 27352936.
13. Abou El-Magd RM, Park HK, Kawazoe T, Iwana S, Ono K, Chung SP, et al. The effect of risperidone on D-amino acid oxidase activity as a hypothesis for a novel mechanism of action in the treatment of schizophrenia. J Psychopharmacol. 2010;24(7):1055-67. Epub 2009/03/31. doi: 10.1177/0269881109102644. PubMed PMID: 19329549.
14. Oshima M, Toyama T, Nakade Y, Yomogida D, Yuasa T, Horikoshi K, et al. Association Between Risperidone Use and Kidney Function Decline in Patients with Schizophrenia: A Retrospective Cohort Study. Clin Ther. 2023;45(9):889-93. Epub 2023/07/25. doi: 10.1016/j.clinthera.2023.07.002. PubMed PMID: 37487866.
15. Jiang Y, McCombs JS, Park SH. A Retrospective Cohort Study of Acute Kidney Injury Risk Associated with Antipsychotics. CNS Drugs. 2017;31(4):319-26. Epub 2017/03/16. doi: 10.1007/s40263-017-0421-4. PubMed PMID: 28290080.
16. Sharon R, Lange T, Aakjaer M, Brogger Kristiansen S, Baltzer Houlind M, Andersen M. Incidence of hospital contacts with acute kidney injury after initiation of second-generation antipsychotics in older adults: a Danish population-based cohort study. Eur J Clin Pharmacol. 2022;78(8):1341-9. Epub 2022/06/01. doi: 10.1007/s00228-022-03339-6. PubMed PMID: 35639132; PubMed Central PMCID: PMCPMC9283184.
17. Hwang YJ, Dixon SN, Reiss JP, Wald R, Parikh CR, Gandhi S, et al. Atypical antipsychotic drugs and the risk for acute kidney injury and other adverse outcomes in older adults: a population-based cohort study. Ann Intern Med. 2014;161(4):242-8. Epub 2014/08/19. doi: 10.7326/M13-2796. PubMed PMID: 25133360.
18. Matsuo S, Imai E, Horio M, Yasuda Y, Tomita K, Nitta K, et al. Revised equations for estimated GFR from serum creatinine in Japan. Am J Kidney Dis. 2009;53(6):982-92. Epub 2009/04/03. doi: 10.1053/j.ajkd.2008.12.034. PubMed PMID: 19339088.
19. Bienaime F, Muorah M, Metzger M, Broeuilh M, Houiller P, Flamant M, et al. Combining robust urine biomarkers to assess chronic kidney disease progression. EBioMedicine. 2023;93:104635. Epub 2023/06/07. doi: 10.1016/j.ebiom.2023.104635. PubMed PMID: 37285616; PubMed Central PMCID: PMCPMC10279781.
20. Boyda HN, Procyshyn RM, Tse L, Yuen JWY, Honer WG, Barr AM. A comparison of the metabolic side-effects of the second-generation antipsychotic drugs risperidone and paliperidone in animal models. PLoS One. 2021;16(1):e0246211. Epub 2021/01/29. doi: 10.1371/journal.pone.0246211. PubMed PMID: 33508013; PubMed Central PMCID: PMCPMC7842964 following competing interests: WGH has received consulting fees or sat on paid advisory boards for Otsuka/Lundbeck, Newron, AlphaSights, Guidepoint, Translational Life Sciences and holds shares in Translational Life Sciences and Eli Lilly. He was additionally supported by the Jack Bell Chair in Schizophrenia. RMP has been a member of the following advisory boards in the past 3

years: Janssen, Lundbeck, and Otsuka; a member of the following speaker's bureaus in the past 3 years: Janssen, Lundbeck, and Otsuka; and received grants from the Canadian Institutes of Health Research. All other authors declare that no competing interests exist. This does not alter our adherence to PLOS ONE policies on sharing data and materials.

21. Koller EA, Cross JT, Doraiswamy PM, Schneider BS. Risperidone-associated diabetes mellitus: a pharmacovigilance study. Pharmacotherapy. 2003;23(6):735-44. Epub 2003/06/25. doi: 10.1592/phco.23.6.735.32178. PubMed PMID: 12820816.

22. Szilagyi B, Ferenczy GG, Keseru GM. Drug discovery strategies and the preclinical development of D-amino-acid oxidase inhibitors as antipsychotic therapies. Expert Opin Drug Discov. 2018;13(10):973-82. Epub 2018/09/18. doi: 10.1080/17460441.2018.1524459. PubMed PMID: 30220232.

23. Kato S, Ishihara T, Hemmi H, Kobayashi H, Yoshimura T. Alterations in D-amino acid concentrations and microbial community structures during the fermentation of red and white wines. J Biosci Bioeng. 2011;111(1):104-8. Epub 2010/09/28. doi: 10.1016/j.jbiosc.2010.08.019. PubMed PMID: 20869309.

24. Tian H, Zheng N, Li S, Zhang Y, Zhao S, Wen F, et al. Characterization of chiral amino acids from different milk origins using ultra-performance liquid chromatography coupled to ion-mobility mass spectrometry. Sci Rep. 2017;7:46289. Epub 2017/04/11. doi: 10.1038/srep46289. PubMed PMID: 28393862; PubMed Central PMCID: PMCPMC5385494.

25. Suzuki M, Shimizu-Hirota R, Mita M, Hamase K, Sasabe J. Chiral resolution of plasma amino acids reveals enantiomer-selective associations with organ functions. Amino Acids. 2022;54(3):421-32. Epub 2022/03/01. doi: 10.1007/s00726-022-03140-w. PubMed PMID: 35226151.

26. Sasabe J, Miyoshi Y, Rakoff-Nahoum S, Zhang T, Mita M, Davis BM, et al. Interplay between microbial d-amino acids and host d-amino acid oxidase modifies murine mucosal defence and gut microbiota. Nat Microbiol. 2016;1(10):16125. Epub 2016/09/28. doi: 10.1038/nmicrobiol.2016.125. PubMed PMID: 27670111; PubMed Central PMCID: PMCPMC5074547.

27. Yanik T, Kursungoz C, Sutcigil L, Ak M. Weight gain in risperidone therapy: investigation of peripheral hypothalamic neurohormone levels in psychotic patients. J Clin Psychopharmacol. 2013;33(5):608-13. Epub 2013/06/29. doi: 10.1097/JCP.0b013e318297980e. PubMed PMID: 23807284.

28. Stogios N, Smith E, Bowden S, Tran V, Asgariroozbehani R, McIntyre WB, et al. Metabolic adverse effects of off-label use of second-generation antipsychotics in the adult population: a systematic review and meta-analysis. Neuropsychopharmacology. 2022;47(3):664-72. Epub 2021/08/28. doi: 10.1038/s41386-021-01163-7. PubMed PMID: 34446830; PubMed Central PMCID: PMCPMC8782876.

Supporting information

S1 Protocol. Study protocol in Japanese and Japanese to English translation.

S1 CONSORT Checklist.

S1 Fig. Study design

S2 Fig. Alpha (A) and beta (B) diversities of the fecal microbiome in the risperidone and control groups

S1 Table. Mean changes in plasma and urine dl-serine and alanine levels in the risperidone and control groups. Abbreviations; Cr, creatinine.

S2 Table. Mean changes in clinical and laboratory parameters in the risperidone and control groups. Abbreviations; HOMA-β, Homeostatic model assessment of beta cell function; HDL, high density lipoprotein.

S3 Table. Relative abundance of the fecal microbiome during follow-up in the risperidone and control groups.

Formatted: Font: Bold

Megumi Oshima and Yasunori Iwata
Department of Nephrology and Rheumatology
Kanazawa University
Kanazawa, Japan
13-1 Takara-machi, Kanazawa, Ishikawa
Japan 920-8641

August 11, 2025

Prof. Kyle J Burghardt
Academic editor
PLOS One

Dear Prof. Kyle J Burghardt

We thank the editors and reviewers for considering our manuscript and providing us the opportunity to revise our manuscript, entitled "**Effects of risperidone on amino acid metabolism, glucose, and kidney function in healthy adults: A pilot randomized controlled trial**" (manuscript ID PONE-D-25-19801).

We have revised the manuscript based on the comments and suggestions provided by the editors and reviewers. Please find below our point-by-point responses to all comments of the editors and reviewers.

This study was supported by KAGAMI INC.. MM is the founder and CEO of KAGAMI INC., a startup company working on chiral amino acid analysis and research for medical applications. The funder provided blinded amino acid level measurements, but had no role in study design, data collection and analysis. MO is supported by a grant from the 2021 public offering research on clinical research by Kanazawa University Hospital and Initiative for Realizing Diversity in the Research Environment, MEXT. Other authors did not receive any specific grant from funding agencies in the public, commercial, or not-for-profit sectors.
Data cannot be made publicly available due to the small sample size and potential participant identification, but de-identified data will be available upon reasonable request to the corresponding author. This does not alter our adherence to PLOS ONE policies on sharing data and materials.

We hope these responses and revisions meet your expectations and that the manuscript is now acceptable for publication in *PLOS One*.

Sincerely,
Megumi Oshima and Yasunori Iwata

Telephone number: +81 76 265 2499
Fax: +81 76 234 4273
Email: moshima@staff.kanazawa-u.ac.jp

COMMENTS TO THE AUTHOR:

Reviewer 1:

1. It would be informative to know whether there is any difference in the baseline characters between the two groups.

Response: Thank you for this important comment. We have added statistical comparisons of baseline characteristics between the two groups to the results section and Table 1 in the revised manuscript.

Results (page 10):

The baseline participant characteristics were comparable between the two groups (all $p > 0.10$), except for body mass index, which was likely to be higher in the risperidone group than in the control group ($p = 0.03$).

Table 1 (page 9-10):

Table 1. Baseline participant characteristics in the risperidone and control groups

	Risperidone (n = 3)	Control (n = 4)	p
Age (years)	35 (12)	29 (8)	0.49
Male	1 (33)	2 (50)	0.66
Current smoking	0 (0)	0 (0)	–
Drinking habit	1 (33)	1 (25)	0.81
Body weight (kg)	66 (9)	61 (7)	0.41
Body mass index (kg/m^2)	23.8 (1.4)	21.2 (0.8)	0.03
Systolic blood pressure (mmHg)	105 (6)	113 (16)	0.45
Diastolic blood pressure (mmHg)	71 (9)	74 (5)	0.69
Aspartate aminotransferase (U/L)	16 (4)	19 (6)	0.62
Alanine aminotransferase (U/L)	12 (6)	16 (7)	0.50
eGFR (mL/min/1.73 m^2)	90 (20)	92 (13)	0.89
Uric acid (mg/dL)	4.6 (1.0)	6.3 (2.0)	0.25
Fasting blood glucose (mg/dL)	92 (9)	90 (4)	0.67
Hemoglobin A1c (%)	4.9 (0.1)	5.0 (0.2)	0.61
Insulin (μU/mL)	8.9 (2.0)	7.3 (2.9)	0.44
C peptide (ng/mL)	1.9 (0.4)	1.6 (0.5)	0.33
HOMA-IR	2.0 (0.4)	1.6 (0.6)	0.36
HOMA-β (%)	119 (57)	100 (43)	0.64
Total cholesterol (mg/dL)	227 (35)	183 (23)	0.10
HDL-cholesterol (mg/dL)	66 (21)	67 (16)	0.97
Triglycerides (mg/dL)	82 (19)	73 (23)	0.60
Urine albumin/Cr (mg/g Cr)	2.9 (1.1)	4.7 (2.9)	0.38
Plasma amino acid			
D-serine (nmol/mL)	1.8 (0.3)	1.9 (0.1)	0.52
L-serine (nmol/mL)	120.4 (18.4)	133.3 (17.8)	0.39
D/L-serine (%)	1.5 (0.5)	1.4 (0.2)	0.77

D-alanine (nmol/mL)	1.0 (0.2)	0.8 (0.7)	0.75
L-alanine (nmol/mL)	354.3 (50.4)	288.5 (59.8)	0.19
D/L-alanine (%)	0.3 (0.1)	0.3 (0.2)	0.96
Urine amino acid			
D-serine/Cr (mmol/g Cr)	123.4 (10.0)	136.6 (18.3)	0.31
L-serine/Cr (mmol/g Cr)	118.9 (53.3)	256.6 (252.1)	0.40
D-alanine/Cr (mmol/g Cr)	27.1 (9.5)	23.7 (19.6)	0.80
L-alanine/Cr (mmol/g Cr)	142.3 (75.5)	113.7 (46.3)	0.56

Abbreviations; eGFR, estimated glomerular filtration rate; HOMA-IR, Homeostatic model assessment of insulin resistance; HOMA-β, Homeostatic model assessment of beta cell function; HDL, high density lipoprotein; Cr, creatinine.
Data are described as mean (standard deviation) for continuous variables or n (%) for categorical variables. Statistical comparisons were performed using Student's t-test for continuous variables and Fisher's exact test for categorical variables.

2. The sample size is extremely small. The inference based on this pilot study is questionable to some extent.

Response: We acknowledge that the small sample size is a significant limitation of this pilot study and have emphasized the limitation in the discussion section in the revised manuscript.

Discussion (page 15):
This study has several important limitations…. Second, we focused exclusively on a healthy population in a pilot study with a small sample size and short follow-up period, which limited the robustness of the safety data. The small sample size limits our findings and any inferences should be interpreted with caution…. Longer-term clinical trials with larger sample sizes in patients with CKD are warranted to assess the sustained effects and safety of risperidone in this patient population.

3. Student's t-test assumes normality and equal variances. How to check the normality with such a small sample?

Response: We agree with the reviewer's comment and have added Mann-Whitney U tests as non-parametric analyses to complement Student's t-tests. The results remained consistent between parametric and non-parametric approaches, except for fasting blood glucose where the Mann-Whitney U test did not show a significant difference. We have added these results and limitation to the revised manuscript.

Methods, Statistical analysis (page 8):
….For the primary and secondary endpoints, Student's *t*-test was used to compare mean changes in clinical and laboratory parameters from baseline to days 5 and 9 between the risperidone and control groups. *Given the small sample size, Mann-Whitney U test was also performed as a non-parametric analysis to complement the parametric tests.*

Results (page 10):

…. The significant differences in plasma d-serine levels and urinary D-serine-to-creatinine ratio between groups remained consistent when analyzed using Mann-Whitney U tests (S1 Table).

Results (page 11):
…. The mean change in fasting blood glucose was marginally higher in the risperidone group than in the control group by Student's t-test (4.0 ± 2.0 vs. −1.3 ± 3.0 mg/dL, p = 0.048), however this difference was not significant when analyzed using the Mann-Whitney U test (p = 0.07). …. All other parameters except fasting blood glucose showed consistent results between parametric and non-parametric analyses.

Discussion (page 15):
…. Third, the assumption of normality for Student's t-test may not be reliably verified with small sample sizes, which represents a limitation of our statistical approach. ….

S1 Table:
S1 Table. Mean changes in plasma and urine dl-serine and alanine levels in the risperidone and control groups

	Day	Mean (SD) change from baseline		p*	p**
		**Risperidone (n = 3)**	**Control (n = 4)**		
**Plasma amino acid**					
D-serine (nmol/ml)	5	0.24 (0.36)	–0.32 (0.13)	0.03	0.03
L-serine (nmol/ml)	5	2.93 (15.28)	–12.65 (29.22)	0.44	0.29
D/L-serine (%)	5	0.12 (0.05)	–0.11 (0.23)	0.16	0.03
D-alanine (nmol/ml)	5	0.54 (1.03)	–0.31 (0.70)	0.25	0.40
L-alanine (nmol/ml)	5	–24.67 (44.09)	–23.25 (69.26)	0.98	0.43
D/L-alanine (%)	5	0.17 (0.24)	–0.09 (0.28)	0.25	0.13
**Urine amino acid**					
D-serine/Cr (mmol/g Cr)	5	38.16 (19.77)	–25.84 (23.41)	0.01	0.03
L-serine/Cr (mmol/g Cr)	5	53.57 (95.66)	–70.40 (47.96)	0.07	0.08
D-alanine/Cr (mmol/g Cr)	5	24.98 (38.59)	–9.42 (23.35)	0.20	0.16
L-alanine/Cr (mmol/g Cr)	5	32.20 (52.86)	11.85 (71.62)	0.70	1.00

Abbreviations; Cr, creatinine. *P* values were calculated using Student's t-test and p** values were calculated using Mann-Whitney U test as non-parametric analysis.*

4. With such a small sample size, the focus still seems to be on the inference or significance for the current version. However, its effect sizes might be more valuable than determining the significance.

Response: Thank you for this important suggestion. We have added discussion emphasizing the magnitude of effects rather than statistical significance in the discussion section.

Discussion (page 14):
Given the small sample size of this pilot study, the magnitude of observed effects may be more informative than statistical significance. The substantial increase in plasma D-serine levels (0.56 nmol/mL difference, 95% CI 0.07–1.05) and urinary D-serine-to-creatinine ratio (64.0 mmol/g Cr difference, 95% CI 20.8–107.2) represent clinically meaningful changes that warrant further investigation in larger studies. Similarly, the numerical increase observed in

plasma D-alanine levels and urinary D-alanine-to-creatinine ratio, while not reaching statistical significance, provide valuable preliminary data for effect size calculations in future trial designs.

Reviewer 2:

1. I think the background could benefit from some general information and references regarding the effects of antipsychotics on kidney function to help link your two topics here. A paragraph would be plenty to highlight current evidence regarding various antipsychotics and their influence on eGFR, CrCl, et

Response: Thank you for this valuable suggestion. We have added the sentences in the introduction section discussing the current evidence regarding various antipsychotics and their influence on kidney function.

Introduction (page 4-5):

Several studies have investigated the effects of antipsychotic medications on kidney function with mixed results. While some retrospective studies suggest that atypical antipsychotics, including risperidone, may not increase the risk of acute kidney injury compared to typical antipsychotics,[14, 15] other population-based studies have reported increased risks in elderly patients.[16] The mechanisms underlying these effects remain unclear, but may involve direct nephrotoxic effects or indirect effects through metabolic changes.

2. there needs to be several rationale given for methodological choices: why that duration for risp treatment? why sample size even if you did not perform a power calculation? why 140 as the cutoff for glucose which doesn't appear to rule out pre-diabetes? Why the significant instruction and concern for the effect of the microbiome which isn't mentioned in the background? This becomes even more important since there is a sudden introduction of microbiome analysis in the methods. This should be in the background with rationale for its study.

Response: We have added detailed rationale for our methodological choices throughout the methods section in the revised manuscript.

Introduction (page 5):

….Therefore, we conducted a pilot randomized controlled trial to investigate the impact of short-term risperidone administration on the amino acid profile, kidney function, and glucose metabolism in healthy adults. Microbiome analysis was included because gut microbiome can metabolize D-amino acids, and we aimed to control for potential confounding effects of microbial D-amino acid metabolizm on our study endpoints.

Methods, Study design and participants (page 6):

….The glucose cutoff was chosen to exclude participants with overt diabetes while including those with potential insulin resistance, as measured by HOMA-IR.

….Sample size calculation was not performed due to the absence of prior data on effect size. This pilot study was designed to generate preliminary data for future power calculations.

Methods, Interventions (page 6):
….The lowest available dosage of 0.5 mg/day was chosen to prioritize participant safety. The 4-day treatment duration was chosen based on pharmacokinetic studies showing that steady-state levels are achieved within 3-4 days, allowing us to observe steady-state effects while minimizing exposure in healthy volunteers.

3. You have some instruction about dietary restrictions in this study, was protein intake a concern given its potential effects on renal labs?

Response: Thank you for this important consideration. We did not specifically restrict protein intake in this study, which is a limitation. We have added this to the limitations section in the revised manuscript.

Discussion (page 15):
…. Fourth, we did not specifically control for protein intake during the study period, which may have influenced renal laboratory parameters and represents a limitation of our study design.

4. The focus of the background is on renal function but the primary endpoint was HOMA-IR? Is it possible to explain why this is the primary endpoint?

Response: We have clarified why HOMA-IR was chosen as the primary endpoint in the methods section.

Methods, Study endpoints (page 8):
The primary study endpoint was the mean change in HOMA-IR from baseline to 5 days between groups. HOMA-IR was selected as the primary endpoint because risperidone has the potential to increase glucose levels and insulin resistance, and we first aimed to assess the metabolic safety profile of risperidone in healthy individuals.

5. How and when were fecal samples collected for microbiome analysis?

Response: We have added detailed information about fecal sample collection procedures in the methods section.

Methods, Laboratory measurements (page 7):
Intestinal bacterial flora analysis was conducted based on 16S ribosomal ribonucleic acid (rRNA) sequencing of fecal samples using QIIME2 (version 2021.2) by Takara Bio's Biomedical Center (Shiga, Japan). Fecal samples were collected by participants at home using a collection kit (FS-0017, TechnoSuruga Lab. Co., Ltd., Shizuoka, Japan) at baseline and on day 5.

6. Did between group comparisons of mean changes correct for baseline values?

Response: We did not adjust for baseline values in our between-group comparisons. The baseline characteristics were generally comparable between the two groups, which reduced the necessity for baseline adjustment.

7. For the demographic/baseline comparisons between the groups, can you put statistical analyses to these statements of being comparable and the BMI "likely" being different? Were any identified differences controlled for in a sensitivity analysis to assess for potential effects on primary and secondary outcomes. Perhaps this should be included in the statistics of table 1?

Response: We have added statistical analyses to the demographic comparisons in Table 1 as mentioned in response to Reviewer 1, comment 1.

8. Table 1 footnote should explain what data is (%, means, s.d., etc).

Response: We have expanded the Table 1 footnote to explain the data presentation format.

Table 1 footnote (page 9-10):
Data are described as mean (standard deviation) *for continuous variables* or n (%) *for categorical variables*.

9. Is it possible to utilize the supplementary material to provide in-depth data of your microbiome analysis? The presentation of microbiome results is underwhelming given the breadth of data generally obtained from microbiome analysis.

Response: We agree that the microbiome analysis presentation could be enhanced and have added more detailed microbiome results to the supplementary material.

Results, Effects of risperidone on the fecal microbiome (page 12):
…. *Phylum-level analysis revealed that both groups were dominated by Firmicutes and Bacteroidetes, accounting for over 80% of the microbiome composition at baseline and no significant changes were observed in the composition at day 5 in either group (S2 Fig and S3 Table).*

S3 Table. Relative abundance of the fecal microbiome during follow-up in the risperidone and control groups

		**Risperidone (n = 3)**		**Control (n = 4)**	
	**Day**	**Mean (SD) relative abundance (%)**	**p**	**Mean (SD) relative abundance (%)**	**p**
Firmicutes	1	74.7 (4.0)	0.35	64.3 (6.7)	0.78
	5	68.5 (7.2)		65.9 (6.7)	

Bacteroidetes	1	16.2 (3.1)	0.65	21.3 (5.1)	0.28
	5	18.2 (5.2)		16.9 (4.0)	
Actinobacteria	1	7.2 (2.3)	0.16	8.2 (2.0)	0.18
	5	10.8 (1.7)		12.7 (4.7)	
Proteobacteria	1	1.9 (0.5)	0.67	5.4 (4.5)	0.56
	5	2.3 (1.1)		3.6 (1.9)	
Fusobacteria	1	0.0 (0.0)	–	0.8 (0.9)	0.81
	5	0.0 (0.0)		0.6 (0.7)	

10. The discussion is lacking in depth and detail, especially as it regards the microbiome analysis, why your renal findings might contradict that of other studies.

Response: We have substantially expanded the discussion section to provide more depth and detail, particularly regarding microbiome analysis and potential mechanisms explaining our findings.

Discussion (page 13-14):

…. Although fecal d-serine levels were not measured in the present study, we aimed to minimize confounding mediated dietary factors through the restriction of the intake of fermented foods, antibiotics, and lactic acid supplements during the study period. In addition, the lack of significant changes in gut microbiome diversity during the short treatment period suggests that the increases in D-serine levels were primarily due to direct enzyme inhibition rather than microbial effects. These findings ensured that the observed changes in d-serine were attributable to risperidone administration rather than the impact of dietary or gut microbiota-related factors.

….However, a population-based cohort study reported an increased risk of acute kidney injury-related hospitalizations in elderly patients treated with atypical antipsychotics, including risperidone.[16] The absence of acute adverse effects on kidney function in healthy participants in our study contrasts with some previous reports of antipsychotic-associated nephrotoxicity, which may be related to our study population (healthy individuals vs. patients with comorbidities) or the short duration of exposure. These discrepancies underscore the need for further studies to elucidate the kidney safety profile of risperidone particularly in vulnerable populations such as elderly individuals and patients with CKD.
